# Phenotypic Flexibility in the City: A Meta‐Analysis on Variation

**DOI:** 10.1111/ele.70419

**Published:** 2026-08-16

**Authors:** Jules Petit, Melanie Dammhahn

**Affiliations:** ^1^ Department of Behavioural Biology University of Münster Münster Germany; ^2^ Joint Institute for Individualisation in a Changing Environment (JICE) University of Münster and Bielefeld University Münster Germany

**Keywords:** environmental heterogeneity, individual variation, irreversible variation, meta‐analysis, phenotypic flexibility, phenotypic plasticity, reversible variation, urbanisation

## Abstract

Among global changes, urbanisation entangles a variety of human‐induced rapid environmental changes, such as habitat fragmentation, temperature change, introduction of human food sources and pollution. Urban environments in contrast to non‐urban ones are often assumed to be more heterogeneous and variable in space and time. A key feature of animals coping with high environmental variability ought to be phenotypic flexibility, that is, the capacity of individuals to express reversible variation in labile traits. However, this ‘phenotypic flexibility hypothesis’ has not been tested rigorously. We compiled available raw data and used a meta‐analysis to estimate overall differences in among‐ and within‐individual variation between urban and non‐urban population pairs of wild animals. We considered within‐individual variation as a proxy of phenotypic flexibility. Across all taxa, among‐individual variation did not differ between urban and non‐urban populations. Within‐individual variation was marginally lower in urban populations compared to non‐urban ones. The potential decrease of phenotypic flexibility in urban individuals could result from the multidimensionality and complexity of urban environmental conditions. Overall, the effects of urbanisation on phenotypic variation are not generalisable and depend on the taxa, species, and traits. Future studies should increase efforts to directly link temporal and spatial environmental variability with phenotypic individual variation.

## Graphical abstract

A key feature of animals coping with high environmental variability ought to be phenotypic flexibility. We used a meta‐analysis to estimate overall differences among‐ and within‐individual variation between urban and non‐urban population pairs of wild animals. We considered within‐individual variation as a proxy of phenotypic flexibility. Overall, the effects of urbanisation on phenotypic variation are not generalisable and may depend on the taxa, species and traits.

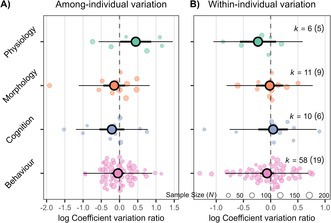

## 1 Introduction

Among the suite of global changes in environments, the impacts of urbanisation are particularly valuable to study as they encompass several human‐induced rapid environmental changes (HIREC) within a single area. Urbanisation alters habitat via loss and fragmentation, contributes to various forms of pollution (e.g., light, air and noise), and generates local warming via heat island effect (Grimm et al. 2008; Sih et al. 2011). These combined environmental alterations create a dynamic and complex system making cities a major multi‐level selection agent (Alberti 2015; Szulkin et al. 2020). Given that urban areas are expanding globally at an unprecedent rate exerting strong effects on wildlife, it is crucial to understand the factors that facilitate or constrain individual and population responses to urban environments. Reviews of empirical studies have reported major shifts in average phenotypic traits (i.e., a difference in population mean value of a trait) between urban and non‐urban populations (behaviour: Burkhard et al. 2026; life‐history: Capilla‐Lasheras et al. 2022a; physiology: Iglesias‐Carrasco et al. 2020; home range size: O’Donnell and delBarco‐Trillo 2020; morphology: Putman and Tippie 2020), as well as higher rates of phenotypic changes (Alberti et al. 2017). More recently, urban ecologists have studied how total phenotypic variation (observed total phenotypic variance of a trait) differ between urban and non‐urban populations (Burkhard et al. 2026; Capilla‐Lasheras et al. 2022a; Gervais et al. 2025). Observed phenotypic variance is a key component to understanding a population's ability to persist under changing environments, like urbanisation, as it represents the raw evolutionary material upon which natural selection is acting (Darwin 1859). Although assessing differences in total phenotypic variation is an important first step, more information can be gained by decomposing it into within‐ and among‐individual variation components (Araya‐Ajoy et al. 2023; Westneat et al. 2015).

Within‐individual variation is the amount of phenotypic variance attributable to differences in phenotype among observations of the same individual, and reflects reversible short‐term adjustments of individuals to transient environmental changes without genetic change (also referred to as intra‐individual variation, Piersma and Drent 2003). Within‐individual variation refers to the reversible phenotypic transformation an individual expressed for a given trait and can be considered a proxy of phenotypic flexibility, a specific form of phenotypic plasticity (Piersma and Drent 2003). Phenotypic flexibility is hypothesized to underly the success of urban dwellers when genetic changes cannot explain the speed of phenotypic changes within an individual lifetime (Hendry et al. 2008). High phenotypic flexibility is expected under fluctuating environmental conditions to facilitate an individual's phenotype‐environment match, like in urban habitats (Fox et al. 2019; March‐Salas et al. 2021; Matesanz et al. 2010; Nicotra et al. 2010; Pigliucci et al. 1996; Valladares et al. 2014). Urban habitats typically show spatial heterogeneity with high variation regarding the type and densities of buildings, or the presence of remnant and natural areas over short distances (Cadenasso et al. 2007; Pickett et al. 2001). Cities are also characterised by high spatial and temporal temperature fluctuations, especially in their centres, due to less greenery boosting the urban heat island effect (Soltani and Sharifi 2017). Lastly, the availability of anthropogenic food sources is assumed to fluctuate over shorter temporal scales compared to natural food sources (Stofberg et al. 2019). If this is true, the high environmental heterogeneity associated with city life in the aforementioned environmental attributes (Thompson et al. 2022) is expected to increase phenotypic flexibility of urban individuals in contrast to non‐urban conspecifics. Here, environmental heterogeneity is expected to have persistent, population‐level effect in both urban and non‐urban habitats, respectively. Surprisingly, we know only one study that tested whether higher spatial heterogeneity (i.e., approximated by variance in impervious surface area) was associated with greater phenotypic flexibility (i.e., within‐individual variation) and authors found no support for such relationships for breath rate and exploration in a passerine bird species (Gervais et al. 2025). Yet, even studies comparing solely phenotypic flexibility between urban and non‐urban populations—without estimates of environmental heterogeneity—are scarce, and produced mixed results hampering our abilities to understand the importance of reversible variation for adjustment to urban conditions and more broadly to heterogeneous habitats (but see Dammhahn et al. 2020; Gervais et al. 2025).

Urban environmental heterogeneity is not only expected to affect within‐individual phenotypic variation (i.e., phenotypic flexibility) but also between‐individual differences (Rivkin et al. 2019). Among‐individual variation is the amount of phenotypic variance attributable to differences between individuals in average phenotype and indicates individual specialisation (Araya‐Ajoy et al. 2023; Falconer and Mackay 1996; Nakagawa and Schielzeth 2010). Among‐individual variation represents, to some extent, irreversible variation generated by genetic and/or permanent environmental and developmental effects, revealing partly local adaptation and selective processes (Dingemanse and Wolf 2013; Lynch and Walsh 1998; Wilson et al. 2010). Changes in among‐individual variation have the potential to dampen the abilities of populations to cope with future environmental conditions when individuals in a population show high phenotypic homogenisation (Bolnick et al. 2011; Forsman and Wennersten 2016). In bird and predator communities, respectively, functional species homogenisation (i.e., a reduction of among‐species variation) is a well‐establish phenomenon in urban populations (Callaghan et al. 2019; Devictor et al. 2007; Silva et al. 2016; Sorace and Gustin 2009). To our knowledge, only Gervais et al. (2025) investigated differences in among‐individual variation between urban and non‐urban populations with mixed findings. Hence, we expect homogenisation to occur at the individual level similarly to the species level in urban populations, although we acknowledge the potential for context‐dependent effects in this process.

Within‐ and among‐individual variation can be estimated via variance decomposition using repeated measurement per individual, as commonly applied in quantitative genetics or animal personality to estimate heritability or repeatability, respectively (Falconer and Mackay 1996; Nakagawa and Schielzeth 2010; Réale et al. 2007). Disentangling these two variance components is essential to better understand the relative contribution of reversible and irreversible variation for adaptation to changing environmental conditions due to their genetic basis (Alonzo 2015; Martin et al. 2017). However, so far, most urban ecology meta‐analyses have summarised total phenotypic variation, lumping together both within‐ and among individual variation (behaviour: Burkhard et al. 2026; life‐history: Capilla‐Lasheras et al. 2022a).

Using a combined approach of data re‐analysis followed by a formal meta‐analysis, we aim to test predictions of the phenotypic flexibility and the homogenisation hypotheses. We first summarised studies with repeated individual measurements for both urban and non‐urban populations of wild non‐human animals for labile traits (i.e., behaviour, cognition, physiology, life‐history and morphology) assessed via standardised procedures. Although studies on clonal organisms have investigated phenotypic plasticity (Brans et al. 2017, 2018; Brans and De Meester 2018), they were not included in this meta‐analysis for reasons of comparability whenever they did not report repeated measurements per individual. Then, we quantified within‐ and among‐individual variation by performing variance decomposition on all available datasets. We tested whether habitat type (urban versus non‐urban) explained differences for each phenotypic variance component. Specifically, we tested two predictions (i) within‐individual variation is higher in urban compared to non‐urban populations (i.e., indicating higher phenotypic flexibility), and (ii) among‐individual variation is lower in urban compared to non‐urban populations (i.e., indicating phenotypic homogenisation).

It is important to note that within‐individual variation has two components, the individual's reaction norm (i.e., an individual's responsiveness to an environmental gradient, namely individual plasticity, Dingemanse et al. 2010; Snell‐Rood 2013) and the residual within‐individual variability (i.e., residual reversible variation after correcting for variation in average response and individual plasticity, namely predictability, Cleasby et al. 2015; Hertel et al. 2020; Westneat et al. 2015). Albeit these two components could be heritable and bear individual differences affecting both estimations of among‐ and within‐individual variation, we were not able to disentangle them due to statistical power limitations (Cleasby et al. 2015; Dingemanse et al. 2010; Westneat et al. 2015). Therefore, we assume that those individual differences are negligible in our results and discuss potential sources of biases they might cause.

## 2 Methods

### 2.1 Literature Review: Scoping, Search Term Development and Search

We used the Population, Exposure, Comparison and Outcome (PECO) framework to develop a search strategy. Our PECO framework has the following structure: *P* = among wild populations of non‐human animals, E = urban living conditions, C = non‐urban living conditions, O = within‐ and among‐individual phenotypic variation. We pre‐identified a few studies from our personal library with non‐specific query via Google Scholar to establish a preliminary query (Text S1). Then, we performed a pre‐screening on 1000 papers to refine and develop our final query for our search (Text S1). We performed the systematic literature search in five databases (Web of Sciences collection, Scopus, ProQuest, EBSCOhost Open Dissertations and OpenGrey) on the 10th of March 2023. The search allowed for the inclusion of published and unpublished literature (e.g., grey literature) across all continents and languages as well as personal communications. However, search terms were only used in English which might introduce some bias to the evidence base. The details of the search term used for each database can be found in Table S1. In total, we found 4322 papers. We removed 1150 duplicates using the software *Ryyan* (Ouzzani et al. 2016) and double checked with a conservative string match script in *R* (v.4.4.0; R Core Team 2022), resulting in 3172 papers. We performed three screening phases resulting in 113 papers. The first screening excluded all studies on humans, plants, domesticated or zoo animals, discarding 2626 studies. The second and third screenings excluded studies that did not have repeated individual measurements of labile traits for both urban and non‐urban populations, discarding 409 and 25 studies, respectively. Repeated measurements were defined as multiple measurements across contexts or over time per individual. Separate measurement per individual that were clones were not considered to fit our framework since such approach quantifies within‐genotype variation (another aspect of phenotypic plasticity) rather than within‐individual variation (specifically used here for phenotypic flexibility). The number of papers excluded for each screening phase is explained in Figure S1. All screening, data extraction, categorisation, and variance partitioning was done by Jules Petit. Hypotheses were set a priori, and analyses (with moderators) were planned prior to data collection.

### 2.2 Dataset Collection

To assess whether phenotypic flexibility (i.e., within‐individual variation) differs between urban and non‐urban populations, variance partitioning needed to be performed (for details see Section 2.4). None of the 113 papers did the adequate partitioning as most studies were not focusing on phenotypic flexibility. Therefore, raw data tables (raw values of phenotypic traits) were extracted if made open access, or authors were contacted to obtain raw data, or asked to run a specific *R*‐script (Text S5 for contact procedure). After the final contacting phase and a deep data check, we collected data from 33 studies (Dataset citation: Bar‐Ziv et al. 2022; Batabyal 2017; Batabyal and Thaker 2019a; Biondi 2021; Dominoni 2015, 2020; Garitano‐Zavala 2022; Hardman and Dalesman 2018a; Heppner 2023; Huang 2020; Jakubas et al. 2020a; Kaiser 2018, 2019, 2020; Kozlovsky et al. 2017a, 2017b, 2017c, 2017d; Mazza 2020; Nonacs et al. 2021; Ouyang et al. 2019a; Petit and Dammhahn 2024; Prasher et al. 2019b; Rimbach and Dammhahn 2024; Smit et al. 2023; Tabh et al. 2020; Thompson et al. 2018a; Tüzün et al. 2017a; Vardi and Berger‐Tal 2022a; Vincze 2014, 2016; Yovel 2020). Initially, the meta‐dataset contained 108 paired urban–non‐urban estimates from 23 species.

### 2.3 Data Categorisation

Labile traits are traits with the capacity to be reversibly expressed (Brommer 2013; Westneat et al. 2015). For behavioural, physiological, cognitive and life history traits, we assumed an overall presence of reversible variation (i.e., phenotypic flexibility). However, morphological traits can be labile, such as body mass, or not, such as tarsus length in birds, which grows continuously until a fixed size without possible reversible change. We excluded all morphological traits that were not labile. To standardise across studies, we relabelled traits following functional definitions coming from key papers (Table S2). The data categorisation was validated by five extra persons with relevant expertise.

The level of urbanisation can be assessed in multiple ways (Szulkin et al. 2020) and cities encompass various habitat types. To facilitate comparisons, we followed the classification into urban and non‐urban habitats by the authors of the original studies (Text S6). Although environmental heterogeneity exists within urban and non‐urban habitats respectively, we assumed that such within‐habitat variation (e.g., among non‐urban or among urban sites separately) was smaller than the between‐habitat variation (e.g., between non‐urban and urban overall habitat). Indeed, from the authors' descriptions, all studies had clear macro‐environmental changes associated with urbanisation, as urban locations had both higher human presence and higher coverage of built areas compared to the non‐urban ones. When studies did not report a clear urban and non‐urban classification (e.g., when study sites were distributed along an urban gradient), we asked the authors how they would categorise their sites.

Measurement intervals can affect both variance partitions via temporal bias of phenotypic variation assessment. Short‐interval measurements can either lower within‐individual variation due to individual's state similarity or inflate among‐individual variation when different individuals are measured under different environmental conditions (e.g., low versus high predation risk; Araya‐Ajoy et al. 2015, Dingemanse and Dochtermann 2013 for the pseudo‐repeatability). In addition, long‐term intervals could increase or decrease among‐individual variation due to developmental plasticity. Therefore, we classified measurement intervals for each trait into six categories (minute, day, hour, week, month and year) based on the shortest test interval. For example, if three tests were performed within 4 h over 7 days, we classified it as ‘hour’ interval. The biological meaning of different measurement intervals depends on the species' lifespan. Therefore, we created four temporal categories (very‐short, short, medium, and long) relating the interval between repeated measurements to one round of breeding season (more details in Text S7). Development (e.g., age class) can strongly affect phenotypic variation within a population (Sears 2014). To standardise comparisons and consider this potential effect, we subset datasets that had both juvenile and adult age classes. The category juvenile included age classes such as ‘juvenile’, ‘larvae’, ‘chick’ or ‘pupae’. The category adult included the age class named ‘adult’. Taxa might respond differently to urban conditions. Therefore, we classified every species according to their taxonomic class (e.g., mammal, bird, insect). We did not categorise more moderators related to the study sites (e.g., percentage imperviousness, urban heat island effect) or population characteristics (e.g., seasonality) due to data limitations and power issues.

### 2.4 Variance Partitioning

The two main objectives of the meta‐analysis were to assess whether within‐ and among‐individual variation in urban populations differ from non‐urban ones. For all traits, we partitioned the variance using linear mixed effects models (LMMs) to estimate both variance components. All models were run only on continuous or count data using a Gaussian assumption to facilitate the comparison between the estimates (Text S8 for justification). We used only untransformed data and excluded composite variables from PCAs because we were interested to estimate the raw amount of variance change. Any transformation could change the relationships among variances (Emerson 1991). Fitting count data in Gaussian statistic without transformation maintains the variance–mean relationship which we resolved by using logarithm coefficient variance ratio (lnCVR) estimates in the meta‐analysis (see below ‘Meta‐analytic effect size’), as suggested by Senior et al. (2020). We used the *R* package *lme4* to run all variance partitioning models (Bates et al. 2015).

The variance partitioning was based on the following steps. First, we ran ‘intercept‐only’ LMMs (IO‐LMMs) with only individual identity as random intercept. Second, we ran ‘adjusted’ LMMs (AD‐LMMs) with trial number, sex and treatment (if available) as fixed factors, among others, when the authors mentioned that they improved the model fit significantly. IO‐LMMs were used to perform basic variance partitioning and verify that the mean or variance estimation from the LMMs were aligned with the classical mean or variance estimation approach (Text S9). IO‐LMMs were also used for sensitivity analyses. AD‐LMMs were used to control for variation arising from experimental or natural causes (De Villemereuil et al. 2018). Within‐individual variation is composed of two components of each individual's reaction norm: individual plasticity (Dingemanse et al. 2010; Snell‐Rood 2013) and residual within‐individual variability (individual predictability; Hertel et al. 2020; Westneat et al. 2015). The compiled dataset did not allow for a proper estimation of individual random slopes, and we thus ran models without random slopes. The integration of non‐natural fixed factors (trial number and treatment) was an attempt to control for population mean plasticity related to experimental procedure which may introduce extra artificial variation, whereas natural fixed factors such as sex were included to allow a sex‐corrected assessment of the phenotypic flexibility hypothesis. Therefore, we consider AD‐LMMs to compute a more ecologically accurate variance partitioning, especially to estimate within‐individual variation. We used AD‐LMMs' estimated variance for the effect size in the main meta‐analytic models. We did not include interactions, quadratic effects, and more than three fixed effects as their variance estimation becomes difficult using a frequentist approach. For both model types, Maximum Likelihood method was used to estimate all variance components simultaneously, making variance estimates more comparable between models (by avoiding differential variance estimations when fixed factors are included) and better targeting the true variance parameter value (Bryk and Raudenbush 1992; Searle et al. 1992). In all models, among‐individual variation was approximated via the variance explained by the variable ‘ID_individual’. Within‐individual variation was approximated using the residual variance. Residual variance includes also measurement error. Among‐individual variance might still contain some sort of within‐individual variation (i.e., biased phenotypic flexibility) when repeated measures for an individual are taken in the same environment, but individuals differ in their environment (i.e., pseudo‐repeatability; Dingemanse and Dochtermann 2013). To mitigate such effect, we corrected our model with a moderator, taking into account the environmental test condition (treatment) when necessary.

### 2.5 Meta‐Analytic Effect Size

For all variance estimates that were validated, we calculated the log coefficient of variation ratio (*lnCVR*) to investigate differences in the variability between urban and non‐urban populations (Nakagawa et al. 2015; Senior et al. 2020). Mean and variance values are often positively associated (e.g., Taylor's Law; Cohen and Xu 2015), therefore, we chose *lnCVR* over *lnVR* as we did not mitigate the mean–variance relationship using log‐transformation on the count data. *lnCVR* is a widely used standardised metric allowing for the comparison of raw variance amounts from various measurements across studies while accounting for uncertainties due to sampling (i.e., sampling error variance). The *lnCVR* equation is defined as $\text{lnCVR}=\ln\left(\frac{{\mathrm{CV}}_{T}}{{\mathrm{CV}}_{C}}\right)+\frac{1}{2}\left(\frac{1}{n_{T}-1}-\frac{1}{n_{C}-1}\right)+\frac{1}{2}\left(\frac{s_{C}^{2}}{n_{C}{\bar{x}}_{C}^{2}}-\frac{s_{T}^{2}}{n_{T}{\bar{x}}_{T}^{2}}\right)$ where *ln* denotes the natural logarithm. ${\mathrm{CV}}_{T}=s_{T}/{\bar{x}}_{T}$ and ${\mathrm{CV}}_{C}=s_{C}/{\bar{x}}_{C}$ denote the coefficient of variation (*CV*) in the treatment group (i.e., urban) and control (i.e., non‐urban) group, respectively (see Senior et al. 2020, equation 12 for sampling error variance equation). To calculate *lnCVR* for among‐ and within‐individual variation separately, for both IO‐LMMs and AD‐LMMs with their associated sampling variance, we carried forward the among‐ and within‐individual variances. To do so, we used the variance components, ID_individual, and residuals from the LMMs, as well as the intercept from the basic *R* function *mean()* in R (v.4.4.0; R Core Team 2022). To compute *lnCVR*, we used the script from Nakagawa et al. (2015) as the package *metafor* (v.4.6‐0; Viechtbauer 2010) was not implemented with the mean–variance relationship correction for *lnCVR* sampling variance. Positive *lnCVR* values meant higher estimates for urban populations compared to non‐urban ones. Although our variance components were estimated with repeated measurements, we used the original sample size of the study for the calculation of the *lnCVR* to follow the most conservative approach. Using two sample size variants weighted for the number of repeated measurements did not change our findings (analyses not reported).

### 2.6 Final Dataset

**FIGURE 1:**
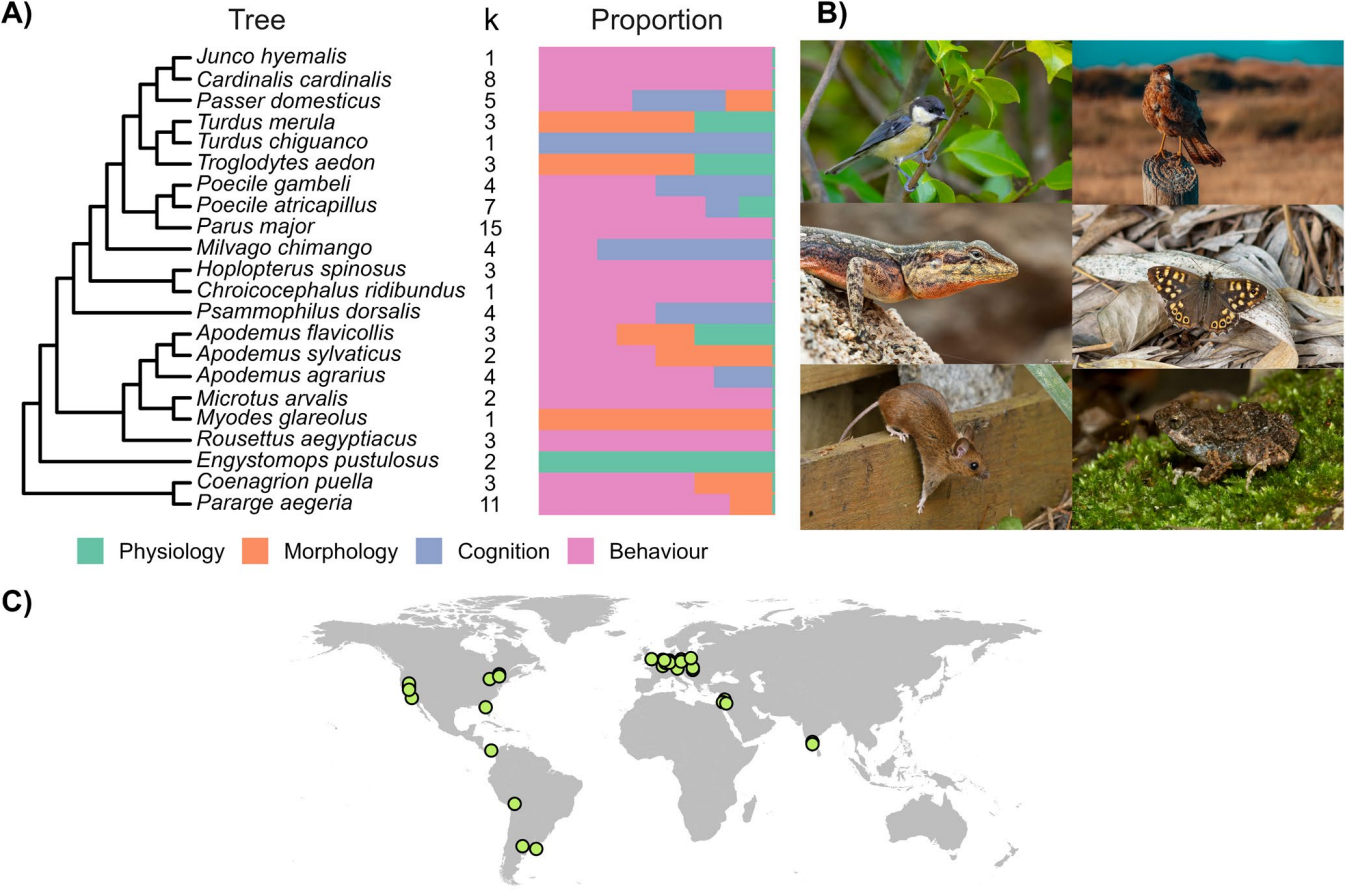
Phylogenetic and geographical portrayal of the meta‐analysis data set. (A) Phylogenetic tree of the 22 species included in the meta‐analysis with the number of effect sizes (k = urban–non‐urban comparisons) included per species (the numbers may vary if IO‐LMMs or AD‐LMMs are meta‐analysed, see ‘Final dataset’) and the proportion of comparisons for each phenotypic trait type. (B) Pictures of species included in the meta‐analysis representing the five major taxa. Left to right from top to bottom: *Parus major* , *Milvago chimango* , *Psammophilus dorsali*, *Pararge aegeria*, *Apodemus sylvaticus* , and *Engystomops pustulosus* . All images were extracted from www.flickr.com (Authors: Nicolas Venner, Gonzalo Arias, Vipin Baliga, Nicolas Venner, Tim Worfolk, Brian Gratwicke) in accord with their copyright (Text S11). (C) Global map (excluding Antarctica) showing the location of each study included in the meta‐analysis. Each point represents the urban location of the urban–non‐urban pair.

In total, 29 and 23 paired urban–non‐urban estimates were discarded for IO‐LMMs and AD‐LMMs respectively due to poor model fit or inability to calculate *lnCVR* (Text S9 for procedure and Tables S3 and S4 for summary of the deletion) including a complete removal of two studies (Grunst et al. 2014; Thompson and Morand‐Ferron 2019). The final meta‐dataset included 90 paired urban–non‐urban estimates from 22 species of 31 studies mainly located in the northern hemisphere (Figure 1; Bar‐Ziv et al. 2023; Batabyal et al. 2017; Batabyal and Thaker 2019b; Biondi et al. 2022; Dominoni et al. 2015, 2020; Garitano‐Zavala et al. 2022; Hardman and Dalesman 2018b; Harten et al. 2021; Heppner et al. 2023; Huang et al. 2020; Jakubas et al. 2020b; Kaiser et al. 2018, 2019, 2020; Kozlovsky et al. 2017e; Mazza et al. 2020; Mazza and Guenther 2021; Ouyang et al. 2019b; Papp et al. 2015; Prasher et al. 2019a; Smit et al. 2024; Solaro and Sarasola 2019; Stansell et al. 2022; Tabh et al. 2022; Thompson et al. 2018b; Tüzün et al. 2017b; Vardi and Berger‐Tal 2022b; Vincze et al. 2016; J. Petit & M. Dammhahn, personal communication; R. Rimbach & M. Dammhahn, personal communication). Phenotypic traits were divided into four phenotypic trait types: behaviour (61 effect sizes, 20 studies), physiology (6 effect sizes, 5 studies), cognition (12 effect sizes, 8 studies), and morphology (11 effect sizes, 9 studies). Within the 61 behavioural effect sizes, 15 corresponded to boldness (11 studies), 32 corresponded to activity/exploration (11 studies), 8 corresponded to aggression (3 studies), 1 corresponded to neophilia (1 study), 3 corresponded to neophobia (3 studies) and 2 corresponded to foraging (2 studies) (for sample size of IO‐LMMs and AD‐LMMs separately, see Text S10).

### 2.7 Phylogenies

We used the Open Tree of Life (Hinchliff et al. 2015; Rees and Cranston 2017) and the interface provided by the *R* package *rotl* (v.3.1.0; Michonneau et al. 2016; OpenTreeOfLife et al. 2019) to calculate the phylogenetic trees. We estimated tree branch length using Grafen's method (Grafen 1989) and generated a phylogenetic correlation matrix to include in all our multilevel meta‐analytic models. We assessed the phylogenetic signal based on the proportion of variation explained by phylogeny (Cinar et al. 2022).

### 2.8 Meta‐Analysis

We evaluated the effect of urbanisation on phenotypic flexibility of labile traits running phylogenetic multilevel (intercept‐only) meta‐analysis models for each response term (Model 1 and Model 3, Table 1). We also fitted (no‐intercept) models including trait type as the response variable to estimate a separate coefficient for behaviour, physiology, morphology and cognition, respectively (Model 2 and Model 4, Table 1). One recent study found that different phenotypic traits might show different change across the variance partition (Gervais et al. 2025). Thus, we ran a (no‐intercept) model including only behavioural traits as a response variable since it had an appropriate sample size to estimate a separate coefficient for boldness, aggression, and activity/exploration (Model 5, Table 1). Since a large part of variability across studies included in our meta‐analysis (heterogeneity) was present among our effect size, we tested whether the among‐ and within‐individual variance differences between urban and non‐urban populations of the labile traits were affected by additional a priori selected moderators. We fitted a separate (no‐intercept) model for each of the following predictors: provenance of the data (whether wild animals are measured in the wild, in the lab or both), the temporal category of interval between the repeated measurement corrected for the species lifespan (inter‐test interval corrected for lifespan; short, very short), the age class (adult, juvenile), the taxa (amphibian, reptile, bird, mammal, insect), and the number of random and fixed factors used in the LMM to partition the variance (Model 6a to 6e and Model 7a to 7e, Table 1). We did not run these models with interactions across moderators due to data and power limitation.

**TABLE 1:** Summary of the different models present in the meta‐analysis.

Model ID	Response	Variance partitioning model	Data	Moderators	Details
*Main analyses*					
1	lnCVR among‐individual variation	AD‐LMM	All traits	Intercept	Overall meta‐analysis on among‐individual variation. Univariate. Table S5. Figure S2
2	lnCVR among‐individual variation	AD‐LMM	All traits	Trait type	Effect per trait on among‐individual variation. Quadrivariate. Table S7. Figure 2A
3	lnCVR within‐individual variation	AD‐LMM	All traits	Intercept	Overall meta‐analysis on within‐individual variation. Univariate. Table S6. Figure S3
4	lnCVR within‐individual variation	AD‐LMM	All traits	Trait type	Effect per trait on within‐individual variation. Quadrivariate. Table S8. Figure 2B
5	lnCVR within‐individual variation	AD‐LMM	Only behaviour	Behaviour trait type	Effect per behavioural trait on within‐individual variation. Trivariate. Table S9. Figure 3
*Secondary analyses*					
6.a	lnCVR among‐individual variation	AD‐LMM	All traits	Provenance of data	Effect of a specific moderator on among‐individual variation. Multivariate. Table S10a
6.b	lnCVR among‐individual variation	AD‐LMM	All traits	Interval between measurement corrected for lifespan	Effect of a specific moderator on among‐individual variation. Multivariate. Table S10b
6.c	lnCVR among‐individual variation	AD‐LMM	All traits	Age class	Effect of a specific moderator on among‐individual variation. Multivariate. Table S10c
6.d	lnCVR among‐individual variation	AD‐LMM	All traits	Taxa	Effect of a specific moderator on among‐individual variation. Multivariate. Table S10d
6.e	lnCVR among‐individual variation	AD‐LMM	All traits	Number of fixed and random effect in AD‐LMM	Effect of a specific moderator on among‐individual variation. Multivariate. Table S10e
7.a	lnCVR within‐individual variation	AD‐LMM	All traits	Provenance of data	Effect of a specific moderator on within‐individual variation. Multivariate. Table S12a
7.b	lnCVR within‐individual variation	AD‐LMM	All traits	Interval between measurement corrected for lifespan	Effect of a specific moderator on within‐individual variation. Multivariate. Table S12b
7.c	lnCVR within‐individual variation	AD‐LMM	All traits	Age class	Effect of a specific moderator on within‐individual variation. Multivariate. Table S12c
7.d	lnCVR within‐individual variation	AD‐LMM	All traits	Taxa	Effect of a specific moderator on within‐individual variation. Multivariate. Table S12d
7.e	lnCVR within‐individual variation	AD‐LMM	All traits	Number of fixed and random effect in AD‐LMM	Effect of a specific moderator on within‐individual variation. Multivariate. Table S12e
*Sensitivity analysis*					
8	lnCVR among‐individual variation	IO‐LMM	All traits	Intercept	Overall meta‐analysis on among‐individual variation. Univariate. Table S13a
9	lnCVR among‐individual variation	IO‐LMM	All traits	Trait type	Effect per trait on among‐individual variation. Quadrivariate. Table S13b
10	lnCVR within‐individual variation	IO‐LMM	All traits	Intercept	Overall meta‐analysis on within‐individual variation. Univariate. Table S14a
11	lnCVR within‐individual variation	IO‐LMM	All traits	Trait type	Effect per trait on within‐individual variation. Quadrivariate. Table S14b
12	lnCVR within‐individual variation	IO‐LMM	Only behaviour	Behaviour trait type	Effect per behavioural trait on within‐individual variation. Trivariate. Table S14c
13	lnCVR among‐individual variation	AD‐LMM	All traits with only bird	Intercept	Overall meta‐analysis on among‐individual variation only with bird. Univariate. Table S15a
14	lnCVR within‐individual variation	AD‐LMM	All traits with only bird	Intercept	Overall meta‐analysis on within‐individual variation only with bird. Univariate. Table S15b
15	lnCVR among‐individual variation	AD‐LMM	All traits without bird	Intercept	Overall meta‐analysis on among‐individual variation without bird. Univariate. Table S15a
16	lnCVR within‐individual variation	AD‐LMM	All traits without bird	Intercept	Overall meta‐analysis on within‐individual variation without bird. Univariate. Table S15b
17	lnCVR among‐individual variation	AD‐LMM	All traits	Intercept	Single study effect. Univariate. Table S16a
18	lnCVR within‐individual variation	AD‐LMM	All traits	Intercept	Single study effect. Univariate. Table S16b
*Publication bias*					
19	lnCVR among‐individual variation	AD‐LMM	All traits	Square‐root of the inverse of the Effective sample size	Overall small‐study effect on among‐individual variation. Table S17a
20	lnCVR within‐individual variation	AD‐LMM	All traits	Square‐root of the inverse of the effective sample size	Overall small‐study effect on within‐individual variation Table S17b
21	lnCVR among‐individual variation	AD‐LMM	All traits	Mean‐centred year of study publication	Overall decline effect on among‐individual variation. Table S18a
22	lnCVR within‐individual variation	AD‐LMM	All traits	Mean‐centred year of study publication	Overall decline effect on within‐individual variation Table S18b

All meta‐analytic models estimated four random intercept effects, publication identity (i.e., among‐study variation), phylogeny (see ‘phylogenies’ section), species identity (i.e., among‐species variation not explained by phylogeny) and an observation ID term (residuals). All models were fitted assuming compound symmetry variance structure. For all models, we estimated total heterogeneity (*I* ^2^) following Nakagawa and Santos (2012) and Senior et al. (2016) using the *R* function *i2_ml* (*orchaRd R* package v.2.0; Nakagawa et al. 2021). We considered *I* ^2^ values around 25%, 50% and 75% as low, moderate and high heterogeneity, respectively (Higgins et al. 2003). Along with estimates, we reported 95% confidence intervals (CI) and 95% prediction intervals (PI) using the function *predict.rma* (*metafor* v.4.6‐0; Viechtbauer 2010). We performed all analysis and produced visualisations using *R* (v.4.4.0; R Core Team 2022) and the code available in the paper of Capilla‐Lasheras et al. (2022a); code citation: Capilla‐Lasheras et al. (2022b).

### 2.9 Sensitivity Analysis

We assessed the robustness of our results with three complementary analyses. First, we re‐ran the same models as Model 1 to 5 using *lnCVR* calculated from the variance component of IO‐LMMs (Model 8 to 12, Table 1). Since bird taxa represented 61% of our effect sizes, we then re‐ran the phylogenetic multilevel (intercept‐only) meta‐analyses (Model 1 and 3, Table 1) only on birds (Model 13 and 14, Table 1) and excluding all bird species (Model 15 and 16, Table 1). We did not re‐run models using *lnVR* because of the strong mean–variance relationship present in our study due to the count data. Lastly, we performed two leave‐one‐out analyses testing the effect of a single study on the overall *lnCVR* estimates of among‐ and within‐individual variation, respectively (Model 17 and 18, Table 1).

### 2.10 Publication Bias

We assessed small‐study bias and decline effects (time‐lag effects), following Nakagawa et al. (2022) method and the examples from Capilla‐Lasheras et al. (2022a). In total, we ran four additional uni‐moderator multilevel meta‐analytic models for *lnCVR* of among‐ and within‐individual variation, respectively (Model 19 to 22, Table 1). Each of these models included a single moderator: either the square‐root of the inverse of the effective sample size or the mean‐centred year of study publication (Nakagawa et al. 2022; Trikalinos and Ioannidis 2005). When data were unpublished, we used the year when the data were collected in the model. The variation explained by these moderators (i.e., *R* ^2^_marginal_) was calculated using the *R* function *r2_ml* (*orchaRd R* package v.2.0; Nakagawa et al. 2021).

## 3 Results

### 3.1 Is Among‐Individual Variation Lower in the City?

**FIGURE 2:**
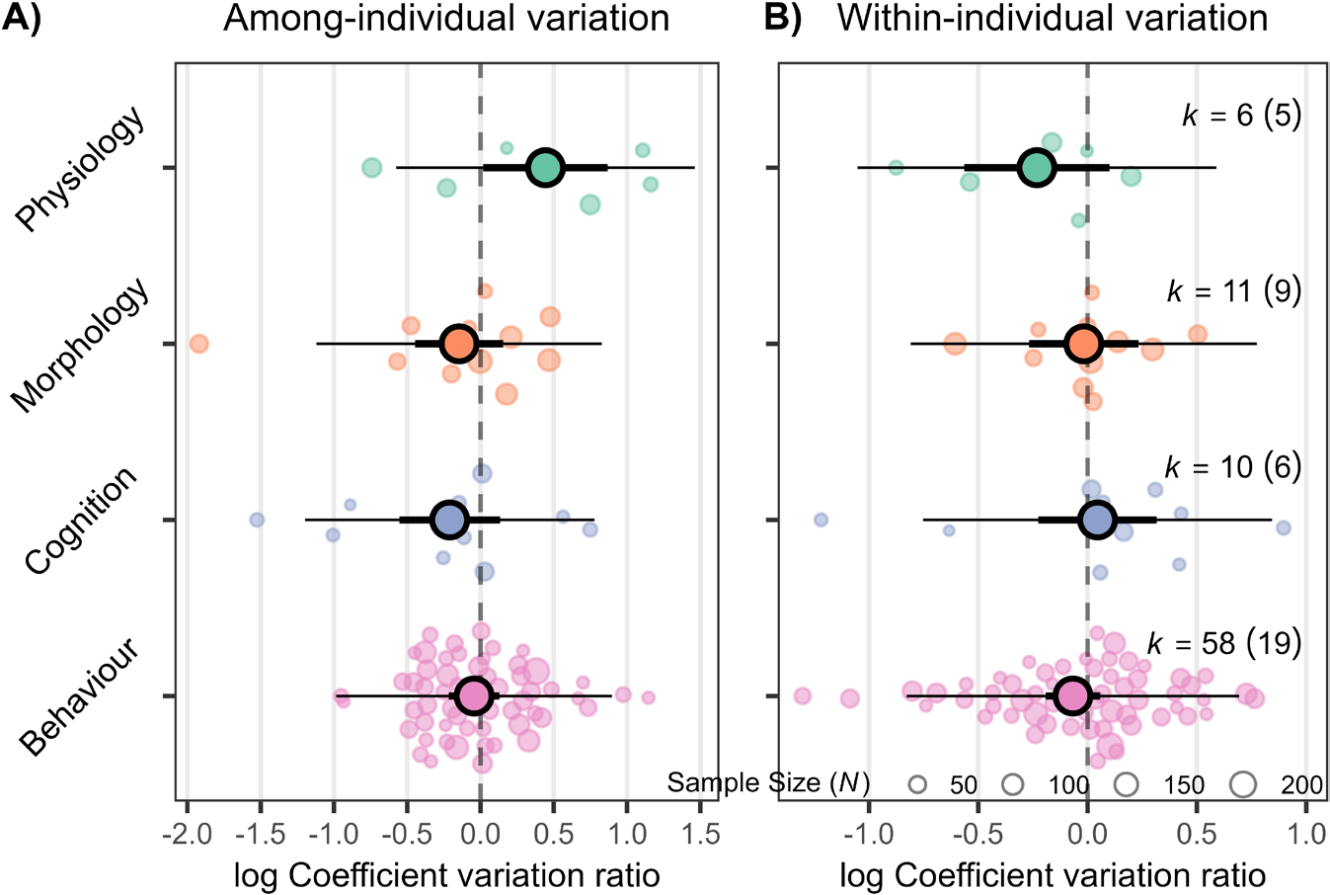
Meta‐analytical model estimates (log coefficient of variation ratio; *lnCVR*) per phenotypic trait assessing differences between urban and non‐urban populations for (A) among‐individual variation (Model 2) and (B) within‐individual variation (Model 4) calculated from AD‐LMMs. Positive values on the *x*‐axes represent higher among‐ or within‐individual variation in urban populations compared to non‐urban populations and *vice versa* for negative values. The large coloured points represent overall meta‐analytical model estimates. The thick black lines show their 95% confidence intervals. The thin black line shows the 95% prediction intervals. Transparent small coloured dots show the raw data (their size is scaled according to their sample size from which they were estimated). *k* is the number of observations supplemented with the number of studies between brackets.

*Univariate and Quadrivariate Meta‐Analytical Model (Origin of Variance: AD‐LMMs*). The overall coefficient of among‐individual phenotypic variation in urban populations did not differ from non‐urban ones (Model 1: *lnCVR* estimate [95% CI], [95% PI] = −0.031 [−0.174, 0.112], [−0.972, 0.910], Figure S2, Table S5). Total heterogeneity was high (*I* ^2^_total_ = 89.8%), with 17.9% explained by species‐specific effects (Table S5). Calculating urban effects per trait indicated that physiological traits seemed to respond differently to urbanisation for among‐individual variation compared to other phenotypic traits (Model 2: *lnCVR* behaviour estimate [95% CI], [95% PI] = −0.044 [−0.217, 0.129], [−0.985, 0.898]; cognition estimate = −0.210 [−0.554, 0.134], [−1.197, 0.777]; morphology estimate = −0.146 [−0.446, 0.155], [−1.119, 0.827]; physiology estimate = 0.444 [0.019, 0.869], [−0.575, 1.462], Figure 2A, Table S7). However, we note that the ‘physiology’ estimate may not be stable due to its low number of compiled effect sizes (*n* = 6). Total heterogeneity was high (*I* ^2^_total_ = 89.7%), with 6.0% and 19.4% explained by species‐ and study‐specific effects, respectively (Table S7).

### 3.2 Is Within‐Individual Variation Higher in the City?

**FIGURE 3:**
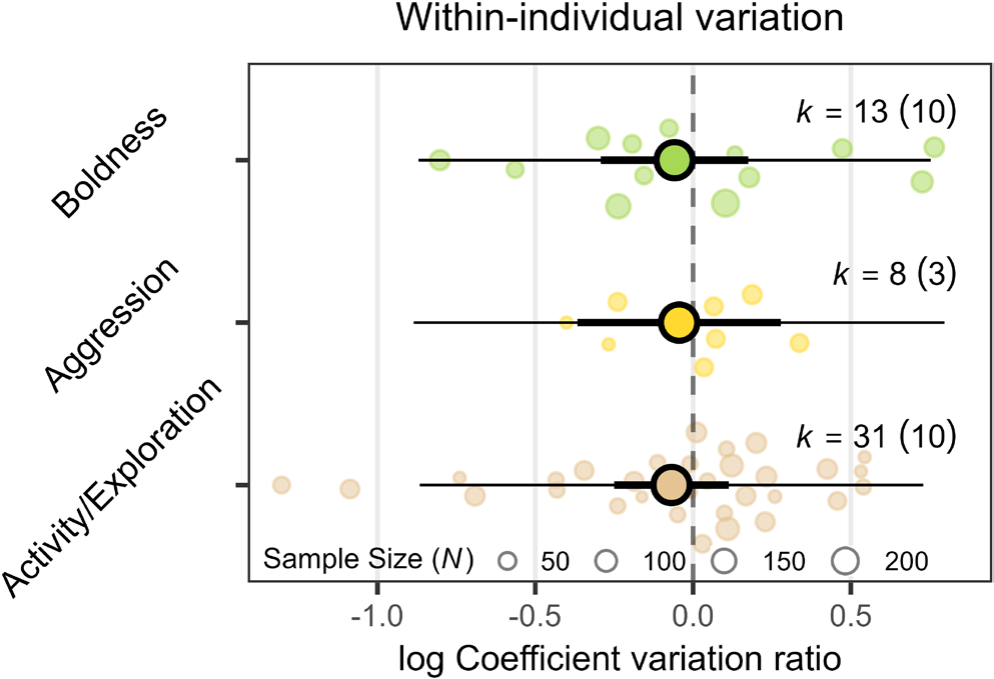
Meta‐analytical model estimates (log coefficient of variation ratio; *lnCVR*) per behaviour assessing differences between urban and non‐urban populations for within‐individual variation calculated from AD‐LMM (Model 5). Positive values on the *x*‐axis represent higher within‐individual variation in urban populations compared to non‐urban populations and *vice versa* for negative values. The large coloured points represent overall model estimates. The thick black lines show their 95% confidence intervals. The thin black line shows the 95% prediction intervals. Transparent small coloured dots show the raw data (their size is scaled according to their sample size from which they were estimated). *k* is the number of observations supplemented with the number of studies between brackets.

*Univariate and Quadrivariate Meta‐Analytical Model (Origin of Variance: AD‐LMMs)*. We found that non‐urban populations tended to have on average 5.9% higher coefficients of within‐individual phenotypic variation than urban populations, but this effect did not differ from zero (Model 3: *lnCVR* estimate [95% CI], [95% PI] = −0.059 [−0.159, 0.041], [−0.808, 0.690], Figure S3, Table S6). Total heterogeneity was high (*I* ^2^_total_ = 92.5%), with 6.9% explained by species‐specific effects (Table S6). Calculating urban effects per trait suggested that the observed trend was most likely due to a pattern emerging from behavioural traits (Model 4: *lnCVR* behaviour estimate [95% CI], [95% PI] = −0.067 [−0.192, 0.057], [−0.828, 0.694]; cognition estimate = 0.045 [−0.226, 0.316], [−0.753, 0.844]; morphology estimate = −0.017 [−0.268, 0.233], [−0.809, 0.774]; physiology estimate = −0.232 [−0.564, 0.100], [−1.053, 0.589], Figure 2B, Table S8). Total heterogeneity was high (*I* ^2^_total_ = 92.7%), with 8.14% explained by species‐specific effects (Table S8). Additional analysis on the subset of behavioural traits revealed that there was not a specific behaviour leading the increase of within‐individual variation in non‐urban populations compared to urban ones (Model 5: *lnCVR* activity/exploration estimate [95% CI], [95% PI] = −0.068 [−0.250, 0.113], [−0.866, 0.730]; aggression estimate = −0.044 [−0.366, 0.278], [−0.885, 0.797]; boldness estimate = −0.059 [−0.293, 0.176], [−0.870, 0.753], Figure 3, Table S9). Total heterogeneity was high (*I* ^2^_total_ = 93.9%), with 12.9% explained by species‐specific effects (Table S9).

### 3.3 Are Other Moderators Explaining Heterogeneity for Among‐ and Within‐Individual Variation in the Context of Urbanisation?

*Meta‐Regression Model (Origin of Variance: AD‐LMMs)*. We found a positive correlation between the number of factors added in the AD‐LMMs used for variance partitioning and the *lnCVR* of among‐individual variation (Model 6e: *lnCVR* ‘Number of factors in AD‐LMM’ estimate [95% CI] = 0.223 [0.081, 0.366], Table S10e). Provenance of data, inter‐test interval corrected for lifespan, age class and taxa did not affect *lnCVR* of among‐individual variation (Table S10a–d). We ran an additional analysis to understand why the number of factors affected the *lnCVR* of among‐individual variation, but it was inconclusive (see Text S12 and Table S11). For *lnCVR* of within‐individual variation, we found a trend that short inter‐test intervals corrected for lifespan increased the contrast between urban and non‐urban populations for within‐individual variation, where non‐urban populations displayed higher within‐individual variation (Model 7b: *lnCVR* ‘inter‐test interval corrected for lifespan_SHORT’ estimate [95% CI] = −0.157 [−0.341, 0.028], Table S12b) which does not seem to be the case for very short inter‐test interval (Model 7b: *lnCVR* ‘inter‐test interval corrected for lifespan_VERYSHORT’ estimate [95% CI] = −0.021 [−0.132, 0.090], Table S12b). We did not find any effect of the provenance of data, class age, taxa and the number of factors added in the AD‐LMMs used for variance partitioning on *lnCVR* of within‐individual variation (Table S12a,c–e, respectively).

### 3.4 Sensitivity Analysis and Assessment of Publication Bias

#### 3.4.1 Sensitivity Analysis—IO‐LMMs Versus AD‐LMMs

We performed the same main analysis (Model 1 to Model 5) using the effect size calculated from the variance of the IO‐LMMs (Text S13). Overall, we noted that *lnCVR* estimates might change between IO‐LMM and AD‐LMM methods but that all estimates fell within the confidence interval of each other (*lnCVR* among‐individual: Table S13, *lnCVR* within‐individual: Table S14). Only the estimates of physiological and cognitive traits from the meta‐analytical model of *lnCVR* on among‐individual variation had noticeable changes in the estimates probably due to the low sample sizes for those specific traits (Tables S13b and S14c). Most importantly, the overall observed patterns remained.

#### 3.4.2 Sensitivity Analysis—Bird Versus All Other Taxa Combined

We conducted the main meta‐analysis (Model 1 and Model 3) only on the bird data (Model 13 and Model 14) and excluding the bird taxa (Model 15 and Model 16). For among‐individual variation, we found that non‐bird taxa of non‐urban populations tended to have on average 4.3% higher coefficient of among‐individual phenotypic variation than non‐bird taxa of urban populations, but the 95% confidence interval for this estimate overlapped zero (Model 15: *lnCVR* estimate [95% CI] = −0.043 [−0.215, 0.128], Table S15a for comparisons). We did not find this trend in the bird taxa analysed alone (Model 14: *lnCVR* estimates [95% CI] = −0.029 [−0.299, 0.240], Table S15a for comparisons). For within‐individual variation, we found the same trend as in the main analysis whether the analysis was performed only on birds (Model 14: *lnCVR* estimate [95% CI] = −0.068 [−0.199, 0.063], Table S15b for comparisons) or only on non‐bird taxa (Model 16: *lnCVR* estimate [95% CI] = −0.056 [−0.211, 0.101], Table S15b for comparisons).

#### 3.4.3 Sensitivity Analysis—Single Study Effect

We did not find evidence for the existence of a large influence of a single study on the overall *lnCVR* estimates of among‐ and within‐individual variation (Model 17 and 18, Table S16a,b, respectively).

#### 3.4.4 Publication Bias—Small‐Study and Decline Effects

We did not find evidence for the existence of small‐study or decline effects in our study (Model 19 to 22, Text S14, Tables S17 and S18 respectively).

## 4 Discussion

We used phylogenetically controlled multilevel meta‐analysis to assess how urban living is related to changes in phenotypic variation for labile phenotypic traits. By decomposing phenotypic variation into within‐ and among‐individual variation, we addressed potential changes in reversible and irreversible variation between urban and non‐urban populations. Contrary to the phenotypic flexibility hypothesis, we found no evidence that urban individuals are more flexible than non‐urban ones. Instead, we observed a weak tendency for urban individuals to be less flexible than non‐urban ones. There was also no evidence for urban individuals being more phenotypically similar to each other than non‐urban ones. However, when excluding birds, we found weak evidence for this predicted pattern. Our findings highlight that the effects of urbanisation on the different partitions of phenotypic variation are not as straightforward and generalisable as expected and may depend on the taxa, species, and traits.

### 4.1 Testing the Phenotypic Flexibility Hypothesis for Urban Environments

Cities are characterised by high spatial variation in their landscape structure (Cadenasso et al. 2007; Pickett et al. 2001), high temporal change of anthropogenic food availability (Stofberg et al. 2019), and high spatial–temporal temperature fluctuations (Soltani and Sharifi 2017). In this context of rapid environmental changes, the capacity for individuals to reversibly adjust their phenotype has been suggested to be key for adaptation. The phenotypic flexibility hypothesis posits that exposure to higher environmental fluctuations creates higher reversible variation in individual's trait response. However, we found that across taxa, urban individuals did not express higher phenotypic flexibility (i.e., within‐individual variation) than non‐urban counterparts. On the contrary, we observed a weak tendency for urban individuals to express lower phenotypic flexibility, especially in their behaviour. It is possible that the assumption associating urban habitats with higher environmental heterogeneity might not be generalisable (Thompson et al. 2022). Cities might be more variable than non‐urban areas in some environmental attributes, but most likely not all, like in the case of day to day minimum and maximum temperature, due to the urban heat island effect (Tam et al. 2015). In addition, the patterns predicted by the phenotypic flexibility hypothesis will also depend on the ecological relevance of the traits in the environment in focus. Here, we assumed similar relevance for all traits across the compiled studies, however such findings should be further confirmed.

### 4.2 Environmental Heterogeneity Effect in the City

Environmental heterogeneity measures are generally lacking in empirical studies, limiting our ability to assess the role of total phenotypic variation, or more specifically, phenotypic flexibility in relation to such heterogeneity. To our knowledge, only two studies have directly linked spatial heterogeneity to change in phenotypic variation (total phenotypic variation: Capilla‐Lasheras et al. 2022a; within‐individual variation: Gervais et al. 2025) with mixed findings in the context of urbanisation. More importantly, Gervais et al. (2025) revealed that spatial heterogeneity might not follow the urbanisation gradient as expected. In our study, we were not able to extract enough spatial heterogeneity information for a meaningful comparison. Most critically, it would have been difficult, if not impossible, to decide a ‘one‐size‐fits‐all’ species scale. Urban environments are complex systems with multiple environmental axes (Szulkin et al. 2020), which may have similar or different heterogeneity compared to natural environments, depending on the measurement scale, the species in focus, and the ecological conditions (Alberti et al. 2020; Pickett et al. 2016; Uchida et al. 2021). In fact, this environmental complexity as well as crypticity does not exclude the possibility that non‐urban habitat could have higher environmental heterogeneity, with non‐urban populations expressing higher phenotypic flexibility under certain contexts. In addition, urban individuals could choose to live in areas of the city that display low environmental heterogeneity to reduce environmental pressures (i.e., ‘selection of the environment’ in Edelaar and Bolnick 2019; ‘niche choice’ in Trappes et al. 2022). Therefore, such variety of processes associated with the environmental heterogeneity effect could explain why we do not observe an overall difference in phenotypic flexibility between urban and non‐urban habitats in our study. Overall, our meta‐analysis suggests (a) a lack of support for the fundamental prediction of higher phenotypic flexibility in the city and (b) to be cautious regarding the assumption of higher environmental variation in the urban environment compared to non‐urban ones as it is rarely assessed. Future studies should measure and report environmental heterogeneity in concert with trait variation along urbanisation gradients to allow for more nuanced tests of the phenotypic flexibility hypothesis explaining adaptation of urban dwellers.

### 4.3 Phenotypic Flexibility and Environmental Heterogeneity: The Role of Individual Plasticity and Predictability

In our meta‐analysis, we quantified and contrasted within‐individual variation between urban and non‐urban populations to partly estimate changes in reversible phenotypic flexibility due to urbanisation. Contrary to the expectation, we found a weak trend that urban populations express lower phenotypic flexibility, especially in their behaviour. Within‐individual variation is composed of two components of each individual's reaction norm: individual plasticity (Dingemanse et al. 2010; Snell‐Rood 2013) and residual within‐individual variability (individual predictability; Hertel et al. 2020; Westneat et al. 2015). As we were unable to statistically disentangle these components due to lack of information, we assumed they reacted the same to environmental heterogeneity. However, most likely each component has different independent responses to environmental heterogeneity.

Concerning individual plasticity, urban individuals might express weaker plastic responses when changes in the urban environment are non‐detrimental to reduce the costs of plasticity (DeWitt et al. 1998). Mechanisms often studied in the urban context such as environmental insensitivity (e.g., behavioural tolerance: Čapkun‐Huot et al. 2024; habituation: Blumstein 2016) and environmental non‐assimilation (Kelley et al. 2018) could drive a reduction of individual plasticity, implying that individuals show different reversible variation decoupled from the overall environmental fluctuations they face. To our knowledge, only one study from Sprau and Dingemanse (2017) in great tits ( *Parus major* ) assessed individual plasticity in the context of urbanisation and found that individuals did not plastically adjust their aggressiveness and risk‐taking behaviour in response to changes in human activity, a proxy of urbanisation. We note that the impossibility to estimate individual plasticity in our study might have either increased or decreased within‐ and among‐individual variance by neglecting potential fanning out or fanning in of individual reaction norms. However, we believe that the short intervals between measurements should buffer such effects.

Changes in individual predictability (i.e., residual within‐individual variability) can be produced by different mechanisms linked to environmental heterogeneity. First, predictability could decrease via additive effects of multidimensional reaction norms (Westneat et al. 2015) involving an environmental factor, reducing variability in the city (opposite of our prediction, e.g., lower day to day minimal and maximal temperature in the city, Tam et al. 2015). Second, if we assume that an individual's reaction norm slope is the optimal response to environmental change, then individuals expressing high residuality around this slope would be considered far from their optimal response (assessment error in plasticity) and express maladaptive phenotypic variation. Since urban filter strongly selects certain phenotypes (behaviour: Burkhard et al. 2026; life‐history: Capilla‐Lasheras et al. 2022a; home range size: O’Donnell and delBarco‐Trillo 2020; morphology: Putman and Tippie 2020) such selection could result in urban individuals characterised by lower assessment error than non‐urban individuals explaining why urban individuals tend to show lower phenotypic flexibility.

Overall, our results highlight that the assumed effects of urban heterogeneity on phenotypic flexibility might not be generalisable across cities and phenotypic traits. The presence of differential feedback mechanisms between individual plasticity and individual predictability may create high variability in how urbanisation and phenotypic flexibility are linked with each other. More detailed data sets are required to better understand these fascinating processes.

### 4.4 Does Among‐Individual Variance Decrease in the City?

Urban environments favour certain phenotypes, leading to shifts in population averages (behaviour: Burkhard et al. 2026; life‐history: Capilla‐Lasheras et al. 2022a; home range size: O’Donnell and delBarco‐Trillo 2020; morphology: Putman and Tippie 2020). However, it is unclear how this pattern affects among‐individual variation within populations. Urban habitats are known to select mostly generalist, thermophilic or high‐dispersal capacity species, reducing the number of urban living species (Callaghan et al. 2019; Piano et al. 2017; Silva et al. 2016; Sorace and Gustin 2009) and creating functional species homogenisation (Devictor et al. 2007). Indeed, high intraspecific competition can increase among‐individual niche variation when it limits the use of an optimal resource, leading individuals to feed on alternative resources (Svanbäck and Bolnick 2005, 2006). As an individual's resource use is linked to phenotypic trait variation (Bolnick et al. 2011) and intraspecific competition could act similarly as interspecific competition, we assumed a similar pattern might be possible for among‐individual trait variation and interspecific competition. Therefore, we predicted that reduced interspecific competition in these impoverished communities could decrease among‐individual variation in the city. Surprisingly, we did not find that urban individuals were more similar to each other than non‐urban ones, most likely due to context‐dependent effects in this process (supporting mixed findings of Gervais et al. 2025). This equivocal result could in theory arise from several important processes acting in different directions. Although we have limited evidence to distinguish them, it is worth enumerating them so future studies can gather the requisite data. First, reduction of species richness along urbanisation gradients (Szabó et al. 2023; however, see Rimbach et al. 2025) could decrease interspecific competition and favour individual specialisation, increasing among‐individual variation. However, population density is often higher in cities (Batáry et al. 2018; Szabó et al. 2023), increasing intraspecific competition which may counterbalance any interspecific competition reduction, maintaining similar levels of among‐individual variation. Second, dispersal propensity of the taxa could create a differential response to urban spatial fragmentation. Species with high dispersal propensity, such as birds or flying insects, might experience higher gene flow (Medina et al. 2018; Miles et al. 2019; but see Delaney 2013) or colonise more diverse urban locations (Thompson et al. 2025, see hypothesis H1), maintaining similar among‐individual variation between urban and non‐urban populations. Species with low dispersal ability might endure higher selective pressures under urban conditions than their non‐urban counterparts, canalising individual's phenotype towards the population optimum average, reducing among‐individual variation in urban populations (Dingemanse and Réale 2005). Results of our sensitivity analysis, indeed, suggest that urban bird populations have similar among‐individual variation to non‐urban ones, whereas urban populations of other taxa than birds tended to have reduced among‐individual variation compared to non‐urban ones. Overall, our results regarding among‐individual variation highlight a potential for phenotypic homogenisation across cities due to an urban filter, although the effects are most likely taxa‐ or species‐specific.

## 5 Conclusions

In our evidence synthesis, we compiled data on among‐ and within‐individual variation from a wide array of taxa and covered a broad range of labile phenotypic traits. This study focused on the key role of phenotypic flexibility in species living in urban environments and more broadly in heterogenous environments. Urbanisation, a pervasive and spreading anthropogenic habitat alteration, has the potential to induce changes in phenotypic variation at different hierarchical levels. Although evidence was weak, our findings highlighted that—against predictions—urban individuals could have reduced phenotypic flexibility. Such changes in individual variation may have consequences on how individuals express their ecological niche through environmental tolerance. The high heterogeneity in the available dataset and the sensitivity analyses showed that the observed patterns are most likely species, taxa, and/or trait specific, supporting recent mixed results found in the literature. Changes in phenotypic variation at the within‐ or among‐individual level can have different eco‐evolutionary consequences and affect population persistence and species adaptation. Given that many changes observed in urban habitats foreshadow broader shifts driven by global change, it is essential to systematically examine how both partitions of phenotypic variation respond to such changes. Studies linking environmental heterogeneity and phenotypic variation are rare especially considering change in within‐individual variation (i.e., phenotypic flexibility). Within‐individual variation represents up to 60% of unexplained variation for many labile traits and most likely contains crucial biological information. For future research, we advocate for a more holistic framework that considers phenotypic flexibility, ideally decomposed into individual's plasticity and predictability, alongside environmental heterogeneity to better understand organismal responses to changing environments.

## Author Contributions

**Jules Petit:** conceptualisation, data curation, formal analysis, investigation, methodology, project administration, resources, software, validation, visualisation, writing – original draft, writing – review and editing. **Melanie Dammhahn:** conceptualisation, funding acquisition, project administration, resources, supervision, validation, writing – review and editing.

## Funding

This research was funded by the German Research Foundation (DFG) as part of the CRC TRR 212 (NC^3^)—Project number 316099922. The funders had no role in study design, data collection and analysis, decision to publish, or preparation of the manuscript. This work was also supported by the University of Münster.

## Conflicts of Interest

The authors declare no conflicts of interest.

## Supporting information


**Table S1:** Final search terms used per literature database.
**Figure S1:** PRISMA chart of the three screening phases and after the contact phase with criteria table.
**Table S2:** Functional definitions of the phenotypic traits incorporated in the meta‐analysis.
**Table S3:** Summary of mean and variance check for intercept‐only linear mixed effects models (IO‐LMMs) and adjusted linear mixed effects models (AD‐LMMs).
**Table S4:** Summary of estimates discarded due to impossibility of variance estimation.
**Figure S2:** Overall model estimates for lnCVR assessing differences between urban and non‐urban populations for among‐individual variation calculated from AD‐LMMs (Model 1). Positive values on the *x* axis represent higher among‐individual variation in urban populations compared to non‐urban populations whereas negative values represent the opposite phenomena. The large grey point represents the overall model estimate. The thick black line shows the 95% confidence intervals. The thin black line shows the 95% prediction intervals. Transparent small grey dots show the raw data (their size is scaled according to their sample size from which they were estimated). *k* is the number of observations supplemented with the number of studies between brackets.
**Table S5:** Meta‐analytic model estimates explaining overall variation in lnCVR of among‐individual variation (Model 1, i.e., differences in among‐individual variance between urban and non‐urban populations calculated using AD‐LMMs). CI stands for confidence intervals, PI for prediction intervals, *I* ^2^ for heterogeneity and *k* for the number of observations.
**Figure S3:** Overall model estimates for lnCVR assessing differences between urban and non‐urban populations for within‐individual variation calculated from AD‐LMMs (Model 3). Positive values on the *x* axis represent higher within‐individual variation in urban populations compared to non‐urban populations whereas negative values represent the opposite phenomena. The large grey point represents the overall model estimate. The thick black line shows the 95% confidence intervals. The thin black line shows the 95% prediction intervals. Transparent small grey dots show the raw data (their size is scaled according to their sample size from which they were estimated). *k* is the number of observations supplemented with the number of studies between brackets.
**Table S6:** Meta‐analytic model estimates explaining overall variation in lnCVR of within‐individual variation (Model 3, i.e., differences in within‐individual variance between urban and non‐urban populations calculated using AD‐LMMs). CI stands for confidence intervals, PI for prediction intervals, *I* ^2^ for heterogeneity and k for the number of observations.
**Table S7:** Meta‐analytic model estimates explaining variation in lnCVR of among‐individual variation per phenotypic trait (Model 2, i.e., differences in among‐individual variance per phenotypic trait between urban and non‐urban populations calculated using AD‐LMMs). CI stands for confidence intervals, PI for prediction intervals, *I* ^2^ for heterogeneity and k for the number of observations.
**Table S8:** Meta‐analytic model estimates explaining variation in lnCVR of within‐individual variation per phenotypic trait (Model 4, i.e., differences in within‐individual variance per phenotypic trait between urban and non‐urban populations calculated using AD‐LMMs). CI stands for confidence intervals, PI for prediction intervals, *I* ^2^ for heterogeneity and k for the number of observations.
**Table S9:** Meta‐analytic model estimates explaining variation in lnCVR of within‐individual variation per behavioural trait (Model 5, i.e., differences in within‐individual variance per behavioural trait between urban and non‐urban populations calculated using AD‐LMMs). CI stands for confidence intervals, PI for prediction intervals, *I* ^2^ for heterogeneity and k for the number of observations.
**Table S10a:** Meta‐analytic model estimates explaining variation in lnCVR of among‐individual variation due to provenance of data (Model 6a, i.e., differences in among‐individual variance between urban and non‐urban populations calculated using AD‐LMMs). CI stands for confidence intervals, PI for prediction intervals, *I* ^2^ for heterogeneity and k for the number of observations.
**Table S10b:** Meta‐analytic model estimates explaining variation in lnCVR of among‐individual variation due to interval between measurements corrected for lifespan (Model 6b, i.e., differences in among‐individual variance between urban and non‐urban populations calculated using AD‐LMMs). CI stands for confidence intervals, PI for prediction intervals, *I* ^2^ for heterogeneity and *k* for the number of observations.
**Table S10c:** Meta‐analytic model estimates explaining variation in lnCVR of among‐individual variation due to age class (Model 6c, i.e., differences in among‐individual variance between urban and non‐urban populations calculated using AD‐LMMs). CI stands for confidence intervals, PI for prediction intervals, *I* ^2^ for heterogeneity and k for the number of observations.
**Table S10d:** Meta‐analytic model estimates explaining variation in lnCVR of among‐individual variation due to taxa (Model 6d, i.e., differences in among‐individual variance between urban and non‐urban populations calculated using AD‐LMMs). CI stands for confidence intervals, PI for prediction intervals, *I* ^2^ for heterogeneity and k for the number of observations.
**Table S10e:** Meta‐analytic model estimates explaining variation in lnCVR of among‐individual variation due to number of factor included in variance partitioning models (Model 6e, i.e., differences in among‐individual variance between urban and non‐urban populations calculated using AD‐LMMs). CI stands for confidence intervals, PI for prediction intervals, *I* ^2^ for heterogeneity and *k* for the number of observations.
**Table S11:** Meta‐analytic model estimates explaining variation in lnCVR of among‐individual variation including variance differences hold by the fixed factors between urban and non‐urban populations (i.e., differences in among‐individual variance between urban and non‐urban populations calculated using AD‐LMMs). CI stands for confidence intervals, PI for prediction intervals, *I* ^2^ for heterogeneity and k for the number of observations.
**Table S12a:** Meta‐analytic model estimates explaining variation in lnCVR of within‐individual variation due to provenance of data (Model 7a, i.e., differences in within‐individual variance between urban and non‐urban populations calculated using AD‐LMMs). CI stands for confidence intervals, PI for prediction intervals, *I* ^2^ for heterogeneity and k for the number of observations.
**Table S12b:** Meta‐analytic model estimates explaining variation in lnCVR of within‐individual variation due to interval between measurements corrected for lifespan (Model 7b, i.e., differences in within‐individual variance between urban and non‐urban populations calculated using AD‐LMMs). CI stands for confidence interval, PI for prediction intervals, *I* ^2^ for heterogeneity and k for the number of observations.
**Table S12c:** Meta‐analytic model estimates explaining variation in lnCVR of within‐individual variation due to class age (Model 7c, i.e., differences in within‐individual variance between urban and non‐urban populations calculated using AD‐LMMs). CI stands for confidence interval, PI for prediction intervals, *I* ^2^ for heterogeneity and k for the number of observations.
**Table S12d:** Meta‐analytic model estimates explaining variation in lnCVR of within‐individual variation due to taxa (Model 7d, i.e., differences in within‐individual variance between urban and non‐urban populations calculated using AD‐LMMs). CI stands for confidence intervals, PI for prediction intervals, *I* ^2^ for heterogeneity and k for the number of observations.
**Table S12e:** Meta‐analytic model estimates explaining variation in lnCVR of within‐individual variation number of factor included in variance partitioning models (Model 7e, i.e., differences in within‐individual variance between urban and non‐urban populations calculated using AD‐LMMs). CI stands for confidence intervals, PI for prediction intervals, *I* ^2^ for heterogeneity and k for the number of observations.
**Table S13:** Meta‐analytic model estimates explaining variation in lnCVR of among‐individual variation comparing variance partitioning done via IOM‐LMMs with variance partitioning done via AD‐LMMs. (a) Comparison between model 8 and model 1. (b) Comparison between model 9 and model 2. CI stands for confidence intervals, PI for prediction intervals, *I* ^2^ for heterogeneity and k for the number of observations.
**Table S14:** Meta‐analytic model estimates explaining variation in lnCVR of within‐individual variation comparing variance partitioning done via IOM‐LMMs with variance partitioning done via AD‐LMMs. (a) Comparison between model 10 and model 3. (b) Comparison between model 11 and model 4. (c) Comparison between model 12 and model 5. CI stands for confidence intervals, PI for prediction intervals, *I* ^2^ for heterogeneity and k for the number of observations.
**Table S15:** Meta‐analytic model estimates explaining variation in lnCVR of (a) among‐individual and (b) within‐individual variation comparing effect size coming only from bird taxa with effect size coming only from other taxa than bird. Variance estimates from AD‐LMMs. CI stands for confidence interval, PI for prediction intervals, *I* ^2^ for heterogeneity and k for the number of observations.
**Table S16:** Single study effect model estimates of overall lnCVR of (a) among‐individual and (b) within‐individual variation. Variance estimated from AD‐LMMs. CI stands for confidence interval.
**Figure S4:** Single study effect on lnCVR estimates of among‐individual variation. The dashed red lines show the 95% confidence interval for the overall effect size in the original model. The blank dots (grey circle) show the effect sizes left out of that specific model. The thick black line shows the 95% confidence intervals. The thin black line shows the 95% prediction intervals. *k* is the number of observations supplemented with the number of studies between brackets.
**Figure S5:** Single study effect on lnCVR estimates of within‐individual variation. The dashed red lines show the 95% confidence interval for the overall effect size in the original model. The thick black line shows the 95% confidence intervals. The thin black line shows the 95% prediction intervals. The blank dots (grey circle) show the effect sizes left out of that specific model. *k* is the number of observations supplemented with the number of studies between brackets.
**Table S17:** Meta‐analytic model estimates explaining variation in lnCVR due to small‐study effect of (a) among‐individual and (b) within‐individual variation. Variance estimated from AD‐LMMs. CI stands for confidence interval, *I* ^2^ for heterogeneity and k for the number of observations, *R* ^2^ is the marginal coefficient of determination.
**Table S18:** Meta‐analytic model estimates explaining variation in lnCVR due to decline effect of (a) among‐individual and (b) within‐individual variation. Variance estimated from AD‐LMMs. CI stands for confidence interval, *I* ^2^ for heterogeneity and k for the number of observations, *R* ^2^ is the marginal coefficient of determination.
**Table S19:** PRISMA EcoEvo checklist from O'Dea et al. (2021). Summarise the main items necessary for a complete meta‐analysis. In total, we reported 73 items as 19 were non‐applicable. Overall reporting score is 97% as 2 items were not reported. As the published version might change the original format of our document, we advise to contact the main author to obtain the adequate word document.

## Data Availability

The data and code that support the findings of this study are publicly available in Dryad at https://doi.org/10.5061/dryad.2rbnzs82d.

## References

[ele70419-bib-0001] Alberti, M. 2015. “Eco‐Evolutionary Dynamics in an Urbanizing Planet.” Trends in Ecology & Evolution 30: 114–126. 25498964 10.1016/j.tree.2014.11.007

[ele70419-bib-0002] Alberti, M. , C. Correa , J. M. Marzluff , et al. 2017. “Global Urban Signatures of Phenotypic Change in Animal and Plant Populations.” Proceedings of the National Academy of Sciences 114: 8951–8956. 10.1073/pnas.1606034114 PMC5576774 28049817

[ele70419-bib-0003] Alberti, M. , E. P. Palkovacs , S. D. Roches , et al. 2020. “The Complexity of Urban Eco‐Evolutionary Dynamics.” Bioscience 70: 772–793.

[ele70419-bib-0004] Alonzo, S. H. 2015. “Integrating the How and Why of Within‐Individual and Among‐Individual Variation and Plasticity in Behavior.” Current Opinion in Behavioral Sciences, the Integrative Study of Animal Behavior 6: 69–75.

[ele70419-bib-0005] Araya‐Ajoy, Y. G. , N. J. Dingemanse , D. F. Westneat , and J. Wright . 2023. “The Evolutionary Ecology of Variation in Labile Traits: Selection on Its Among‐ and Within‐Individual Components.” Evol 77: 2246–2256. 10.1093/evolut/qpad136 37490354

[ele70419-bib-0006] Araya‐Ajoy, Y. G. , K. J. Mathot , and N. J. Dingemanse . 2015. “An Approach to Estimate Short‐Term, Long‐Term and Reaction Norm Repeatability.” Methods in Ecology and Evolution 6: 1462–1473.

[ele70419-bib-0007] Bar‐Ziv, M. , A. Sofer , A. Gorovoy , and O. Spiegel . 2022. Data for: Beyond Simple Habituation: Anthropogenic Habitats Influence the Escape Behavior of Spur‐Winged Lapwings in Response to Both Human and Non‐Human Threats [Dataset]. 10.5061/dryad.ns1rn8px7. PMC10107496 36477653

[ele70419-bib-0008] Bar‐Ziv, M. , A. Sofer , A. Gorovoy , and O. Spiegel . 2023. “Beyond Simple Habituation: Anthropogenic Habitats Influence the Escape Behaviour of Spur‐Winged Lapwings in Response to Both Human and Non‐Human Threats.” Journal of Animal Ecology 92: 417–429. 36477653 10.1111/1365-2656.13858 PMC10107496

[ele70419-bib-0009] Batabyal, A. 2017. Data for: A Multivariate Approach to Understanding Shifts in Escape Strategies of Urban Lizards [Dataset]. 10.5061/dryad.2rbnzs82d.

[ele70419-bib-0010] Batabyal, A. , S. Balakrishna , and M. Thaker . 2017. “A Multivariate Approach to Understanding Shifts in Escape Strategies of Urban Lizards.” Behavioral Ecology and Sociobiology 71: 83.

[ele70419-bib-0160] Batabyal, A. , and M. Thaker . 2019a. Data for: Lizards from Suburban Areas Learn Faster to Stay Safe [Dataset]. 10.1098/rsbl.2019.0009. PMC6405467 30958137

[ele70419-bib-0011] Batabyal, A. , and M. Thaker . 2019b. “Lizards From Suburban Areas Learn Faster to Stay Safe.” Biology Letters 15: 20190009. 10.1098/rsbl.2019.0009. 30958137 PMC6405467

[ele70419-bib-0012] Batáry, P. , K. Kurucz , M. Suarez‐Rubio , and D. E. Chamberlain . 2018. “Non‐Linearities in Bird Responses Across Urbanization Gradients: A Meta‐Analysis.” Global Change Biology 24: 1046–1054. 29080260 10.1111/gcb.13964

[ele70419-bib-0013] Bates, D. , M. Mächler , B. Bolker , and S. Walker . 2015. “Fitting Linear Mixed‐Effects Models Using lme4.” Journal of Statistical Software 67: 1–48.

[ele70419-bib-0014] Biondi, L. M. 2021. Data for: Behavioural Factors Underlying Innovative Problem‐Solving Differences in an Avian Predator From Two Contrasting Habitats [Dataset]. 10.5061/dryad.2rbnzs82d. 34709499

[ele70419-bib-0015] Biondi, L. M. , G. Fuentes , and M. Susana . 2022. “Behavioural Factors Underlying Innovative Problem‐Solving Differences in an Avian Predator From Two Contrasting Habitats.” Animal Cognition 25: 529–543. 34709499 10.1007/s10071-021-01569-2

[ele70419-bib-0016] Blumstein, D. T. 2016. “Habituation and Sensitization: New Thoughts About Old Ideas.” Animal Behaviour 120: 255–262.

[ele70419-bib-0017] Bolnick, D. I. , P. Amarasekare , M. S. Araújo , et al. 2011. “Why Intraspecific Trait Variation Matters in Community Ecology.” Trends in Ecology & Evolution 26: 183–192. 21367482 10.1016/j.tree.2011.01.009 PMC3088364

[ele70419-bib-0018] Brans, K. I. , and L. De Meester . 2018. “City Life on Fast Lanes: Urbanization Induces an Evolutionary Shift Towards a Faster Lifestyle in the Water Flea Daphnia.” Functional Ecology 32: 2225–2240.

[ele70419-bib-0019] Brans, K. I. , L. Govaert , J. M. T. Engelen , A. T. Gianuca , C. Souffreau , and L. De Meester . 2017. “Eco‐Evolutionary Dynamics in Urbanized Landscapes: Evolution, Species Sorting and the Change in Zooplankton Body Size Along Urbanization Gradients.” Philosophical Transactions of the Royal Society of London. Series B, Biological Sciences 372: 20160030. 27920375 10.1098/rstb.2016.0030 PMC5182426

[ele70419-bib-0020] Brans, K. I. , R. Stoks , and L. De Meester . 2018. “Urbanization Drives Genetic Differentiation in Physiology and Structures the Evolution of Pace‐of‐Life Syndromes in the Water Flea *Daphnia magna* .” Proceedings of the Biological Sciences 285: 20180169. 30051844 10.1098/rspb.2018.0169 PMC6083254

[ele70419-bib-0021] Brommer, J. E. 2013. “Phenotypic Plasticity of Labile Traits in the Wild.” Current Zoology 59: 485–505.

[ele70419-bib-0022] Bryk, A. S. , and S. W. Raudenbush . 1992. Hierarchical Linear Models: Applications and Data Analysis Methods. Hierarchical linear models: Applications and data analysis methods. Sage Publications, Inc.

[ele70419-bib-0023] Burkhard, T. T. , N. A. Dochtermann , and A. Charmantier . 2026. “Global Meta‐Analysis Reveals Urban‐Associated Behavioural Differences Among Wild Populations.” Journal of Animal Ecology: 1–19. https://doi/10.1111/1365‐2656.70269. 10.1111/1365-2656.70269 PMC13536287 42152172

[ele70419-bib-0024] Cadenasso, M. L. , S. T. A. Pickett , and K. Schwarz . 2007. “Spatial Heterogeneity in Urban Ecosystems: Reconceptualizing Land Cover and a Framework for Classification.” Frontiers in Ecology and the Environment 5: 80–88.

[ele70419-bib-0025] Callaghan, C. T. , R. E. Major , J. H. Wilshire , J. M. Martin , R. T. Kingsford , and W. K. Cornwell . 2019. “Generalists Are the Most Urban‐Tolerant of Birds: A Phylogenetically Controlled Analysis of Ecological and Life History Traits Using a Novel Continuous Measure of Bird Responses to Urbanization.” Oikos 128: 845–858.

[ele70419-bib-0026] Capilla‐Lasheras, P. , M. J. Thompson , A. Sánchez‐Tójar , et al. 2022a. “A Global Meta‐Analysis Reveals Higher Variation in Breeding Phenology in Urban Birds Than in Their Non‐Urban Neighbours.” Ecology Letters 25: 2552–2570. 36136999 10.1111/ele.14099 PMC9826320

[ele70419-bib-0027] Capilla‐Lasheras, P. , M. J. Thompson , A. Sánchez‐Tójar , et al. 2022b. A Global Meta‐Analysis Reveals Higher Variation in Breeding Phenology in Urban Birds Than in Their Non‐Urban Neighbours [Code]. 10.5281/zenodo.7010687. PMC9826320 36136999

[ele70419-bib-0028] Čapkun‐Huot, C. , D. T. Blumstein , D. Garant , D. Sol , and D. Réale . 2024. “Toward a Unified Framework for Studying Behavioural Tolerance.” Trends in Ecology & Evolution 39: 446–455. 38177010 10.1016/j.tree.2023.12.006

[ele70419-bib-0029] Cinar, O. , S. Nakagawa , and W. Viechtbauer . 2022. “Phylogenetic Multilevel Meta‐Analysis: A Simulation Study on the Importance of Modelling the Phylogeny.” Methods in Ecology and Evolution 13: 383–395.

[ele70419-bib-0030] Cleasby, I. R. , S. Nakagawa , and H. Schielzeth . 2015. “Quantifying the Predictability of Behaviour: Statistical Approaches for the Study of Between‐Individual Variation in the Within‐Individual Variance.” Methods in Ecology and Evolution 6: 27–37.

[ele70419-bib-0031] Cohen, J. E. , and M. Xu . 2015. “Random Sampling of Skewed Distributions Implies Taylor's Power Law of Fluctuation Scaling.” Proceedings of the National Academy of Sciences 112: 7749–7754. 10.1073/pnas.1503824112 PMC4485080 25852144

[ele70419-bib-0032] Dammhahn, M. , V. Mazza , A. Schirmer , C. Göttsche , and J. A. Eccard . 2020. “Of City and Village Mice: Behavioural Adjustments of Striped Field Mice to Urban Environments.” Scientific Reports 10: 13056. 32747632 10.1038/s41598-020-69998-6 PMC7400609

[ele70419-bib-0033] Darwin, C. 1859. On the Origin of Species by Means of Natural Selection, or the Preservation of Favoured Races in the Struggle for Life. Murray. PMC5184128 30164232

[ele70419-bib-0034] De Villemereuil, P. , M. B. Morrissey , S. Nakagawa , and H. Schielzeth . 2018. “Fixed‐Effect Variance and the Estimation of Repeatabilities and Heritabilities: Issues and Solutions.” Journal of Evolutionary Biology 31: 621–632. 29285829 10.1111/jeb.13232

[ele70419-bib-0035] Delaney, K. S. 2013. “Landscape Genetics of Urban Bird Populations.” In Avian Urban Ecology, edited by D. Gil and H. Brumm . Oxford University Press.

[ele70419-bib-0036] Devictor, V. , R. Julliard , D. Couvet , A. Lee , and F. Jiguet . 2007. “Functional Homogenization Effect of Urbanization on Bird Communities.” Conservation Biology 21: 741–751. 17531052 10.1111/j.1523-1739.2007.00671.x

[ele70419-bib-0037] DeWitt, T. J. , A. Sih , and D. S. Wilson . 1998. “Costs and Limits of Phenotypic Plasticity.” Trends in Ecology & Evolution 13: 77–81. 21238209 10.1016/s0169-5347(97)01274-3

[ele70419-bib-0038] Dingemanse, N. J. , and N. A. Dochtermann . 2013. “Quantifying Individual Variation in Behaviour: Mixed‐Effect Modelling Approaches.” Journal of Animal Ecology 82: 39–54. 23171297 10.1111/1365-2656.12013

[ele70419-bib-0039] Dingemanse, N. J. , A. J. N. Kazem , D. Réale , and J. Wright . 2010. “Behavioural Reaction Norms: Animal Personality Meets Individual Plasticity.” Trends in Ecology & Evolution 25: 81–89. 19748700 10.1016/j.tree.2009.07.013

[ele70419-bib-0040] Dingemanse, N. J. , and D. Réale . 2005. “Natural Selection and Animal Personality.” Behaviour 142: 1159–1184.

[ele70419-bib-0041] Dingemanse, N. J. , and M. Wolf . 2013. “Between‐Individual Differences in Behavioural Plasticity Within Populations: Causes and Consequences.” Animal Behaviour 85: 1031–1039.

[ele70419-bib-0042] Dominoni, D. , J. A. H. Smit , M. E. Visser , and W. Halfwerk . 2020. “Multisensory Pollution: Artificial Light at Night and Anthropogenic Noise Have Interactive Effects on Activity Patterns of Great Tits ( *Parus major* ).” Environmental Pollution 256: 113314. 31761596 10.1016/j.envpol.2019.113314

[ele70419-bib-0043] Dominoni, D. M. 2015. Data for: Social Cues Are Unlikely to be the Single Cause for Early Reproduction in Urban European Blackbirds ( *Turdus merula* ) [Dataset]. 10.5061/dryad.2rbnzs82d. 25619949

[ele70419-bib-0044] Dominoni, D. M. 2020. Data for: Multisensory Pollution: Artificial Light at Night and Anthropogenic Noise Have Interactive Effects on Activity Patterns of Great Tits ( *Parus major* ) [Dataset]. 10.5061/dryad.2rbnzs82d. 31761596

[ele70419-bib-0045] Dominoni, D. M. , T. J. Van’t Hof , and J. Partecke . 2015. “Social Cues Are Unlikely to be the Single Cause for Early Reproduction in Urban European Blackbirds ( *Turdus merula* ).” Physiology & Behavior 142: 14–19. 25619949 10.1016/j.physbeh.2015.01.026

[ele70419-bib-0046] Edelaar, P. , and D. I. Bolnick . 2019. “Appreciating the Multiple Processes Increasing Individual or Population Fitness.” Trends in Ecology & Evolution 34: 435–446. 30850175 10.1016/j.tree.2019.02.001

[ele70419-bib-0047] Emerson, J. D. 1991. “Introduction to Transformation.” In Fundamentals of Exploratory Analysis of Variance, 365–400. John Wiley & Sons, Ltd.

[ele70419-bib-0048] Falconer, D. S. , and T. F. C. Mackay . 1996. Introduction to Quantitative Genetics. 4th ed. Pearson. 10.1093/genetics/167.4.1529 PMC1471025 15342495

[ele70419-bib-0049] Forsman, A. , and L. Wennersten . 2016. “Inter‐Individual Variation Promotes Ecological Success of Populations and Species: Evidence From Experimental and Comparative Studies.” Ecography 39: 630–648.

[ele70419-bib-0050] Fox, R. J. , J. M. Donelson , C. Schunter , T. Ravasi , and J. D. Gaitán‐Espitia . 2019. “Beyond Buying Time: The Role of Plasticity in Phenotypic Adaptation to Rapid Environmental Change.” Philosophical Transactions of the Royal Society, B: Biological Sciences 374: 20180174. 10.1098/rstb.2018.0174 PMC6365870 30966962

[ele70419-bib-0051] Garitano‐Zavala, Á. 2022. Data for: The Behavioral Responses of the Chiguanco Thrush to Urbanization in a Neotropical City Comes From Preadapted Behavioral Traits [Dataset]. 10.5061/dryad.2rbnzs82d.

[ele70419-bib-0052] Garitano‐Zavala, Á. , R. Calbimonte , and G. Esteve‐Herraiz . 2022. “The Behavioral Responses of the Chiguanco Thrush to Urbanization in a Neotropical City Comes From Preadapted Behavioral Traits.” Frontiers in Ecology and Evolution: 10.

[ele70419-bib-0053] Gervais, L. , M. J. Thompson , P. de Villemereuil , et al. 2025. “Behavioral DiverCity: Individual Differences in Behavior Change Along an Urbanization Gradient.” Behavioral Ecology 36: araf035. 40575390 10.1093/beheco/araf035 PMC12202043

[ele70419-bib-0054] Grafen, A. 1989. “The Phylogenetic Regression.” Phil. Trans. R. Soc. Lond. B 326: 119–157. 2575770 10.1098/rstb.1989.0106

[ele70419-bib-0055] Grimm, N. B. , S. H. Faeth , N. E. Golubiewski , et al. 2008. “Global Change and the Ecology of Cities.” Science 319: 756–760. 18258902 10.1126/science.1150195

[ele70419-bib-0056] Grunst, M. L. , J. T. Rotenberry , and A. S. Grunst . 2014. “Variation in Adrenocortical Stress Physiology and Condition Metrics Within a Heterogeneous Urban Environment in the Song Sparrow *Melospiza melodia* .” Journal of Avian Biology 45: 574–583.

[ele70419-bib-0057] Hardman, S. I. , and S. Dalesman . 2018a. “Data for: Repeatability and Degree of Territorial Aggression Differs Among Urban and Rural Great Tits (*Parus major*) [Datatset].” Scientific Reports. 10.1038/s41598-018-23463-7. PMC5864914 29568056

[ele70419-bib-0159] Hardman, S. I. , and S. Dalesman . 2018b. “Repeatability and Degree of Territorial Aggression Differs Among Urban and Rural Great Tits (*Parus major*).” Scientific Reports 8: 5042. 29568056 10.1038/s41598-018-23463-7 PMC5864914

[ele70419-bib-0058] Harten, L. , N. Gonceer , M. Handel , O. Dash , H. B. Fokidis , and Y. Yovel . 2021. “Urban Bat Pups Take After Their Mothers and Are Bolder and Faster Learners Than Rural Pups.” BMC Biology 19: 190. 34493290 10.1186/s12915-021-01131-z PMC8422611

[ele70419-bib-0059] Hendry, A. P. , T. J. Farrugia , and M. T. Kinnison . 2008. “Human Influences on Rates of Phenotypic Change in Wild Animal Populations.” Molecular Ecology 17: 20–29. 18173498 10.1111/j.1365-294X.2007.03428.x

[ele70419-bib-0060] Heppner, J. J. 2023. Data for: Urbanization and Maternal Hormone Transfer: Endocrine and Morphological Phenotypes Across Ontogenetic Stages [Dataset]. 10.5061/dryad.2rbnzs82d. PMC10978011 36402244

[ele70419-bib-0061] Heppner, J. J. , J. S. Krause , and J. Q. Ouyang . 2023. “Urbanization and Maternal Hormone Transfer: Endocrine and Morphological Phenotypes Across Ontogenetic Stages.” General and Comparative Endocrinology 333: 114166. 36402244 10.1016/j.ygcen.2022.114166 PMC10978011

[ele70419-bib-0062] Hertel, A. G. , P. T. Niemelä , N. J. Dingemanse , and T. Mueller . 2020. “A Guide for Studying Among‐Individual Behavioral Variation From Movement Data in the Wild.” Movement Ecology 8: 30. 32612837 10.1186/s40462-020-00216-8 PMC7325061

[ele70419-bib-0063] Higgins, J. P. T. , S. G. Thompson , J. J. Deeks , and D. G. Altman . 2003. “Measuring Inconsistency in Meta‐Analyses.” BMJ 327: 557–560. 12958120 10.1136/bmj.327.7414.557 PMC192859

[ele70419-bib-0064] Hinchliff, C. E. , S. A. Smith , J. F. Allman , et al. 2015. “Synthesis of Phylogeny and Taxonomy Into a Comprehensive Tree of Life.” Proceedings of the National Academy of Sciences 112: 12764–12769. 10.1073/pnas.1423041112 PMC4611642 26385966

[ele70419-bib-0065] Huang, P. 2020. Data for: Habitat Urbanization and Stress Response Are Primary Predictors of Personality Variation in Northern Cardinals (*Cardinalis cardinalis*) [Dataset]. 10.5061/dryad.2rbnzs82d.

[ele70419-bib-0066] Huang, P. , C. M. St. Mary , and R. T. Kimball . 2020. “Habitat Urbanization and Stress Response Are Primary Predictors of Personality Variation in Northern Cardinals ( *Cardinalis cardinalis* ).” Journal of Urban Ecology 6: juaa015.

[ele70419-bib-0067] Iglesias‐Carrasco, M. , U. Aich , M. D. Jennions , and M. L. Head . 2020. “Stress in the City: Meta‐Analysis Indicates no Overall Evidence for Stress in Urban Vertebrates.” Proceedings of the Royal Society B: Biological Sciences 287: 20201754. 10.1098/rspb.2020.1754 PMC7657868 33023414

[ele70419-bib-0068] Jakubas, D. , P. Indykiewicz , J. Kowalski , T. Iciek , and P. Minias . 2020a. “Data for: Inter‐Colony Variation in Foraging Flight Characteristics of Black‐Headed Gulls *Chroicocephalus ridibundus* During the Incubating Period [Dataset].” Ecology and Evolution. 10.5061/dryad.q83bk3jfg. PMC7319234 32607169

[ele70419-bib-0069] Jakubas, D. , P. Indykiewicz , J. Kowalski , T. Iciek , and P. Minias . 2020b. “Intercolony Variation in Foraging Flight Characteristics of Black‐Headed Gulls *Chroicocephalus ridibundus* During the Incubation Period.” Ecology and Evolution 10: 5489–5505. 32607169 10.1002/ece3.6291 PMC7319234

[ele70419-bib-0070] Kaiser, A. 2018. Data for: Urbanisation and Sex Affect the Consistency of Butterfly Personality Across Metamorphosis [Dataset]. 10.5061/dryad.2rbnzs82d.

[ele70419-bib-0071] Kaiser, A. 2019. Data for: Behavioural Repeatability is Affected by Early Developmental Conditions in a Butterfly [Dataset]. 10.5061/dryad.2rbnzs82d.

[ele70419-bib-0072] Kaiser, A. 2020. Data for: An Experimental Test of Changed Personality in Butterflies From Anthropogenic Landscapes [Dataset]. 10.5061/dryad.2rbnzs82d.

[ele70419-bib-0073] Kaiser, A. , T. Merckx , and H. Van Dyck . 2018. “Urbanisation and Sex Affect the Consistency of Butterfly Personality Across Metamorphosis.” Behavioral Ecology and Sociobiology 72: 1–11.

[ele70419-bib-0074] Kaiser, A. , T. Merckx , and H. Van Dyck . 2019. “Behavioural Repeatability Is Affected by Early Developmental Conditions in a Butterfly.” Animal Behaviour 157: 219–226.

[ele70419-bib-0075] Kaiser, A. , T. Merckx , and H. Van Dyck . 2020. “An Experimental Test of Changed Personality in Butterflies From Anthropogenic Landscapes.” Behavioral Ecology and Sociobiology 74: 86.

[ele70419-bib-0076] Kelley, J. L. , L. Chapuis , W. I. L. Davies , and S. P. Collin . 2018. “Sensory System Responses to Human‐Induced Environmental Change.” Frontiers in Ecology and Evolution 6.

[ele70419-bib-0077] Kozlovsky, D. Y. , E. A. Weissgerber , and V. V. Pravosudov . 2017a. Data for: What Makes Specialized Food‐Caching Mountain Chickadees Successful City Slickers? [Dataset]. 10.6084/m9.figshare.4982693. PMC5454252 28539508

[ele70419-bib-0078] Kozlovsky, D. Y. , E. A. Weissgerber , and V. V. Pravosudov . 2017e. “What Makes Specialized Food‐Caching Mountain Chickadees Successful City Slickers?” Proceedings of the Royal Society B: Biological Sciences 284: 20162613. 10.1098/rspb.2016.2613 PMC5454252 28539508

[ele70419-bib-0079] Lynch, M. , and B. Walsh . 1998. Genetics and Analysis of Quantitative Traits. Sinauer Associates (an imprint of Oxford University Press).

[ele70419-bib-0080] March‐Salas, M. , G. Fandos , and P. S. Fitze . 2021. “Effects of Intrinsic Environmental Predictability on Intra‐Individual and Intra‐Population Variability of Plant Reproductive Traits and Eco‐Evolutionary Consequences.” Annals of Botany 127: 413–423. 32421780 10.1093/aob/mcaa096 PMC7988524

[ele70419-bib-0081] Martin, J. G. A. , E. Pirotta , M. B. Petelle , and D. T. Blumstein . 2017. “Genetic Basis of Between‐Individual and Within‐Individual Variance of Docility.” Journal of Evolutionary Biology 30: 796–805. 28182325 10.1111/jeb.13048

[ele70419-bib-0082] Matesanz, S. , E. Gianoli , and F. Valladares . 2010. “Global Change and the Evolution of Phenotypic Plasticity in Plants.” Annals of the New York Academy of Sciences 1206: 35–55. 20860682 10.1111/j.1749-6632.2010.05704.x

[ele70419-bib-0083] Mazza, V. 2020. Data for: Small Mammals in the Big City: Behavioural Adjustments of Non‐Commensal Rodents to Urban Environments [Dataset]. 10.5061/dryad.2rbnzs82d. 32767603

[ele70419-bib-0084] Mazza, V. , M. Dammhahn , E. Lösche , and J. A. Eccard . 2020. “Small Mammals in the Big City: Behavioural Adjustments of Non‐Commensal Rodents to Urban Environments.” Global Change Biology 26: 6326–6337. 32767603 10.1111/gcb.15304

[ele70419-bib-0085] Mazza, V. , and A. Guenther . 2021. “City Mice and Country Mice: Innovative Problem Solving in Rural and Urban Noncommensal Rodents.” Animal Behaviour 172: 197–210.

[ele70419-bib-0086] Medina, I. , G. M. Cooke , and T. J. Ord . 2018. “Walk, Swim or Fly? Locomotor Mode Predicts Genetic Differentiation in Vertebrates.” Ecology Letters 21: 638–645. 29527800 10.1111/ele.12930

[ele70419-bib-0087] Michonneau, F. , J. W. Brown , and D. J. Winter . 2016. “Rotl: An R Package to Interact With the Open Tree of Life Data.” Methods in Ecology and Evolution 7: 1476–1481.

[ele70419-bib-0088] Miles, L. S. , L. R. Rivkin , M. T. J. Johnson , J. Munshi‐South , and B. C. Verrelli . 2019. “Gene Flow and Genetic Drift in Urban Environments.” Molecular Ecology 28: 4138–4151. 31482608 10.1111/mec.15221

[ele70419-bib-0089] Nakagawa, S. , M. Lagisz , M. D. Jennions , et al. 2022. “Methods for Testing Publication Bias in Ecological and Evolutionary Meta‐Analyses.” Methods in Ecology and Evolution 13: 4–21.

[ele70419-bib-0090] Nakagawa, S. , M. Lagisz , R. E. O’Dea , et al. 2021. “The Orchard Plot: Cultivating a Forest Plot for Use in Ecology, Evolution, and Beyond.” Research Synthesis Methods 12: 4–12. 32445243 10.1002/jrsm.1424

[ele70419-bib-0091] Nakagawa, S. , R. Poulin , K. Mengersen , et al. 2015. “Meta‐Analysis of Variation: Ecological and Evolutionary Applications and Beyond.” Methods in Ecology and Evolution 6: 143–152.

[ele70419-bib-0092] Nakagawa, S. , and E. S. A. Santos . 2012. “Methodological Issues and Advances in Biological Meta‐Analysis.” Evolutionary Ecology 26: 1253–1274.

[ele70419-bib-0093] Nakagawa, S. , and H. Schielzeth . 2010. “Repeatability for Gaussian and Non‐Gaussian Data: A Practical Guide for Biologists.” Biological Reviews 85: 935–956. 20569253 10.1111/j.1469-185X.2010.00141.x

[ele70419-bib-0094] Nicotra, A. B. , O. K. Atkin , S. P. Bonser , et al. 2010. “Plant Phenotypic Plasticity in a Changing Climate.” Trends in Plant Science 15: 684–692. 20970368 10.1016/j.tplants.2010.09.008

[ele70419-bib-0095] Nonacs, P. , P. J. Yeh , D. T. Blumstein , and H. M. Stansell . 2021. Data for: Individual Variation in Tolerance of Human Activity by Urban Dark‐Eyed Juncos (*Junco hyemalis*) [Dataset]. 10.5068/D1MT2P.

[ele70419-bib-0096] O’Donnell, K. , and J. delBarco‐Trillo . 2020. “Changes in the Home Range Sizes of Terrestrial Vertebrates in Response to Urban Disturbance: A Meta‐Analysis.” Journal of Urban Ecology 6: juaa014.

[ele70419-bib-0097] OpenTreeOfLife , B. Redelings , L. L. S. Reyes , K. A. Cranston , et al. 2019. Open Tree of Life Synthetic Tree.

[ele70419-bib-0098] Ouyang, J. Q. , D. Baldan , C. Munguia , and S. Davies . 2019a. Data for: Genetic Inheritance and Environment Determine Endocrine Plasticity to Urban Living [Dataset]. 10.5061/dryad.5p3q298. PMC6710584 31362633

[ele70419-bib-0099] Ouyang, J. Q. , D. Baldan , C. Munguia , and S. Davies . 2019b. “Genetic Inheritance and Environment Determine Endocrine Plasticity to Urban Living.” Proceedings of the Royal Society B: Biological Sciences 286: 20191215. 10.1098/rspb.2019.1215 PMC6710584 31362633

[ele70419-bib-0100] Ouzzani, M. , H. Hammady , Z. Fedorowicz , and A. Elmagarmid . 2016. “Rayyan—A Web and Mobile App for Systematic Reviews.” Systematic Reviews 5: 210. 27919275 10.1186/s13643-016-0384-4 PMC5139140

[ele70419-bib-0101] Papp, S. , E. Vincze , B. Preiszner , A. Liker , and V. Bókony . 2015. “A Comparison of Problem‐Solving Success Between Urban and Rural House Sparrows.” Behavioral Ecology and Sociobiology 69: 471–480.

[ele70419-bib-0102] Petit, J. , and M. Dammhahn . 2024. Data for: Petit & Dammhahn Dataset. 10.5061/dryad.2rbnzs82d.

[ele70419-bib-0103] Piano, E. , K. De Wolf , F. Bona , et al. 2017. “Urbanization Drives Community Shifts Towards Thermophilic and Dispersive Species at Local and Landscape Scales.” Global Change Biology 23: 2554–2564. 27997069 10.1111/gcb.13606

[ele70419-bib-0104] Pickett, S. T. A. , M. L. Cadenasso , D. L. Childers , M. J. McDonnell , and W. Zhou . 2016. “Evolution and Future of Urban Ecological Science: Ecology in, of, and for the City.” Ecosystem Health and Sustainability 2: e01229.

[ele70419-bib-0105] Pickett, S. T. A. , M. L. Cadenasso , M. J. Grove , C. H. Nilon , R. V. Pouyat , and W. C. Zipperer . 2001. “Urban Ecological Systems: Linking Terrestrial Ecological, Physical, and Socioeconomic Components of Metropolitan Areas.” Annual Review of Ecological Systems 32: 127–157.

[ele70419-bib-0106] Piersma, T. , and J. Drent . 2003. “Phenotypic Flexibility and the Evolution of Organismal Design.” Trends in Ecology & Evolution 18: 228–233.

[ele70419-bib-0107] Pigliucci, M. , C. D. Schlichting , C. S. Jones , and K. Schwenk . 1996. “Developmental Reaction Norms: The Interactions Among Allometry, Ontogeny and Plasticity.” Plant Species Biology 11: 69–85.

[ele70419-bib-0108] Prasher, S. , J. C. Evans , M. J. Thompson , and J. Morand‐Ferron . 2019a. “Characterizing Innovators: Ecological and Individual Predictors of Problem‐Solving Performance.” PLoS One 14: e0217464. 31188843 10.1371/journal.pone.0217464 PMC6561637

[ele70419-bib-0109] Prasher, S. , J. C. Evans , M. J. Thompson , and J. Morand‐Ferron . 2019b. Data for: Characterizing Innovators: Ecological and Individual Predictors of Problem‐Solving Performance [Dataset]. 10.5061/dryad.s83d4n1. PMC6561637 31188843

[ele70419-bib-0110] Putman, B. J. , and Z. A. Tippie . 2020. “Big City Living: A Global Meta‐Analysis Reveals Positive Impact of Urbanization on Body Size in Lizards.” Frontiers in Ecology and Evolution 8.

[ele70419-bib-0111] R Core Team . 2022. R: A Language and Environment for Statistical Computing. R Foundation for Statistical Computing.

[ele70419-bib-0112] Réale, D. , S. M. Reader , D. Sol , P. T. McDougall , and N. J. Dingemanse . 2007. “Integrating Animal Temperament Within Ecology and Evolution.” Biological Reviews 82: 291–318. 17437562 10.1111/j.1469-185X.2007.00010.x

[ele70419-bib-0113] Rees, J. A. , and K. Cranston . 2017. “Automated Assembly of a Reference Taxonomy for Phylogenetic Data Synthesis.” Biodiversity Data Journal 5: e12581. 10.3897/BDJ.5.e12581 PMC5515096 28765728

[ele70419-bib-0114] Rimbach, R. , and M. Dammhahn . 2024. Data for: Rimbach & Dammhahn Dataset [Dataset]. 10.5061/dryad.2rbnzs82d.

[ele70419-bib-0115] Rimbach, R. , K. Heinze , L. Poorthuis , J. Petit , and M. Dammhahn . 2025. “City Life Does Not Change a Small Mammal Community Composition.” Wildlife Research 52.

[ele70419-bib-0116] Rivkin, L. R. , J. S. Santangelo , M. Alberti , et al. 2019. “A Roadmap for Urban Evolutionary Ecology.” Evolutionary Applications 12: 384–398. 30828362 10.1111/eva.12734 PMC6383741

[ele70419-bib-0117] Searle, S. R. , G. Casella , and C. E. McCulloch . 1992. Variance Components. 1st ed. Wiley Series in Probability and Statistics.

[ele70419-bib-0118] Sears, K. E. 2014. “Quantifying the Impact of Development on Phenotypic Variation and Evolution.” Journal of Experimental Zoology Part B: Molecular and Developmental Evolution 322: 643–653. 25393554 10.1002/jez.b.22592

[ele70419-bib-0119] Senior, A. M. , C. E. Grueber , T. Kamiya , et al. 2016. “Heterogeneity in Ecological and Evolutionary Meta‐Analyses: Its Magnitude and Implications.” Ecology 97: 3293–3299. 27912008 10.1002/ecy.1591

[ele70419-bib-0120] Senior, A. M. , W. Viechtbauer , and S. Nakagawa . 2020. “Revisiting and Expanding the Meta‐Analysis of Variation: The Log Coefficient of Variation Ratio.” Research Synthesis Methods 11: 553–567. 32431099 10.1002/jrsm.1423

[ele70419-bib-0121] Sih, A. , M. C. O. Ferrari , and D. J. Harris . 2011. “Evolution and Behavioural Responses to Human‐Induced Rapid Environmental Change.” Evolutionary Applications 4: 367–387. 25567979 10.1111/j.1752-4571.2010.00166.x PMC3352552

[ele70419-bib-0122] Silva, C. P. , R. D. Sepúlveda , and O. Barbosa . 2016. “Nonrandom Filtering Effect on Birds: Species and Guilds Response to Urbanization.” Ecology and Evolution 6: 3711–3720. 27231534 10.1002/ece3.2144 PMC4864331

[ele70419-bib-0123] Smit, J. A. H. , R. Vooijs , P. Lindenburg , A. T. Baugh , and W. Halfwerk . 2023. Data for: Noise and Light Pollution Elicit Endocrine Responses in Urban but Not Forest Frogs [Dataset]. 10.34894/TA0XUT. 37979210

[ele70419-bib-0124] Smit, J. A. H. , R. Vooijs , P. Lindenburg , A. T. Baugh , and W. Halfwerk . 2024. “Noise and Light Pollution Elicit Endocrine Responses in Urban but Not Forest Frogs.” Hormones and Behavior 157: 105453. 37979210 10.1016/j.yhbeh.2023.105453

[ele70419-bib-0125] Snell‐Rood, E. C. 2013. “An Overview of the Evolutionary Causes and Consequences of Behavioural Plasticity.” Animal Behaviour 85: 1004–1011.

[ele70419-bib-0126] Solaro, C. , and J. H. Sarasola . 2019. “Urban Living Predicts Behavioural Response in a Neotropical Raptor.” Behavioural Processes 169: 103995. 31698033 10.1016/j.beproc.2019.103995

[ele70419-bib-0127] Soltani, A. , and E. Sharifi . 2017. “Daily Variation of Urban Heat Island Effect and Its Correlations to Urban Greenery: A Case Study of Adelaide.” Frontiers of Architectural Research 6: 529–538.

[ele70419-bib-0128] Sorace, A. , and M. Gustin . 2009. “Distribution of Generalist and Specialist Predators Along Urban Gradients.” Landscape and Urban Planning 90: 111–118.

[ele70419-bib-0129] Sprau, P. , and N. J. Dingemanse . 2017. “An Approach to Distinguish Between Plasticity and Non‐Random Distributions of Behavioral Types Along Urban Gradients in a Wild Passerine Bird.” Frontiers in Ecology and Evolution 5.

[ele70419-bib-0130] Stansell, H. M. , D. T. Blumstein , P. J. Yeh , and P. Nonacs . 2022. “Individual Variation in Tolerance of Human Activity by Urban Dark‐Eyed Juncos ( *Junco hyemalis* ).” Wilson Journal of Ornithology 134: 43–51.

[ele70419-bib-0131] Stofberg, M. , S. J. Cunningham , P. Sumasgutner , and A. Amar . 2019. “Juggling a “Junk‐Food” Diet: Responses of an Urban Bird to Fluctuating Anthropogenic‐Food Availability.” Urban Ecosystem 22: 1019–1026.

[ele70419-bib-0132] Svanbäck, R. , and D. I. Bolnick . 2005. “Intraspecific Competition Affects the Strength of Individual Specialization: An Optimal Diet Theory Method.” Evolutionary Ecology Research 7: 993–1012.

[ele70419-bib-0133] Svanbäck, R. , and D. I. Bolnick . 2006. “Intraspecific Competition Drives Increased Resource Use Diversity Within a Natural Population.” Proceedings of the Royal Society B: Biological Sciences 274: 839–844. 10.1098/rspb.2006.0198 PMC2093969 17251094

[ele70419-bib-0134] Szabó, B. , D. Korányi , R. Gallé , G. L. Lövei , G. Bakonyi , and P. Batáry . 2023. “Urbanization Decreases Species Richness, and Increases Abundance in Dry Climates Whereas Decreases in Wet Climates: A Global Meta‐Analysis.” Science of the Total Environment 859: 160145. 36395843 10.1016/j.scitotenv.2022.160145

[ele70419-bib-0135] Szulkin, M. , C. J. Garroway , M. Corsini , A. Z. Kotarba , and D. Dominoni . 2020. “How to Quantify Urbanization When Testing for Urban Evolution?” In Urban Evolutionary Biology, edited by M. Szulkin , J. Munshi‐South , and A. Charmantier . Oxford University Press.

[ele70419-bib-0136] Tabh, J. K. R. , G. F. Mastromonaco , and G. Burness . 2020. Data for: Stress‐Induced Changes in Body Surface Temperature Are Repeatable, but do Not Differ Between Urban and Rural Birds [Datatset]. 10.5061/dryad.2rbnzs82d. 35138449

[ele70419-bib-0137] Tabh, J. K. R. , G. F. Mastromonaco , and G. Burness . 2022. “Stress‐Induced Changes in Body Surface Temperature Are Repeatable, but Do Not Differ Between Urban and Rural Birds.” Oecologia 198: 663–677. 35138449 10.1007/s00442-022-05120-z

[ele70419-bib-0138] Tam, B. Y. , W. A. Gough , and T. Mohsin . 2015. “The Impact of Urbanization and the Urban Heat Island Effect on Day to Day Temperature Variation.” Urban Climate 12: 1–10.

[ele70419-bib-0139] Thompson, M. J. , P. Capilla‐Lasheras , D. M. Dominoni , D. Réale , and A. Charmantier . 2022. “Phenotypic Variation in Urban Environments: Mechanisms and Implications.” Trends in Ecology & Evolution 37: 171–182. 34690006 10.1016/j.tree.2021.09.009

[ele70419-bib-0140] Thompson, M. J. , J. C. Evans , S. Parsons , and J. Morand‐Ferron . 2018a. Data for: Urbanization and Individual Differences in Exploration and Plasticity [Dataset]. 10.5061/dryad.kv7qp23.

[ele70419-bib-0141] Thompson, M. J. , J. C. Evans , S. Parsons , and J. Morand‐Ferron . 2018b. “Urbanization and Individual Differences in Exploration and Plasticity.” Behavioral Ecology 29, no. 6: 1415–1425.

[ele70419-bib-0142] Thompson, M. J. , J. G. A. Martin , C. Biard , et al. 2025. “Continental Patterns of Phenotypic Variation Along Replicated Urban Gradients: A Mega‐Analysis.” Ecology Letters 28: e70180. 40704628 10.1111/ele.70180 PMC12288444

[ele70419-bib-0143] Thompson, M. J. , and J. Morand‐Ferron . 2019. “Food Caching in City Birds: Urbanization and Exploration Do Not Predict Spatial Memory in Scatter Hoarders.” Animal Cognition 22: 743–756. 31161364 10.1007/s10071-019-01271-4

[ele70419-bib-0144] Trappes, R. , B. Nematipour , M. I. Kaiser , et al. 2022. “How Individualized Niches Arise: Defining Mechanisms of Niche Construction, Niche Choice, and Niche Conformance.” Bioscience 72: 538–548. 35677293 10.1093/biosci/biac023 PMC9169896

[ele70419-bib-0145] Trikalinos, T. A. , and J. P. A. Ioannidis . 2005. “Assessing the Evolution of Effect Sizes Over Time.” In Publication Bias in Meta‐Analysis, 241–259. John Wiley & Sons, Ltd.

[ele70419-bib-0146] Tüzün, N. , S. Müller , K. Koch , and R. Stoks . 2017a. Data for: Pesticide‐Induced Changes in Personality Depend on the Urbanization Level [Dataset]. 10.6084/m9.figshare.5483875.

[ele70419-bib-0147] Tüzün, N. , S. Müller , K. Koch , and R. Stoks . 2017b. “Pesticide‐Induced Changes in Personality Depend on the Urbanization Level.” Animal Behaviour 134: 45–55.

[ele70419-bib-0148] Uchida, K. , R. V. Blakey , J. R. Burger , D. S. Cooper , C. A. Niesner , and D. T. Blumstein . 2021. “Urban Biodiversity and the Importance of Scale.” Trends in Ecology & Evolution 36: 123–131. 33168154 10.1016/j.tree.2020.10.011

[ele70419-bib-0149] Valladares, F. , S. Matesanz , F. Guilhaumon , et al. 2014. “The Effects of Phenotypic Plasticity and Local Adaptation on Forecasts of Species Range Shifts Under Climate Change.” Ecology Letters 17: 1351–1364. 25205436 10.1111/ele.12348

[ele70419-bib-0150] Vardi, R. , and O. Berger‐Tal . 2022a. Data for: Environmental Variability as a Predictor of Behavioral Flexibility in Urban Environments [Dataset]. 10.5061/dryad.83bk3j9sx.

[ele70419-bib-0151] Vardi, R. , and O. Berger‐Tal . 2022b. “Environmental Variability as a Predictor of Behavioral Flexibility in Urban Environments.” Behavioral Ecology 33: 573–581.

[ele70419-bib-0152] Viechtbauer, W. 2010. “Conducting Meta‐Analyses in R With the Metafor Package.” Journal of Statistical Software 36: 1–48.

[ele70419-bib-0153] Vincze, E. 2014. Data for: A Comparison of Problem‐Solving Success Between Urban and Rural House Sparrows [Dataset]. 10.5061/dryad.2rbnzs82d.

[ele70419-bib-0154] Vincze, E. 2016. Data for: Habituation to Human Disturbance is Faster in Urban Than Rural House Sparrows [Dataset]. 10.5061/dryad.2rbnzs82d.

[ele70419-bib-0155] Vincze, E. , S. Papp , B. Preiszner , G. Seress , V. Bókony , and A. Liker . 2016. “Habituation to Human Disturbance Is Faster in Urban Than Rural House Sparrows.” Behavioral Ecology 27: 1304–1313.

[ele70419-bib-0156] Westneat, D. F. , J. Wright , and N. J. Dingemanse . 2015. “The Biology Hidden Inside Residual Within‐Individual Phenotypic Variation.” Biological Reviews 90: 729–743. 25080034 10.1111/brv.12131

[ele70419-bib-0157] Wilson, A. J. , D. Réale , M. N. Clements , et al. 2010. “An Ecologist's Guide to the Animal Model.” Journal of Animal Ecology 79: 13–26. 20409158 10.1111/j.1365-2656.2009.01639.x

[ele70419-bib-0158] Yovel, Y. 2020. Data for: Urban Bat Pups Take After Their Mothers and Are Bolder and Faster Learners Than Rural Pups [Dataset]. 10.5061/dryad.2rbnzs82d. PMC8422611 34493290

