# Supplementary material for: Phenotypic Flexibility in the City: A Meta‐Analysis on Variation
Source: Ecol Lett. 2026 Aug 16;29(8):e70419. doi: 10.1111/ele.70419 (PMC13477745; doi:10.1111/ele.70419)
Supplement: Supplementary file 1 [file ELE-29-0-s001.docx]

Supplementary information

**Phenotypic flexibility in the city: A meta-analysis on variation**

**Authors:** Jules Petit^1,2,#^, Melanie Dammhahn^1,2^

**Affiliations:**

^1^Department of Behavioural Biology, University of Münster

^2^Joint Institute for Individualisation in a Changing Environment (JICE), University of Münster and Bielefeld University

^#^ Corresponding author: [jules.petit1@outlook.com](mailto:jules.petit1@outlook.com) (JP)

**ORCID:** Jules Petit (0009-0008-4061-8346); Melanie Dammhahn (0000-0003-0557-740X)

Table of Contents

***This file contains supplementary figures, supplementary tables, and supplementary text.***

[Appendix - Material and methods	2](#_Toc219740982)

[Appendix - Results	11](#_Toc219740983)

[Overall meta-analysis of lnCVR (variance from AD-LMMs)	11](#_Toc219740984)

[Meta-analysis of lnCVR per trait (variance from AD-LMMs)	13](#_Toc219740985)

[Meta-analysis of lnCVR per behaviour (variance from AD-LMMs)	14](#_Toc219740986)

[Meta-analysis of lnCVR of each moderator separetely (variance from AD-LMMs)	14](#_Toc219740987)

[Sensitivity analysis	19](#_Toc219740988)

[IOM-LMM versus AD-LMM	19](#_Toc219740989)

[Bird versus all other taxa combined	21](#_Toc219740990)

[Single study effect	21](#_Toc219740991)

[Publication bias	24](#_Toc219740992)

[Appendix – PRISMA EcoEvo checklist	25](#_Toc219740993)

[Appendix - Literature	36](#_Toc219740994)

Appendix - Material and methods

**Text S1. Scoping and search term development**

We first identified few studies fitting the two main requirements for the meta-analysis (e.g. urban versus non-urban populations comparisons, repeated measurement for labile traits). Using Google Scholar and the bibliography in these papers, we found 13 suitable papers (Text S2). Based on these papers, we established a first keyword query (*Query 1*).

*Query 1:*

*All Fields =“phenotypic plasticity” OR repeata* OR consistenc*, “inter-individual” or flexibilit* or “individual plasticity” or “animal personality”) AND Topic = (urban* or rural*)*

Using *Query 1* in Web of Science with specific categories (Text S3), we extracted 2,864 papers (13.12.2022) and recovered all papers mentioned above except Møller & Tryjanowski (2014). In addition, we assessed the suitability of *Query 1* to yield relevant papers. We sorted the papers by author’s name and screened 1,000 papers using five categories: paper with non-wild animals, paper without repeated measurements, paper with urban or non-urban populations, paper with urban and non-urban populations, and paper of unknown category due to lack of methodological detail. From this categorisation, the hit rate was at 3.5%. To improve it and facilitate the screening process, exclusion keywords were added to the search. We made two groups: i) papers which were highly irrelevant using the strong exclusion criteria ‘paper with non-wild animals’ (n = 767) and, ii) papers which were highly relevant ‘paper with wild animals, studying urban or non-urban populations, and with repeated measurements for labile traits’ (n = 35).

For each group, we calculated the frequencies of the words used in the title and abstract separately using *R* packages *wordcloud* (Fellows 2018) and *wordcloud2* (Lang & Chien 2018). Six words were used repeatedly in the irrelevant paper group and never used in the relevant paper group (e.g., planning, water management, emission, policy, carbon, and child). We added them in the query as exclusion words. We also added important missing keywords such as “intra-individual”, “repeated disturbance”, “repeated exposure”, and “repeated trial”. We did not use “repeat*” because it added many irrelevant articles. Based on these refinements *Query 2* was developed.

*Query 2:*

*TOPIC = "phenotypic plasticity" or repeata* or consistenc* or "inter-individual" or "intra-individual" or flexibilit* or "individual plasticity" or "animal personality" or "repeated disturbance*" or "repeated exposure*" or "repeated trial*" AND ABSTRACT = urban* or rural* NOT ABSTRACT = "planning" or "water management" or emission* or polic* or carbon* or child**

*Query 2* yielded 1,509 papers in Web of Science with specific categories (Text S4) and retrieved 12 original papers from the Google Scholar search (Møller & Tryjanowski 2014 was still missing) as well as the 35 papers from the 1,000 papers screening. Thus, Query 2 was more specific and effective to find relevant papers and was used for the literature search.

**Text S2. List of suitable papers from Google Scholar search**

The following papers have been used for finding original keywords for the first search (Carrete & Tella 2013, 2017; Charmantier *et al.* 2017; Davies & Sewall 2016; Evans *et al.* 2010; Hardman & Dalesman 2018; Kaiser *et al.* 2018; Miranda *et al.* 2013; Møller & Tryjanowski 2014; Papp *et al.* 2015; Scales *et al.* 2011; Tabh *et al.* 2022; Uchida *et al.* 2020).

**Text S3. *Web of sciences categories* for *Query 1***

Environmental Sciences, Environmental studies, Ecology, Urban studies, Marine Freshwater Biology, Ornithology, Agriculture Multidisciplinary, Veterinary Sciences, Multidisciplinary Sciences, Zoology, Biodiversity Conservation, Psychology Multidisciplinary, Neurosciences, Physiology, Psychology Multidisciplinary, Behavioural Sciences, Biology, Evolutionary Biology, Parasitology, Immunology, Toxicology, Psychology Biological, Endocrinology Metabolism, Entomology, Reproductive biology.

**Text S4. *Web of sciences categories* for *Query 2* and final search**

Environmental Sciences, Environmental studies, Ecology, Urban studies, Marine Freshwater Biology, Zoology, Psychology Multidisciplinary, Biology, Evolutionary Biology, Behavioural Sciences, Veterinary Sciences, Neurosciences, Entomology, Agriculture Multidisciplinary, Ornithology, Physiology, Toxicology, Reproductive Biology, Immunology, Parasitology, Multidisciplinary Sciences, Biodiversity Conservation.

**Table S1. Final search terms used per literature database**

| **Database** | **Search used** | **Specific filter used** |
| --- | --- | --- |
| Web of Sciences Collection | **TOPIC** = "phenotypic plasticity" or repeata* or consistenc* or "inter-individual" or "intra-individual" or flexibilit* or "individual plasticity" or "animal personality" or "repeated disturbance*" or "repeated exposure*" or "repeated trial*"  AND **ABSTRACT** = urban* or rural*  NOT **ABSTRACT** = "planning" or "management" or emission* or polic* or carbon* or child* | In Web of Science Core Collection.  All dates until 10.03.2024  *Web of sciences categories:* see Text S4 |
| Scopus | **(article, abstract, keyword)** = "phenotypic plasticity" or repeata* or consistenc* or "inter-individual" or "intra-individual" or flexibilit* or "individual plasticity" or "animal personality" or "repeated disturbance*" or "repeated exposure*" or "repeated trial*"  AND **ABSTRACT** = urban* or rural*  NOT **ABSTRACT** = "planning" or "management" or emission* or polic* or carbon* or child* | All dates until 10.03.2024  *Subject area*: ‘Environmental Sciences’, ‘Agricultural and Biological Sciences’, ‘Psychology’, ‘Multidisciplinary’, ‘Decision Sciences’, ‘Immunology and Microbiology’, ‘Pharmacology, Toxicology and Pharmaceutics’, ‘Neuroscience’, ‘Veterinary’, ‘Undefined’ |
| ProQuest | "phenotypic plasticity" or repeata* or consistenc* or "inter-individual" or "intra-individual" or flexibilit* or "individual plasticity" or "animal personality" or "repeated disturbance*" or "repeated exposure*" or "repeated trial*” **IN abstract – ABSTRACT°** AND urban* or rural* **IN abstract – ABSTRACT°** NOT "planning" or "management" or emission* or polic* or carbon* or child* **IN abstract – ABSTRACT°** | All dates until 10.03.2024 |
| EBSCOhost Open Dissertations | ("phenotypic plasticity" or repeata* or consistenc* or "inter-individual" or "intra-individual" or flexibilit* or "individual plasticity" or "animal personality" or "repeated disturbance*" or "repeated exposure*" or "repeated trial*") AND (urban* or rural*) NOT ("planning" or "management" or emission* or polic* or carbon* or child*) | Publication data: 1970-2020 |
| Open Grey | ("phenotypic plasticity" or repeata* or consistenc* or "inter-individual" or "intra-individual" or flexibilit* or "individual plasticity" or "animal personality" or "repeated disturbance*" or "repeated exposure*" or "repeated trial*") AND (urban* or rural*) NOT ("planning" or "management" or emission* or polic* or carbon* or child*) | All dates until 10.03.2024 |

**Figure S1.** **PRISMA chart** of the three screening phases and after the contact phase with criteria table

**Text S5. Contact procedure**

We used a template letter inspired from Foo *et al.* (2021). This first contacting phase added 9 potential papers resulting in 122 papers. All authors were contacted at least three times over a one year and a half period to increase the chance of responses. In total, we collected 39 dataset, and 2 researchers were willing to run our script without sharing data. After the last contacting phase and a deep check of the data we could use 33 datasets to estimate the among- and within-individual variance partitions. We would like to emphasize that, unfortunately, about half of our emails did not receive any answer.

**Table S2. Functional definitions** **of the phenotypic traits incorporated in the meta-analysis**

| **Phenotypic traits** | **Definitions** |
| --- | --- |
| BEHAVIOUR | |
| Aggression | Agonistic behaviour including all behaviours associated with the contest or struggle between individuals (King 1973). |
| Boldness | Behaviour expressed in any risky situation (but not new situations) where direct confrontation can be avoided (Réale *et al.* 2007). |
| Activity/Exploration | Behaviour directed toward acquiring information about the environment (Meyer 1998).  We included activity as disentangle information gathering from pure movement is difficult and original paper did not make this difference (Crawley 1985; Paré & Glavin 1993). |
| Foraging | Behaviour related to food acquisition. |
| Neophilia | Refer to the tendency to approach novel stimuli. Differ from ‘Neophobia’ as it should be in a familiar/non-dangerous situation (Greenberg & Mettke-hofmann 2001; Mettke‐Hofmann *et al.* 2002). |
| Neophobia | (1-Non neophobic) the mere preference for feeding on or visiting familiar foods, objects, or places.  (2-Neophobic) the aversion or fear demonstrated to the same novel stimuli (e.g., new object, new colour, new type of food) in a foraging context.  (Greenberg & Mettke-hofmann 2001; Mettke‐Hofmann *et al.* 2002) |
| COGNITION | |
| Innovation  (also called problem solving or innovative problem solving) | Adoption of behaviours that allow individuals to exploit newly available, previously used or familiar resources in a new way (Greenberg 2003). **One** **apparatus used once to avoid learning effect. Per definition can be only contextual repeatability.**  We did not discard individuals that did not solve the problem because individuals who took the maximum time are still informative of the individual variation happening in the population (experimental artefact from the length of test) |
| Associative learning  (also called learning or repetitive problem solving) | The ability of an organism to learn the correct association in a stimulus-reward contingencies (Griffin *et al.* 2015). **One apparatus re-use multiple time (minimum re-use is 2-trial for repeatability)**  We did not discard individuals that did not solve the problem because individuals who took the maximum time are still informative of the individual variation happening in the population (experimental artefact from the length of test) |
| Reversal learning | The ability of an organism to learn a reverse previously learned association in a stimulus-reward contingencies (Williams 1942). |
| MORPHOLOGY | |
| Body mass | Weight of the individual |
| Testis size | Width of the testis |
| Fat score | Amount of subcutaneous fat scaled from 0 to 8 (Dominoni *et al.* 2015) |
| PHYSIOLOGY | |
| Corticosterone baseline | Corticosterone measure without any intentional stress induced on animals (following the expertise of the authors of the paper) |
| Corticosterone acute stress | Corticosterone measure after an intentional stress induced on animals after a relatively short interval (following the expertise of the authors of the paper) |
| Corticosterone long stress | Corticosterone measure after an intentional stress induced on animals after a relatively long interval (following the expertise of the authors of the paper) |
| Luteinizing hormone | Luteinizing hormone concentration in plasma |
| Body surface temperature | Body surface temperature is measured on the periorbital region of the eye |

**Text S6.** **Nomenclature used in the papers to qualify urban or non-urban habitats**

Urban habitats were referred as “urban”, “stable urban”, “dynamic urban”, “city”, “urban zone”, and “suburban”. Non-urban habitats were referred as “forest”, “rural”, “extra-urban”, “artificial pond”, “crops”, “country”, “woodland”, “agricultural”, “lake”, and “artificial reservoir”. More detailed regarding how authors described their study sites can be found in the excel file ‘Data/0-Literature review/*Summary screening process from 2^nd^ screening.xlsx*’ in the dryad depository.

**Text S7. Example of categorisation of measurement intervals**

To illustrate the categorisation for a bird species, repetitions made across two years (≥ two breeding seasons) would be considered a long interval. Repetitions made between the first and the second half of one breeding season would be considered medium whereas repetitions within a shorter period within one breeding season (e.g. one week) would be considered short. Repetitions over consecutive days would be considered very short.

**Text S8.** **Justification of the use of only continuous and count data and the use of Gaussian error distribution for count data**

We used only continuous and count data in our meta-analysis as binomial data cannot be fitted in a meaningful way using Gaussian error distribution. We chose to not fit other distributions such as Poisson or Binomial error distribution because it transforms the data to another realm (probabilistic, Zuur *et al.* 2009b) with different variance properties which would hamper our ability to draw straightforward conclusions in the meta-analysis. It is accepted that count data can be analysed using Gaussian error distribution as estimates remain unbiased although residual heterogeneity might occur (Schielzeth *et al.* 2020; Zuur *et al.* 2009a). Therefore, we verified homogeneity of residuals for all our models via the function *check_model()* from the *performance* package (Lüdecke *et al.* 2021). We would like to stress that, here, models are used solely as a tool to calculate descriptive statistics such as overall average, inter-group variance (e.g. individual identity) and inter-observation variance (e.g. residuals). We did not perform any model selection as the only goal of these models was to estimate the variance.

**Text S9. Procedure of mean and variance extraction check**

Usually, in meta-analysis, authors extract estimates based on descriptive statistics rather than using a variance partitioning approach. Therefore, we also extracted the mean and total variance with the basic function “mean()” and “var()” in *R* (v.4.4.0; R Core Team 2022) for each urban and non-urban dataset separately. We checked that the mean and total variance estimates from our models did not deviate > 5% compared to the basic estimation method. We considered that 5% deviation is an adequate threshold to validate how the models accomplish a solid estimation.

Mean and variance estimates calculated via the ‘intercept-only’ linear mixed effects models (IO-LMMs) were compared to the variance estimates obtained via classical mean and variance computations (*mean()* and *var()* function used in *R* on the raw dataset) to verify that our models accurately estimated our parameters. For adjusted linear mixed effects models (AD-LMMs), we could check only the variance since the inclusion of fixed factors deviated the model intercept from the ‘true’ mean. We calculated a coefficient of deviation (Δ_LMM_) for each mean and variance estimates separately (see *Equation 1*).

(*Equation 1*) Δ_LMM_=$\frac{X_{model}-X_{classical}}{X_{classical}}\times100$, *where X_model_ represents the mean or variance estimated from the model and X_classical_ represents the mean or variance estimated from the classical computations.*

We aimed that our LMM estimates did not deviate for more than 5% from the classical estimates.

When 5% < Δ_LMM_ < 6%, we checked the presence of ‘Influential Observations’ using the function *check_model()* from the *performance* package (Lüdecke *et al.* 2021).

- **If influential observations had a Leverage < 0.5,** we checked the presence of outliers using the function *check_model()* and looking at the ‘Posterior Predictive Check’.
  - **If a clear right or left-skew distribution was present,** we accepted the estimates as it is still very close to the 5% threshold of acceptation. We decided to not remove the outliers as we assumed that authors checked the biological validity of these outliers.
  - **If we did not observe any outliers,** the estimate was classified as unusable as we were not able to run a LMM giving an acceptable accuracy.
- **If influential observations had a Leverage ≥ 0.5,** we rerun the model without the influential observations. In this case, we accepted the deletion of the influential observations even if the observations seemed biologically relevant. We prefer to stay conservative in our estimations with reduced amount of information on variation than calculate inaccurate variance estimations. The maximum deletion applied in a dataset was five datapoints.
  - **If the new estimate reached the 5% threshold of acceptation**, the new estimate was accepted.
  - **If the new estimate did not reach the 5% threshold of acceptation**, the estimate was classified as unusable as we were not able to run a LMM giving an acceptable accuracy.

When Δ_LMM_ > 6%, we performed the same procedure although if a clear right or left skew was observed we did not accept the estimates and instead tried to improve Δ_LMM_ by removing outliers.

We used ‘Homogeneity of variance’ to select which value were to be prioritized for removal. Observations with high fitted values and high standard deviation residuals were removed in priority.

We noticed that outliers without repeated values had strong effect on the variance estimation. In addition, dataset with Poisson looking-like residual’s distribution had more chance to cause problems in variance estimation as well.

In addition, when among-individual variation was estimated to be zero, we removed those estimates from any analyses (among- and within-individual variation analyses) since they caused strong outliers in the meta-analytic models and that such null estimates are most likely due to poor data resolution.

AD-LMM estimates were automatically discarded when IO-LMM did not estimate the mean respecting the ‘mean check’ threshold and that the same dataset was used in both modelling approach for a more conservative method.

After excluding observations with inappropriate deviation (Table S3), on average for the IO-LMM, the overall absolute ‘mean’ divergence was |0.52|% with a maximum positive divergence of 4.20% and a maximum negative divergence of 4.89%. The overall absolute ‘variance’ divergence was |2.00|% with a maximum positive divergence of 5.62% and a maximum negative divergence of 5.36%. For the AD-LMM, the overall absolute ‘variance’ divergence was |1.78|% with a maximum positive divergence of 5.87% and a maximum negative divergence of 5.81%. We did not calculate the divergence for the ‘mean’ since the inclusion of fixed factors deviated the model intercept from the ‘true’ mean.

**Table S3. Summary of mean and variance check for intercept-only linear mixed effects models (IO-LMMs) and adjusted linear mixed effects models (AD-LMMs)**

| **Paper** | **Trait** | **Data point removed** | **Conclusion** |
| --- | --- | --- | --- |
| **For IO-LMM** | | | |
| Dominoni *et al.* 2015 | Luteinizing, Urban | NA | Accepted, could not fix but because high right skew and close to 5% |
| Dominoni *et al.* 2015 | Body mass, Urban | 1 | Corrected |
| Garitano-Zavala *et al.* 2022 | Latency to solve, NonUrban | NA | Accepted, could not fix but close to 5% |
| Huang *et al.* 2020 | Number of flight, Urban | NA | Accepted, could not fix but close to 5% |
| Huang *et al.* 2020 | Number of flight, NonUrban | NA | Accepted, could not fix but close to 5% |
| Huang *et al.* 2020 | Number of hop, NonUrban | NA | Accepted, could not fix but close to 5% |
| Huang *et al.* 2020 | Active scanning, NonUrban | NA | Accepted, could not fix but close to 5% |
| Huang *et al.* 2020 | Prop. Perch visited, Urban | NA | Accepted, could not fix but close to 5% |
| Huang *et al.* 2020 | Prop. Ground visited, NonUrban | NA | Accepted, could not fix but close to 5% |
| Huang *et al.* 2020 | Latency score differences novel object, NonUrban | 1 | Accepted, outlier removed because out of range |
| Mazza & Guenther 2021 | Boldness, urban | NA | Accepted, could not fix but close to 5% |
| Petit & Dammhahn 2024 unpubl. | Prop. activity, WM, NonUrban | 2 | Corrected |
| Petit & Dammhahn 2024 unpubl. | Prop. activity, YM, Urban | NA | Accepted, could not fix but close to 5% |
| Petit & Dammhahn 2024 unpubl. | Body mass, WM, NonUrban | 3 | Corrected |
| Petit & Dammhahn 2024 unpubl. | Boldness, YM, NonUrban | NA | Discarded, could not fix mean |
| Petit & Dammhahn 2024 unpubl. | Boldness, YM, Urban | NA | Discarded, could not fix the variance and mean |
| Rimbach & Dammhahn 2024 unpubl. | CORT, YM, Urban | NA | Discarded, could not fix the variance |
| Rimbach & Dammhahn 2024 unpubl. | CORT, WM, NonUrban | NA | Discarded, could not fix the variance and mean |
| Rimbach & Dammhahn 2024 unpubl. | CORT, BV, NonUrban | 2 | Corrected |
| Solaro & Sarasola 2019 | Innovation, latency, NonUrban | NA | Discarded, could not fix the variance and mean |
| Stansell *et al.* 2022 | Boldness, NonUrban | 6 | Corrected |
| Thompson & Morand-Ferron 2019 | Latency to solve, Nonurban, Juv | NA | Accepted, could not fix but because high right skew and close to 5% |
| Thompson & Morand-Ferron 2019 | Incorrect cache, Nonurban, Juv | NA | Accepted, could not fix but because high right skew and close to 5% |
| Thompson *et al.* 2018 | Hop duration, NonUrban, Adult | 1 | Corrected |
| **For AD-LMM** | | | |
| Bar‐Ziv *et al.* 2023 | DFL, Jackdal, Urban | NA | Discard, could not fix variance |
| Dominoni *et al.* 2015 | Luteinizing, Urban | NA | Accepted, could not fix but because high right skew and close to 5% |
| Dominoni *et al.* 2015 | Body mass, Urban | 1 | Corrected |
| Huang *et al.* 2020 | Number of flight, Urban | NA | Accepted, could not fix but close to 5% |
| Huang *et al.* 2020 | Number of flight, NonUrban | NA | Accepted, could not fix but close to 5% |
| Huang *et al.* 2020 | Number of hop, NonUrban | NA | Accepted, could not fix but close to 5% |
| Huang *et al.* 2020 | Active scanning, NonUrban | NA | Accepted, could not fix but close to 5% |
| Huang *et al.* 2020 | Prop. Perch visited, Urban | NA | Accepted, could not fix but close to 5% |
| Huang *et al.* 2020 | Prop. Ground visited, NonUrban | NA | Accepted, could not fix but close to 5% |
| Huang *et al.* 2020 | Number of hop, Urban | 2 | Corrected |
| Huang *et al.* 2020 | Latency score differences novel object, NonUrban | 1 | Accepted, outlier removed because out of range |
| Petit & Dammhahn 2024 unpubl. | Prop. activity, WM, NonUrban | 3 | Corrected |
| Petit & Dammhahn 2024 unpubl. | Body mass, WM, NonUrban | 3 | Corrected |
| Petit & Dammhahn 2024 unpubl. | Boldness, YM, NonUrban | NA | Discarded, could not fix mean in IO-LMM |
| Petit & Dammhahn 2024 unpubl. | Boldness, YM, Urban | NA | Discarded, could not fix mean in IO-LMM |
| Petit & Dammhahn 2024 unpubl. | Prop. activity, YM, nonurban | NA | Accepted, could not fix but a little skew but close to 5% |
| Petit & Dammhahn 2024 unpubl. | Mass, urban, YM | NA | Accepted, could not fix but a little skew but close to 5% |
| Prasher *et al.* 2019 | Latency, nonurban, juvenile | NA | Accepted, could not fix but because binomial like skew and close to 5% |
| Rimbach & Dammhahn 2024 unpubl. | CORT, WM, NonUrban | NA | Discarded, could not fix mean in IO-LMM and convergence problem |
| Rimbach & Dammhahn 2024 unpubl. | CORT, BV, NonUrban | 2 | Corrected (only for AD-LMM) |
| Rimbach & Dammhahn 2024 unpubl. | Mass, BV, urban | 2 | Corrected |
| Solaro & Sarasola 2019 | Innovation, latency, NonUrban | NA | Discarded, could not fix mean in IO-LMM |
| Stansell *et al.* 2022 | Boldness, NonUrban | NA | Discarded nonurban because off with 20% |
| Thompson & Morand-Ferron 2019 | Latency to solve, Nonurban, Juv | NA | Accepted, could not fix but because high right skew and close to 5% |
| Thompson & Morand-Ferron 2019 | Incorrect cache, Nonurban, Juv | NA | Accepted, could not fix but because high right skew and close to 5% |
| Thompson *et al.* 2018 | Hop duration, NonUrban, Adult | 1 | Corrected |
| Thompson *et al.* 2018 | Number of tree visited, nonurban, adult | NA | Accepted, could not fix but close to 5% |
| Thompson *et al.* 2018 | Flight duration, nonurban, adult | NA | Accepted, could not fix but a little skew but close to 5% |
| Thompson *et al.* 2018 | Hop duration, urban, adult | 1 | Corrected |
| Tüzün *et al.* 2017 | Mass, urban | 2 | Corrected |

**Table S4. Summary of estimates discarded due to impossibility of variance estimation**

| **Paper** | **Traits** | **Habitat** | **Model type** |
| --- | --- | --- | --- |
| Bar‐Ziv *et al.* 2023 | Boldness:FID:jackdal | urban  urban & non-urban | IO-LMM  AD-LMM |
| Dominoni *et al.* 2015 | Testis size | urban & non-urban  urban & non-urban | IO-LMM  AD-LMM |
| Garitano-Zavala *et al.* 2022 | Innovation: Latency to solve | non-urban | IO-LMM |
| Grunst *et al.* 2014 | CORT_contextA | urban & non-urban  non-urban | IO-LMM  AD-LMM |
| Harten *et al.* 2021 | Boldness: prop. Landing (female) | non-urban  non-urban | IO-LMM  AD-LMM |
| Heppner *et al.* 2023 | Body mass (Juvenile) | urban & non-urban | IO-LMM |
| Huang *et al.* 2020 | Neophobia: latency score differences | urban  urban | IO-LMM  AD-LMM |
| Huang *et al.* 2020 | CORT_contextB | urban  urban | IO-LMM  AD-LMM |
| Kozlovsky *et al.* 2017 | Neophobia: Latency to approach | urban & non-urban | IO-LMM |
| Kozlovsky *et al.* 2017 | Associative learning: Latency to solve | urban | IO-LMM |
| Mazza & Guenther 2021 | Activity/exploration: prop. Of activity | urban | AD-LMM |
| Mazza & Guenther 2021 | Innovation: Latency to solve | urban | AD-LMM |
| Ouyang *et al.* 2019 | CORT_temporal (Juvenile) | urban & non-urban | IO-LMM |
| Ouyang *et al.* 2019 | Body mass (Juvenile) | urban & non-urban | IO-LMM |
| Papp *et al.* 2015 | Innovation: Latency to solve | non-urban  non-urban | IO-LMM  AD-LMM |
| Petit & Dammhahn 2024 unpubl. | Boldness: Latency to emerge (*Apodemus sylvaticus*) | urban  urban | IO-LMM  AD-LMM |
| Prasher *et al.* 2019 | Innovation: Latency to solve (Juvenile) | urban & non-urban  urban & non-urban | IO-LMM  AD-LMM |
| Prasher *et al.* 2019 | Innovation: Latency to solve (Adult) | urban | AD-LMM |
| Rimbach & Dammhahn 2024 | CORT (*Myodes glareolus*) | urban | IO-LMM  AD-LMM |
| Tabh *et al.* 2022 | Body surface temperature | non-urban | IO-LMM |
| Thompson & Morand-Ferron 2019 | Associative learning: Latency to solve (Juvenile) | urban & non-urban  urban & non-urban | IO-LMM  AD-LMM |
| Thompson & Morand-Ferron 2019 | Associative learning: Latency to solve (Adult) | urban & non-urban  non-urban | IO-LMM  AD-LMM |
| Thompson & Morand-Ferron 2019 | Associative learning: Incorrect cache (Juvenile) | urban & non-urban  non-urban | IO-LMM  AD-LMM |
| Thompson & Morand-Ferron 2019 | Associative learning: Incorrect cache (Adult) | urban & non-urban  urban | IO-LMM  AD-LMM |
| Thompson *et al.* 2018 | Activity/exploration: Flight duration (Juvenile) | non-urban  non-urban | IO-LMM  AD-LMM |
| Thompson *et al.* 2018 | Activity/exploration: Flight duration (Adult) | urban | IO-LMM |
| Thompson *et al.* 2018 | Activity/exploration: Hop duration (Juvenile) | non-urban | IO-LMM |
| Thompson *et al.* 2018 | Activity/exploration: Number of tree (Juvenile) | non-urban | IO-LMM |

**Text S10. Sample size of IO-LMMs and AD-LMMs separately**

For IO-LMMs only, the dataset included 79 paired urban–non-urban estimates from 20 species and 27 studies. Of these 79 comparisons, 57 corresponded to comparisons of behavioural trait (20 studies), 10 corresponded to cognitive traits (7 studies), 3 were comparisons of physiological trait (2 studies), 9 were comparisons of morphological traits (7 studies). Last, within the 57 behavioural traits, 15 corresponded to comparisons of boldness (11 studies), 29 corresponded to comparisons of activity/exploration (11 studies), 8 corresponded to comparisons of aggression (3 studies), 1 corresponded to neophilia (1 study), 2 corresponded to neophobia (2 studies), and 2 corresponded to foraging (2 studies). For AD-LMMs only, the dataset included 85 paired urban–non-urban estimates from 21 species and 29 studies (list reference here). Of these 85 comparisons, 58 corresponded to comparisons of behavioural trait (19 studies), 10 were comparisons of cognitive trait (6 studies), 11 were comparisons of morphological traits (9 studies) and 6 were comparisons of physiological trait (5 studies). Last, within the 58 behavioural traits, 31 corresponded to comparisons of activity/exploration (10 studies), 8 corresponded to comparisons of aggression (3 studies) and 13 corresponded to comparisons of boldness (10 studies), 1 corresponded to neophilia (1 study), 3 corresponded to neophobia (3 studies), and 2 corresponded to foraging (2 studies).

**Text S11. Copyright usage**

Pictures from Nicolas Venner (material link: <https://flic.kr/p/2phKm3X> and <https://flic.kr/p/2ppn7XJ>) and Gonzalo Arias (material link: <https://flic.kr/p/2hK3YoE>) are in public domain and copyright free (CC0 1.0 Universal). Picture from Vipin Baliga follows the copyright CC BY 2.0 and is free to share and adapt even for commercially purpose (material link: <https://flic.kr/p/nsgk5Z>). Picture of Tim Worfolk follows the copyright CC-BY-ND 2.0 and is free to share without derivatives even for commercially purpose (material link: <https://flic.kr/p/2oYSvD7>). Picture of Brian Gratwicke follows the copyright CC BY 2.0 and is free to share and adapt even for commercially purpose (material link: <https://flic.kr/p/a8poSD>).

Appendix - Results

Overall meta-analysis of lnCVR (variance from AD-LMMs)

**Figure S2.** **Overall model estimates for lnCVR assessing differences between urban and non-urban populations for among-individual variation calculated from AD-LMMs (Model 1).** Positive values on the x axis represent higher among-individual variation in urban populations compared to non-urban populations whereas negative values represent the opposite phenomena. The large grey point represents the overall model estimate. The thick black line shows the 95% confidence intervals. The thin black line shows the 95% prediction intervals. Transparent small grey dots show the raw data (their size is scaled according to their sample size from which they were estimated). k is the number of observations supplemented with the number of studies between brackets.

**Table S5. Meta-analytic model estimates explaining overall variation in lnCVR of among-individual variation** (Model 1, i.e. differences in among-individual variance between urban and non-urban populations calculated using AD-LMMs). CI stands for confidence intervals, PI for prediction intervals, I² for heterogeneity and k for the number of observations.

| **Fixed Effect** | | | | | |
| --- | --- | --- | --- | --- | --- |
| Intercept | estimate | 95% CI | | 95% PI | |
|  | -0.031 | -0.174 | 0.112 | -0.972 | 0.910 |
|  |  |  | |  | |
| **Random effect & residual variance** | | | | | |
| Study ID | estimate | *I*² | | k | |
|  | 0.000 | 0.00 | | 29 | |
| Phylogeny | 0.000 | 0.00 | | 21 | |
| Species ID | 0.045 | 17.95 | | 21 | |
| Observation ID | 0.180 | 71.86 | | 85 | |
|  | *I*²_total_: | 89.81 | |  | |

**Figure S3.** **Overall model estimates for lnCVR assessing differences between urban and non-urban populations for within-individual variation calculated from AD-LMMs (Model 3).** Positive values on the x axis represent higher within-individual variation in urban populations compared to non-urban populations whereas negative values represent the opposite phenomena. The large grey point represents the overall model estimate. The thick black line shows the 95% confidence intervals. The thin black line shows the 95% prediction intervals. Transparent small grey dots show the raw data (their size is scaled according to their sample size from which they were estimated). k is the number of observations supplemented with the number of studies between brackets.

**Table S6. Meta-analytic model estimates explaining overall variation in lnCVR of within-individual variation** (Model 3, i.e. differences in within-individual variance between urban and non-urban populations calculated using AD-LMMs). CI stands for confidence intervals, PI for prediction intervals, I² for heterogeneity and k for the number of observations.

| **Fixed Effect** | | | | | |
| --- | --- | --- | --- | --- | --- |
| Intercept | estimate | 95% CI | | 95% PI | |
|  | -0.059 | -0.159 | 0.041 | -0.808 | 0.690 |
|  |  |  | |  | |
| **Random effect & residual variance** | | | | | |
| Study ID | estimate | *I*² | | k | |
|  | 0.000 | 0.00 | | 29 | |
| Phylogeny | 0.000 | 0.00 | | 21 | |
| Species ID | 0.011 | 6.86 | | 21 | |
| Observation ID | 0.133 | 85.67 | | 85 | |
|  | *I*²_total_: | 92.52 | |  | |

Meta-analysis of lnCVR per trait (variance from AD-LMMs)

**Table S7. Meta-analytic model estimates explaining variation in lnCVR of among-individual variation per phenotypic trait** (Model 2, i.e. differences in among-individual variance per phenotypic trait between urban and non-urban populations calculated using AD-LMMs). CI stands for confidence intervals, PI for prediction intervals, I² for heterogeneity and k for the number of observations.

| **Fixed Effect** | | | | | |
| --- | --- | --- | --- | --- | --- |
| Behaviour  Cognition  Morphology  Physiology | estimate | 95% CI | | 95% PI | |
|  | -0.044  -0.210  -0.146  0.444 | -0.217  -0.554  -0.446  0.019 | 0.129  0.134  0.155  0.869 | -0.985  -1.197  -1.119  -0.575 | 0.898  0.777  0.827  1.462 |
|  |  |  | |  | |
| **Random effect & residual variance** | | | | | |
| Study ID | estimate | *I*² | | k | |
|  | 0.048 | 19.40 | | 29 | |
| Phylogeny | 0.000 | 0.00 | | 21 | |
| Species ID | 0.015 | 5.96 | | 21 | |
| Observation ID | 0.160 | 64.35 | | 85 | |
|  | *I*²_total_: | 89.72 | |  | |

**Table S8. Meta-analytic model estimates explaining variation in lnCVR of within-individual variation** **per phenotypic trait** (Model 4, i.e. differences in within-individual variance per phenotypic trait between urban and non-urban populations calculated using AD-LMMs). CI stands for confidence intervals, PI for prediction intervals, I² for heterogeneity and k for the number of observations.

| **Fixed Effect** | | | | | |
| --- | --- | --- | --- | --- | --- |
| Behaviour  Cognition  Morphology  Physiology | estimate | 95% CI | | 95% PI | |
|  | -0.067  0.045  -0.017  -0.232 | -0.192  -0.226  -0.268  -0.564 | 0.057  0.316  0.233  0.100 | -0.828  -0.753  -0.809  -1.053 | 0.694  0.844  0.774  0.589 |
|  |  |  | |  | |
| **Random effect & residual variance** | | | | | |
| Study ID | estimate | *I*² | | k | |
|  | 0.000 | 0.00 | | 29 | |
| Phylogeny | 0.000 | 0.00 | | 21 | |
| Species ID | 0.013 | 8.14 | | 21 | |
| Observation ID | 0.134 | 84.53 | | 85 | |
|  | *I*²_total_: | 92.67 | |  | |

Meta-analysis of lnCVR per behaviour (variance from AD-LMMs)

**Table S9. Meta-analytic model estimates explaining variation in lnCVR of within-individual variation per behavioural trait** (Model 5, i.e. differences in within-individual variance per behavioural trait between urban and non-urban populations calculated using AD-LMMs). CI stands for confidence intervals, PI for prediction intervals, I² for heterogeneity and k for the number of observations.

| **Fixed Effect** | | | | | |
| --- | --- | --- | --- | --- | --- |
| Activity/Exploration  Aggression  Boldness | estimate | 95% CI | | 95% PI | |
|  | -0.068  -0.044  -0.059 | -0.250  -0.366  -0.293 | 0.113  0.278  0.176 | -0.866  -0.885  -0.870 | 0.730  0.797  0.753 |
|  |  |  | |  | |
| **Random effect & residual variance** | | | | | |
| Study ID | estimate | *I*² | | k | |
|  | 0.000 | 0.00 | | 16 | |
| Phylogeny | 0.000 | 0.00 | | 13 | |
| Species ID | 0.022 | 12.90 | | 13 | |
| Observation ID | 0.136 | 81.04 | | 52 | |
|  | *I*²_total_: | 93.94 | |  | |

Meta-analysis of lnCVR of each moderator separetely (variance from AD-LMMs)

**Table S10a. Meta-analytic model estimates explaining variation in lnCVR of among-individual variation due to provenance of data** (Model 6a, i.e. differences in among-individual variance between urban and non-urban populations calculated using AD-LMMs). CI stands for confidence intervals, PI for prediction intervals, I² for heterogeneity and k for the number of observations.

| **Fixed Effect** | | | | | | |
| --- | --- | --- | --- | --- | --- | --- |
| both  field  lab | estimate | 95% CI | | 95% PI | | k |
|  | 0.002  0.023  -0.052 | -0.755  -0.248  -0.219 | 0.758  0.293  0.114 | -1.209  -0.960  -1.012 | 1.212  1.006  0.907 | 2  17  66 |
|  |  |  | | | |  |
| **Random effect & residual variance** | | | | | | |
| Study ID | estimate | *I*² | | | | k |
|  | 0.012 | 4.73 | | | | 29 |
| Phylogeny | 0.000 | 0.00 | | | | 21 |
| Species ID | 0.039 | 15.15 | | | | 21 |
| Observation ID | 0.181 | 70.22 | | | | 85 |
|  | *I*²_total_: | 90.10 | | | |  |

**Table S10b. Meta-analytic model estimates explaining variation in lnCVR of among-individual variation due to interval between measurements corrected for lifespan** (Model 6b, i.e. differences in among-individual variance between urban and non-urban populations calculated using AD-LMMs). CI stands for confidence intervals, PI for prediction intervals, I² for heterogeneity and k for the number of observations.

| **Fixed Effect** | | | | | | |
| --- | --- | --- | --- | --- | --- | --- |
| Short  Very short | estimate | 95% CI | | 95% PI | | k |
|  | -0.042  -0.025 | -0.292  -0.197 | 0.208  0.148 | -1.016  -0.981 | 0.932  0.932 | 21  64 |
|  |  |  | | | |  |
| **Random effect & residual variance** | | | | | | |
| Study ID | estimate | *I*² | | | | k |
|  | 0.000 | 0.00 | | | | 29 |
| Phylogeny | 0.000 | 0.00 | | | | 21 |
| Species ID | 0.050 | 19.40 | | | | 21 |
| Observation ID | 0.181 | 70.62 | | | | 85 |
|  | *I*²_total_: | 90.02 | | | |  |

**Table S10c. Meta-analytic model estimates explaining variation in lnCVR of among-individual variation due to age class** (Model 6c, i.e. differences in among-individual variance between urban and non-urban populations calculated using AD-LMMs). CI stands for confidence intervals, PI for prediction intervals, I² for heterogeneity and k for the number of observations.

| **Fixed Effect** | | | | | | |
| --- | --- | --- | --- | --- | --- | --- |
| Adult  Juvenile | estimate | 95% CI | | 95% PI | | k |
|  | -0.008  -0.157 | -0.158  -0.467 | 0.142  0.154 | -0.947  -1.134 | 0.931  0.821 | 73  12 |
|  |  |  | | | |  |
| **Random effect & residual variance** | | | | | | |
| Study ID | estimate | *I*² | | | | k |
|  | 0.000 | 0.00 | | | | 29 |
| Phylogeny | 0.000 | 0.00 | | | | 21 |
| Species ID | 0.040 | 16.17 | | | | 21 |
| Observation ID | 0.183 | 73.57 | | | | 85 |
|  | *I*²_total_: | 89.75 | | | |  |

**Table S10d. Meta-analytic model estimates explaining variation in lnCVR of among-individual variation due to taxa** (Model 6d, i.e. differences in among-individual variance between urban and non-urban populations calculated using AD-LMMs). CI stands for confidence intervals, PI for prediction intervals, I² for heterogeneity and k for the number of observations.

| **Fixed Effect** | | | | | | |
| --- | --- | --- | --- | --- | --- | --- |
| Amphibian  Bird  Insect  Mammal  Reptile | estimate | 95% CI | | 95% PI | | k |
|  | 0.007  -0.039  -0.204  0.094  -0.049 | -0.786  -0.252  -0.651  -0.233  -0.727 | 0.799  0.174  0.243  0.421  0.630 | -1.248  -1.035  -1.275  -0.932  -1.235 | 1.261  0.957  0.867  1.120  1.137 | 2  52  14  13  4 |
|  |  |  | | | |  |
| **Random effect & residual variance** | | | | | | |
| Study ID | estimate | *I*² | | | | k |
|  | 0.000 | 0.00 | | | | 29 |
| Phylogeny | 0.000 | 0.00 | | | | 21 |
| Species ID | 0.066 | 24.24 | | | | 21 |
| Observation ID | 0.180 | 66.19 | | | | 85 |
|  | *I*²_total_: | 90.60 | | | |  |

**Table S10e. Meta-analytic model estimates explaining variation in lnCVR of among-individual variation due to number of factor included in variance partitioning models** (Model 6e, i.e. differences in among-individual variance between urban and non-urban populations calculated using AD-LMMs). CI stands for confidence intervals, PI for prediction intervals, I² for heterogeneity and k for the number of observations.

| **Fixed Effect** | | | | | | |
| --- | --- | --- | --- | --- | --- | --- |
| Intercept  Number of variable in AD-LMM | estimate | 95% CI | | 95% PI | | k |
|  | -0.565  0.223 | -0.919  0.081 | -0.212  0.366 | -1.498  / | 0.367  / | 85 |
|  |  |  | | | |  |
| **Random effect & residual variance** | | | | | | |
| Study ID | estimate | *I*² | | | | k |
|  | 0.019 | 8.85 | | | | 29 |
| Phylogeny | 0.002 | 0.67 | | | | 21 |
| Species ID | 0.000 | 0.00 | | | | 21 |
| Observation ID | 0.173 | 78.83 | | | | 85 |
|  | *I*²_total_: | 88.35 | | | |  |

**Text S12. Rational behind the additional analysis unravelling why the number of factors added in the AD-LMMs affect the lnCVR of among-individual variation**

When we increase the number of factors in a model, we might explain more variance. This new explained variance could come from the residual variance or from other parts of the model such as ID_ind (the among-individual variance). If increasing the number of factors in our model takes away variance from ID_ind and that this phenomenon is stronger in non-urban populations compared to urban ones, then we might observe that when we increase the number of factors in the model the difference of among-individual variance between urban and non-urban populations (here lnCVR of among-individual variance) increases. Therefore, we decided to test whether the difference of variance explained by all fixed factors between urban and non-urban populations affect lnCVR of among-individual variance. We expect that negative differences in variance explained by the fixed factors (i.e., variance of fixed factor in non-urban populations > variance of fixed factors in urban populations) should increase lnCVR of among-individual variance.

**Table S11. Meta-analytic model estimates explaining variation in lnCVR of among-individual variation including variance differences hold by the fixed factors between urban and non-urban populations** (i.e. differences in among-individual variance between urban and non-urban populations calculated using AD-LMMs). CI stands for confidence intervals, PI for prediction intervals, I² for heterogeneity and k for the number of observations.

| **Fixed Effect** | | | | | | |
| --- | --- | --- | --- | --- | --- | --- |
| Intercept  Diff_variance_urban_nonurban | estimate | 95% CI | | 95% PI | | k |
|  | -0.036  0.000 | -0.177  -0.000 | 0.011  0.000 | -0.976  / | 0.905  / | 85 |
|  |  |  | | | |  |
| **Random effect & residual variance** | | | | | | |
| Study ID | estimate | *I*² | | | | k |
|  | 0.000 | 16.33 | | | | 29 |
| Phylogeny | 0.000 | 0.00 | | | | 21 |
| Species ID | 0.041 | 0.00 | | | | 21 |
| Observation ID | 0.184 | 73.47 | | | | 85 |
|  | *I*²_total_: | 89.80 | | | |  |

**Table S12a. Meta-analytic model estimates explaining variation in lnCVR of within-individual variation due to provenance of data** (Model 7a, i.e. differences in within-individual variance between urban and non-urban populations calculated using AD-LMMs). CI stands for confidence intervals, PI for prediction intervals*,* *I*² for heterogeneity and k for the number of observations.

| **Fixed Effect** | | | | | | |
| --- | --- | --- | --- | --- | --- | --- |
| Both  Field  Lab | estimate | 95% CI | | 95% PI | | k |
|  | -0.283  -0.099  -0.037 | -0.840  -0.298  -0.151 | 0.274  0.100  0.077 | -1.217  -0.875  -0.795 | 0.651  0.677  0.722 | 2  17  66 |
|  |  |  | | | |  |
| **Random effect & residual variance** | | | | | | |
| Study ID | estimate | *I*² | | | | k |
|  | 0.000 | 0.00 | | | | 29 |
| Phylogeny | 0.000 | 0.00 | | | | 21 |
| Species ID | 0.010 | 6.49 | | | | 21 |
| Observation ID | 0.136 | 86.16 | | | | 85 |
|  | *I*²_total_: | 92.65 | | | |  |

**Table S12b. Meta-analytic model estimates explaining variation in lnCVR of within-individual variation due to interval between measurements corrected for lifespan** (Model 7b, i.e. differences in within-individual variance between urban and non-urban populations calculated using AD-LMMs). CI stands for confidence interval, PI for prediction intervals, *I*² for heterogeneity and k for the number of observations.

| **Fixed Effect** | | | | | | |
| --- | --- | --- | --- | --- | --- | --- |
| Short  Very short | estimate | 95% CI | | 95% PI | | k |
|  | -0.157  -0.021 | -0.341  -0.132 | 0.028  0.090 | -0.917  -0.766 | 0.604  0.725 | 21  64 |
|  |  |  | | | |  |
| **Random effect & residual variance** | | | | | | |
| Study ID | estimate | *I*² | | | | k |
|  | 0.000 | 0.00 | | | | 29 |
| Phylogeny | 0.000 | 0.00 | | | | 21 |
| Species ID | 0.007 | 4.54 | | | | 21 |
| Observation ID | 0.135 | 87.88 | | | | 85 |
|  | *I*²_total_: | 92.42 | | | |  |

**Table S12c. Meta-analytic model estimates explaining variation in lnCVR of within-individual variation due to class age** (Model 7c, i.e. differences in within-individual variance between urban and non-urban populations calculated using AD-LMMs). CI stands for confidence interval, PI for prediction intervals, *I*² for heterogeneity and k for the number of observations.

| **Fixed Effect** | | | | | | |
| --- | --- | --- | --- | --- | --- | --- |
| Adult  Juvenile | estimate | 95% CI | | 95% PI | | k |
|  | -0.059  -0.061 | -0.169  -0.301 | 0.050  0.179 | -0.816  -0.847 | 0.698  0.726 | 73  12 |
|  |  |  | | | |  |
| **Random effect & residual variance** | | | | | | |
| Study ID | estimate | *I*² | | | | k |
|  | 0.000 | 0.00 | | | | 29 |
| Phylogeny | 0.000 | 0.00 | | | | 21 |
| Species ID | 0.012 | 7.44 | | | | 21 |
| Observation ID | 0.134 | 85.20 | | | | 85 |
|  | *I*²_total_: | 92.64 | | | |  |

**Table S12d. Meta-analytic model estimates explaining variation in lnCVR of within-individual variation due to taxa** (Model 7d, i.e. differences in within-individual variance between urban and non-urban populations calculated using AD-LMMs). CI stands for confidence intervals, PI for prediction intervals, *I*² for heterogeneity and k for the number of observations.

| **Fixed Effect** | | | | | | |
| --- | --- | --- | --- | --- | --- | --- |
| Amphibian  Bird  Insect  Mammal  Reptile | estimate | 95% CI | | 95% PI | | k |
|  | 0.018  -0.069  0.085  -0.151  -0.079 | -0.549  -0.206  -0.185  -0.386  -0.530 | 0.585  0.068  0.354  0.084  0.373 | -0.928  -0.839  -0.720  -0.944  -0.961 | 0.964  0.701  0.889  0.643  0.804 | 2  52  14  13  4 |
|  |  |  | | | |  |
| **Random effect & residual variance** | | | | | | |
| Study ID | estimate | *I*² | | | | k |
|  | 0.000 | 0.00 | | | | 29 |
| Phylogeny | 0.000 | 0.00 | | | | 21 |
| Species ID | 0.013 | 8.02 | | | | 21 |
| Observation ID | 0.137 | 84.78 | | | | 85 |
|  | *I*²_total_: | 92.80 | | | |  |

**Table S12e. Meta-analytic model estimates explaining variation in lnCVR of within-individual variation number of factor included in variance partitioning models** (Model 7e, i.e. differences in within-individual variance between urban and non-urban populations calculated using AD-LMMs). CI stands for confidence intervals, PI for prediction intervals, *I*² for heterogeneity and k for the number of observations.

| **Fixed Effect** | | | | | | |
| --- | --- | --- | --- | --- | --- | --- |
| Intercept  Number of variable in AD-LMM | estimate | 95% CI | | 95% PI | | k |
|  | -0.092  0.014 | -0.396  -0.108 | 0.212  0.135 | -0.901  / | 0.717  / | 85 |
|  |  |  | | | |  |
| **Random effect & residual variance** | | | | | | |
| Study ID | estimate | *I*² | | | | k |
|  | 0.000 | 0.00 | | | | 29 |
| Phylogeny | 0.000 | 0.00 | | | | 21 |
| Species ID | 0.013 | 8.34 | | | | 21 |
| Observation ID | 0.133 | 84.31 | | | | 85 |
|  | *I*²_total_: | 92.65 | | | |  |

Sensitivity analysis

IOM-LMM versus AD-LMM

**Text S13. Result of the sensitivity analysis contrasting the use of IO-LMMs or AD-LMMs methods for variance estimation**

For among-individual variation, like in the main analysis, we found that the coefficient of among-individual phenotypic variation in urban populations did not differ from non-urban ones whether we perform the analysis on the overall dataset (Table S13a for comparisons) or per phenotypic trait (Table S13b for comparisons). For within-individual variation, like in the main analysis, we found that non-urban populations tended to have on average 6.7% higher coefficient of within-individual phenotypic variation than urban populations but again 95% confidence interval overlapped zero (Model 10: lnCVR estimate [95% CI] = -0.067 [-0.177, 0.044]; Table S14a for comparisons). We also confirmed that the observed trend was most likely due to a pattern emerging from behavioural traits (Model 11: lnCVR behaviour estimate [95% CI] = -0.080 [-0.219, 0.059]; cognition estimate = 0.010 [-0.249, 0.269]; morphology estimate = -0.040 [-0.303, 0.224]; physiology estimate = -0.229 [-0.688, 0.231], Table S14b for comparisons). Analysis on the subset of behavioural traits did not reveal a pattern of an influential behaviour driving this potential effect (Model 12: lnCVR activity/exploration estimate = -0.039 [-0.234, 0.157]; aggression estimate = -0.040 [-0.371, 0.290]; boldness estimate [95% CI] = -0.090 [-0.308, 0.128], Table S14c for comparisons).

**Table S13. Meta-analytic model estimates explaining variation in lnCVR of among-individual variation comparing variance partitioning done via IOM-LMMs with variance partitioning done via AD-LMMs.** a) Comparison between model 8 and model 1. b) Comparison between model 9 and model 2. CI stands for confidence intervals, PI for prediction intervals, I² for heterogeneity and k for the number of observations.

|  | **IOM-LMM** | | | **AD-LMM** | | |
| --- | --- | --- | --- | --- | --- | --- |
| a) **Intercept** *model 8* *model 1* | | | | | | |
| Intercept | estimate | 95% CI [PI] | | estimate | 95% CI [PI] | |
|  | **0.007** | -0.149, 0.135 | [-1.007, 0.993] | **-0.031** | -0.174, 0.112 | [-0.972, 0.910] |
|  |  |  |  |  |  |  |
| **Random effect** | estimate | *I*² | k | estimate | *I*² | k |
| Study ID  Phylogeny  Species ID  Observation_ID | **0.000**  **0.000**  **0.031**  **0.225** | 0.00  0.00  11.61  85.21 | 27  20  20  79 | **0.000**  **0.000**  **0.045**  **0.180** | 0.00  0.00  17.95  71.86 | 29  21  21  85 |
| b) **Type of trait** *model 9* *model 2* | | | | | | |
|  | estimate | 95% CI [PI] | | estimate | 95% CI [PI] | |
| Behaviour  Cognition  Morphology  Physiology | **-0.032**  **-0.315**  **0.069**  **0.495** | -0.233, 0.170  -0.672, 0.042  -0.294, 0.431  -0.093, 1.082 | [-1.027, 0.964]  [-1.353, 0.723]  [-0.971, 1.108]  [-0.643, 1.633] | **-0.044**  **-0.210**  **-0.146**  **0.444** | -0.217, 0.129  -0.554, 0.134  -0.446, 0.155  0.019, 0.869 | [-0.985, 0.898]  [-1.197, 0.777]  [-1.119, 0.827]  [-0.575, 1.462] |
|  |  |  |  |  |  |  |
| **Random effect** | estimate | *I*² | k | estimate | *I*² | k |
| Study ID  Phylogeny  Species ID  Observation_ID | **0.000**  **0.000**  **0.019**  **0.228** | 0.00  0.00  7.42  89.30 | 27  20  20 79 | **0.048**  **0.000**  **0.015**  **0.160** | 19.40  0.00  5.96  68.66 | 29  21  21  85 |

**Table S14. Meta-analytic model estimates explaining variation in lnCVR of within-individual variation comparing variance partitioning done via IOM-LMMs with variance partitioning done via AD-LMMs.** a) Comparison between model 10 and model 3. b) Comparison between model 11 and model 4. c) Comparison between model 12 and model 5. CI stands for confidence intervals, PI for prediction intervals, I² for heterogeneity and k for the number of observations.

|  | **IOM-LMM** | | | | **AD-LMM** | | |
| --- | --- | --- | --- | --- | --- | --- | --- |
| a) **Intercept** *model 10* *model 3* | | | | | | | |
| Intercept | estimate | 95% CI [PI] | | estimate | | 95% CI [PI] | |
|  | **-0.067** | -0.177, 0.044 | [-0.774, 0.641] | **-0.059** | | -0.159, 0.041 | [-0.808, 0.690] |
|  |  |  |  |  | |  |  |
| **Random effect** | estimate | *I*² | k | estimate | | *I*² | k |
| Study ID  Phylogeny  Species ID  Observation_ID | **0.000**  **0.000**  **0.025**  **0.103** | 0.00  0.00  17.85  74.37 | 27  20  20  79 | **0.000**  **0.000**  **0.011**  **0.133** | | 0.00  0.00  6.86  85.67 | 29  21  21  85 |
| b) **Type of trait** *model 11* *model 4* | | | | | | | |
|  | estimate | 95% CI [PI] | | estimate | | 95% CI [PI] | |
| Behaviour  Cognition  Morphology  Physiology | **-0.080**  **0.010**  **-0.040**  **-0.229** | -0.219, 0.059  -0.249, 0.269  -0.303, 0.224  -0.688, 0.231 | [-0.819, 0.659]  [-0.761, 0.781]  [-0.812, 0.733]  [-1.088, 0.630] | **-0.067**  **0.045**  **-0.017**  **-0.232** | | -0.192, 0.057  -0.226, 0.316  -0.268, 0.233  -0.564, 0.100 | [-0.828, 0.694]  [-0.753, 0.844]  [-0.809, 0.774]  [-1.053, 0.589] |
|  |  |  |  |  | |  |  |
| **Random effect** | estimate | *I*² | k | estimate | | *I*² | k |
| Study ID  Phylogeny  Species ID  Observation_ID | **0.000**  **0.000**  **0.036**  **0.102** | 0.00  0.00  23.97  68.77 | 27  20  20 79 | **0.000**  **0.000**  **0.013**  **0.134** | | 0.00  0.00  8.14  84.53 | 29  21  21  85 |
| c) **Type of behaviour** *model 12* *model 5* | | | | | | | |
|  | estimate | 95% CI [PI] | | estimate | | 95% CI [PI] | |
| Activity/exploration  Aggression  Boldness | **-0.039**  **-0.040**  **-0.090** | -0.234, 0.157  -0.371, 0.290  -0.308, 0.128 | [-0.811, 0.734]  [-0.858, 0.777]  [-0.869, 0.689] | **-0.068**  **-0.044**  **-0.059** | | -0.250, 0.113  -0.366, 0.278  -0.293, 0.176 | [-0.866, 0.730]  [-0.885, 0.797]  [-0.870, 0.753] |
|  |  |  |  |  | |  |  |
| **Random effect** | estimate | *I*² | k | estimate | | *I*² | k |
| Study ID  Phylogeny  Species ID  Observation_ID | **0.007**  **0.000**  **0.034**  **0.099** | 4.61  0.00  25.67  63.35 | 17  14  14 52 | **0.000**  **0.000**  **0.022**  **0.136** | | 0.00  0.00  12.90  81.04 | 16  13  13  52 |

Bird versus all other taxa combined

**Table S15. Meta-analytic model estimates explaining variation in lnCVR of a) among-individual and b) within-individual variation comparing effect size coming only from bird taxa with effect size coming only from other taxa than bird.** Variance estimates from AD-LMMs. CI stands for confidence interval, PI for prediction intervals, I² for heterogeneity and k for the number of observations.

|  | **Only bird** | | | **All other taxa combined** | | |
| --- | --- | --- | --- | --- | --- | --- |
| a) **Among-individual** *model 13* *model 15* | | | | | | |
| Intercept | estimate | 95% CI [PI] | | estimate | 95% CI [PI] | |
|  | **-0.029** | -0.299, 0.240 | [-1.166, 1.107] | **-0.043** | -0.215, 0.128 | [-0.818, 0.732] |
|  |  |  |  |  |  |  |
| **Random effect** | estimate | *I*² | k | estimate | *I*² | k |
| Study ID  Phylogeny  Species ID  Observation_ID | **0.000**  **0.000**  **0.139**  **0.179** | 0.00  0.00  39.80  51.31 | 17  11  11  52 | **0.000**  **0.008**  **0.000**  **0.141** | 0.00  4.53  0.00  83.51 | 12  10  10  33 |
| b) **Within-individual** *model 14* *model 16* | | | | | | |
| Intercept | estimate | 95% CI [PI] | | estimate | 95% CI [PI] | |
|  | **-0.068** | -0.199, 0.063 | [-0.863, 0.727] | **-0.056** | -0.211,0.101 | [-0.765, 0.654] |
|  |  |  |  |  |  |  |
| **Random effect** | estimate | *I*² | k | estimate | *I*² | k |
| Study ID  Phylogeny  Species ID  Observation_ID | **0.000**  **0.000**  **0.007**  **0.153** | 0.00  0.00  3.86  88.25 | 17  11  11 52 | **0.000**  **0.007**  **0.017**  **0.107** | 0.00  0.50  12.88  79.51 | 12  10  10  33 |

Single study effect

**Table S16. Single study effect model estimates of overall lnCVR of a) among-individual and b) within-individual variation.** Variance estimated from AD-LMMs. CI stands for confidence interval.

| **Single study effect** | | | |
| --- | --- | --- | --- |
| a) **Among-individual** | | | |
| ID_study removed  1  2  3  4  5  6  7  8  9  10  11  12  13  14  15  16  17  18  19  20  21  22  23  24  25  26  27  28  29  30  31 | estimate | 95% CI | |
|  | **-0.047**  **-0.017**  **-0.041**  **-0.038**  **-0.049**  **-0.042**  **-0.025**  **-0.039**  **-0.039**  **-0.041**  **-0.001**  **-0.032**  **-0.002**  **-0.020**  **-0.033**  **-0.031**  **-0.030**  **-0.031**  **-0.032**  **-0.052**  **-0.019**  **-0.033**  **-0.034**  **-0.032**  **-0.027**  **-0.033**  **-0.030**  **-0.016**  **-0.052**  **-0.029**  **-0.024** | -0.192  -0.166  -0.188  -0.186  -0.194  -0.186  -0.170  -0.188  -0.192  -0.186  -0.126  -0.181  -0.129  -0.177  -0.177  -0.174  -0.180  -0.174  -0.175  -0.194  -0.167  -0.180  -0.177  -0.180  -0.171  -0.179  -0.173  -0.179  -0.200  -0.178  -0.162 | 0.098  0.131  0.108  0.111  0.095  0.102  0.119  0.111  0.011  0.103  0.128  0.118  0.126  0.106  0.110  0.112  0.121  0.112  0.112  0.090  0.131  0.112  0.109  0.115  0.117  0.112  0.114  0.147  0.095  0.120  0.113 |
| b) **Within-individual** | | | |
| ID_study removed  1  2  3  4  5  6  7  8  9  10  11  12  13  14  15  16  17  18  19  20  21  22  23  24  25  26  27  28  29  30  31 | estimate | 95% CI | |
|  | **-0.058**  **-0.051**  **-0.068**  **-0.056**  **-0.046**  **-0.067**  **-0.063**  **-0.069**  **-0.064**  **-0.067**  **-0.063**  **-0.053**  **-0.061**  **-0.043**  **-0.060**  **-0.059**  **-0.062**  **-0.059**  **-0.060**  **-0.041**  **-0.059**  **-0.062**  **-0.066**  **-0.060**  **-0.070**  **-0.060**  **-0.057**  **-0.048**  **-0.051**  **-0.058**  **-0.049** | **-0.162**  **-0.155**  **-0.173**  **-0.163**  **-0.146**  **-0.168**  **-0.166**  **-0.172**  **-0.172**  **-0.168**  **-0.169**  **-0.155**  **-0.164**  **-0.137**  **-0.160**  **-0.159**  **-0.166**  **-0.159**  **-0.162**  **-0.140**  **-0.164**  **-0.162**  **-0.162**  **-0.161**  **-0.159**  **-0.159**  **-0.162**  **-0.144**  **-0.154**  **-0.161**  **-0.144** | **0.046**  **0.052**  **0.036**  **0.051**  **0.054**  **0.034**  **0.040**  **0.034**  **0.045**  **0.034**  **0.042**  **0.049**  **0.042**  **0.050**  **0.040**  **0.041**  **0.042**  **0.041**  **0.041**  **0.057**  **0.046**  **0.039**  **0.030**  **0.041**  **0.020**  **0.039**  **0.048**  **0.049**  **0.051**  **0.046**  **0.046** |

**Figure S4. Single study effect on lnCVR estimates of among-individual variation.** The dashed red lines show the 95% confidence interval for the overall effect size in the original model. The blank dots (grey circle) show the effect sizes left out of that specific model. The thick black line shows the 95% confidence intervals. The thin black line shows the 95% prediction intervals. k is the number of observations supplemented with the number of studies between brackets.

**Figure S5. Single study effect on lnCVR estimates of within-individual variation.** The dashed red lines show the 95% confidence interval for the overall effect size in the original model. The thick black line shows the 95% confidence intervals. The thin black line shows the 95% prediction intervals. The blank dots (grey circle) show the effect sizes left out of that specific model. k is the number of observations supplemented with the number of studies between brackets.

Publication bias

**Text S14. Result of the publication bias on overall lnCVR estimate of among- and within-individual (i.e. small-study and decline effects)**

We did not find evidence for the existence of small-study or decline effects for lnCVR of among- and within-individual variation estimates (Model 19: *among-individual variation* slope estimate for the square-root of the inverse of the effective sample size [95% CI] = -0.097 [-1.616, 1.423], R²_marginal_ = 0.0003, Table S17a; Model 20: *within-individual variation* slope estimate for the square-root of the inverse of the effective sample size [95% CI] = -0.346 [-1.444, 0.752], R²_marginal_ = 0.0063, Table S17b; Model 21: *among-individual variation* slope estimate for year of publication [95% CI] = 0.032 [-0.020, 0.084], R²_marginal_ = 0.023, Table S18a); Model 22: *within-individual variation* slope estimate for year of publication [95% CI] = 0.007 [-0.048, 0.034], R²_marginal_ = 0.002, Table S18b for comparisons). According to Nakagawa *et al.* (2022), our results did not seem to suffer from publication bias.

**Table S17. Meta-analytic model estimates explaining variation in lnCVR due to small-study effect of a) among-individual and b) within-individual variation.** Variance estimated from AD-LMMs. CI stands for confidence interval, I² for heterogeneity and k for the number of observations, R² is the marginal coefficient of determination.

| **Small-study effect** | | | | | |
| --- | --- | --- | --- | --- | --- |
| a) **Among-individual** | | | | | |
| Intercept  sqrt_inv_eff_ss | estimate | 95% CI | | | *R*² |
|  | 0.003  -0.097 | -0.494  -1.616 | 0.493  1.423 | | **0.0003** |
|  |  |  | |  | |
| **Random effect** | estimate | *I*² | | k | |
| Study ID  Phylogeny  Species ID  Observation_ID | 0.000  0.000  0.049  0.181 | 0.00  0.00  19.03  70.97 | | 29  21  21  85 | |
| b) **Within-individual** | | | | | |
| Intercept  sqrt_inv_eff_ss | estimate | 95% CI | | | *R*² |
|  | -0.049  -0.346 | -0.304  -1.444 | 0.403  0.752 | | **0.0063** |
|  |  |  | |  | |
| **Random effect** | estimate | *I*² | | k | |
| Study ID  Phylogeny  Species ID  Observation_ID | 0.00  0.00  0.008  0.135 | 0.00  0.00  5.17  87.37 | | 29  21  21 85 | |

**Table S18. Meta-analytic model estimates explaining variation in lnCVR due to decline effect of a) among-individual and b) within-individual variation.** Variance estimated from AD-LMMs. CI stands for confidence interval, I² for heterogeneity and k for the number of observations, R² is the marginal coefficient of determination.

| **Decline effect** | | | | | |
| --- | --- | --- | --- | --- | --- |
| a) **Among-individual** | | | | | |
| Intercept  pub_year_c | estimate | 95% CI | | | *R*² |
|  | -0.041  0.032 | -0.185  -0.020 | 0.103  0.084 | | **0.023** |
|  |  |  | |  | |
| **Random effect** | estimate | *I*² | | k | |
| Study ID  Phylogeny  Species ID  Observation_ID | 0.000  0.000  0.045  0.179 | 0.00  0.00  18.03  71.73 | | 29  21  21  85 | |
| b) **Within-individual** | | | | | |
| Intercept  pub_year_c | estimate | 95% CI | | | *R*² |
|  | -0.058  -0.007 | -0.160  -0.048 | 0.044  0.034 | | **0.002** |
|  |  |  | |  | |
| **Random effect** | estimate | *I*² | | k | |
| Study ID  Phylogeny  Species ID  Observation_ID | 0.00  0.00  0.011  0.135 | 0.00  0.00  7.26  85.37 | | 29  21  21 85 | |

Appendix – PRISMA EcoEvo checklist

**Table S19. PRISMA EcoEvo checklist from O’Dea et al. (2021).** Summarise the main items necessary for a complete meta-analysis. In total, we reported 73 items as 19 were non-applicable. Overall reporting score is 97% as 2 items were not reported. As the published version might change the original format of our document, we advise to contact the main author to obtain the adequate word document.

| **Checklist item** | **Item score** | **Sub-item number** | **Sub-item** | **Reported by authors?** | **Notes** |
| --- | --- | --- | --- | --- | --- |
| **Title and abstract** | **100%** | 1.1 | Identify the review as a systematic review, meta-analysis, or both | Yes | Line: 2, 60 |
|  |  | 1.2 | Summarise the aims and scope of the review | Yes | Line: 60-62 |
|  |  | 1.3 | Describe the data set | Yes | Line: 60-62 |
|  |  | 1.4 | State the results of the primary outcome | Yes | Line: 62-64 |
|  |  | 1.5 | State conclusions | Yes | Line: 64-68 |
|  |  | 1.6 | State limitations | Yes | Reported in introduction (L157-166) as a disclaimer paragraph due to lack of space |
| **Aims and questions** | **100%** | 2.1 | Provide a rationale for the review | Yes | Line: 95-100, 114-117 |
|  |  | 2.2 | Reference any previous reviews or meta-analyses on the topic | Yes | Line: 140-143 |
|  |  | 2.3 | State the aims and scope of the review (including its generality) | Yes | Line: 144-152 |
|  |  | 2.4 | State the primary questions the review addresses (e.g. which moderators were tested) | Yes | Line: 152-156 |
|  |  | 2.5 | Describe whether effect sizes were derived from experimental and/or observational comparisons | Yes | Line: 151-152 |
| **Review registration** | **0%** | 3.1 | Register review aims, hypotheses (if applicable), and methods in a time-stamped and publicly accessible archive and provide a link to the registration in the methods section of the manuscript. Ideally registration occurs before the search, but it can be done at any stage before data analysis. | Not applicable | The study was not registered |
|  |  | 3.2 | Describe deviations from the registered aims and methods | Not applicable | The study was not registered |
|  |  | 3.3 | Justify deviations from the registered aims and methods | Not applicable | The study was not registered |
| **Eligibility criteria** | **50%** | 4.1 | Report the specific criteria used for including or excluding studies when screening titles and/or abstracts, and full texts, according to the aims of the systematic review (e.g. study design, taxa, data availability) | Yes | Table S1, Figure S1 |
|  |  | 4.2 | Justify criteria, if necessary (i.e. not obvious from aims and scope) | Not applicable | Not reported due to the obvious aim and scope of the meta-analysis |
| **Finding studies** | **100%** | 5.1 | Define the type of search (e.g. comprehensive search, representative sample) | Yes | Line: 174-175 |
|  |  | 5.2 | State what sources of information were sought (e.g. published and unpublished studies, personal communications) | Yes | Line: 176-178 |
|  |  | 5.3 | Include, for each database searched, the exact search strings used, with keyword combinations and Boolean operators | Yes | Table S1 |
|  |  | 5.4 | Provide enough information to repeat the equivalent search (if possible), including the timespan covered (start and end dates) | Yes | Table S1, Text S4 |
| **Study selection** | **100%** | 6.1 | Describe how studies were selected for inclusion at each stage of the screening process (e.g. use of decision trees, screening software) | Yes | Figure S1 |
|  |  | 6.2 | Report the number of people involved and how they contributed (e.g. independent parallel screening) | Yes | Line: 190-192, 214, see ‘Acknowledgement’ |
| **Data collection process** | **83%** | 7.1 | Describe where in the reports data were collected from (e.g. text or figures) | Yes | Line: 197-199 |
|  |  | 7.2 | Describe how data were collected (e.g. software used to digitize figures, external data sources) | Yes | Line: 197-199 |
|  |  | 7.3 | Describe moderator variables that were constructed from collected data (e.g. number of generations calculated from years and average generation time) | Yes | See ‘Data categorisation’ (Line 207-243) |
|  |  | 7.4 | Report how missing or ambiguous information was dealt with during data collection (e.g. authors of original studies were contacted for missing descriptive statistics, and/or effect sizes were calculated from test statistics) | Yes | Line: 198, Text S5, Text S6, 222-224 |
|  |  | 7.5 | Report who collected data | Yes | Line: 190-191 |
|  |  | 7.6 | State the number of extractions that were checked for accuracy by co-authors | Not applicable | Data extraction was performed only by the first author |
| **Data items** | **100%** | 8.1 | Describe the key data sought from each study | Yes | Line: 197-198, See ‘Data categorisation’ (line 207-243) |
|  |  | 8.2 | Describe items that do not appear in the main results, or which could not be extracted due to insufficient information | Yes | Line: 241-243 |
|  |  | 8.3 | Describe main assumptions or simplifications that were made (e.g. categorising both 'length' and 'mass' as 'morphology') | Yes | Line: 212-214 (relabelling of traits), 217-221 (assumption about macro-environmental change between urban and non-urban populations), 225-236 (approximation of inter-test interval classification and lifespan), 247-249 (gaussian approximation for count data), 265-270 (correction for population plasticity and sex-corrected assessment), 278-280 (how among- and within-individual variation are approximated) |
|  |  | 8.4 | Describe the type of replication unit (e.g. individuals, broods, study sites) | Yes | Line: 245-246 |
| **Assessment of individual study quality** | **0%** | 9.1 | Describe whether the quality of studies included in the systematic review or meta-analysis was assessed (e.g. blinded data collection, reporting quality, experimental versus observational) | Not applicable | The quality of the studies was not assessed |
|  |  | 9.2 | Describe how information about study quality was incorporated into analyses (e.g. meta-regression and/or sensitivity analysis) | Not applicable | The quality of the studies was not assessed |
| **Effect size measures** | **66%** | 10.1 | Describe effect size(s) used | Yes | Line: 286-288 |
|  |  | 10.2 | Provide a reference to the equation of each calculated effect size (e.g. standardised mean difference, log response ratio) and (if applicable) its sampling variance | Yes | Line: 292-296 |
|  |  | 10.3 | If no reference exists, derive the equations for each effect size and state the assumed sampling distribution(s) | Not applicable | The reference exists and was reported (see item 10.2) |
| **Missing data** | **0%** | 11.1 | Describe any steps taken to deal with missing data during analysis (e.g. imputation, complete case, subset analysis) | Not applicable | Missing data was not used |
|  |  | 11.2 | Justify the decisions made to deal with missing data | Not applicable | Missing data was not used |
| **Meta-analytic model description** | **0%** | 12.1 | Describe the models used for synthesis of effect sizes | Yes | Line: 334-351 |
|  |  | 12.2 | The most common approach in ecology and evolution will be a random-effects model, often with a hierarchical/multilevel structure. If other types of models are chosen (e.g. common/fixed effects model, unweighted model), provide justification for this choice | Yes | Line: 336, 345. No-intercept model allows a direct estimation of each factor level. |
| **Software** | **80%** | 13.1 | Describe the statistical platform used for inference (e.g. R) | Yes | Line: 360-361 |
|  |  | 13.2 | Describe the packages used to run models | Yes | Line: 254, 328, 350, 356, 359, 380 |
|  |  | 13.3 | Describe the functions used to run models | Yes | See item 13.2 |
|  |  | 13.4 | Describe any arguments that differed from the default settings | Not applicable | Reported in the R script |
|  |  | 13.5 | Describe the version numbers of all software used | Yes | See items 13.1, 13.2, and R script |
| **Non-independence** | **100%** | 14.1 | Describe the types of non-independence encountered (e.g. phylogenetic, spatial, multiple measurements over time) | Yes | Line: 303-305, 327-332 |
|  |  | 14.2 | Describe how non-independence has been handled | Yes | See item 14.1 |
|  |  | 14.3 | Justify decisions made | Yes | See item 14.1 |
| **Meta-regression and model selection** | **66%** | 15.1 | Provide a rationale for the inclusion of moderators (covariates) that were evaluated in meta-regression models | Yes | See ‘Data categorisation’ (line 207-243) |
|  |  | 15.2 | Justify the number of parameters estimated in models, in relation to the number of effect sizes and studies (e.g. interaction terms were not included due to insufficient sample sizes) | Yes | Line: 241-243, 350-351 |
|  |  | 15.3 | Describe any process of model selection | Not applicable | No model selection performed |
| **Publication bias and sensitivity analysis** | **100%** | 16.1 | Describe assessments of the risk of bias due to missing results (e.g. publication, time-lag, and taxonomic biases) | Yes | Line: 366-368, 372-380 |
|  |  | 16.2 | Describe any steps taken to investigate the effects of such biases (if present) | Yes | See item 16.1 |
|  |  | 16.3 | Describe any other analyses of robustness of the results, e.g. due to effect size choice, weighting or analytical model assumptions, inclusion or exclusion of subsets of the data, or the inclusion of alternative moderator variables in meta-regressions | Yes | See item 16.1 |
| **Clarification of post hoc analyses** | **0%** | 17.1 | When hypotheses were formulated after data analysis, this should be acknowledged. | Not applicable | Hypotheses were made a priori of data collection |
| **Metadata, data, and code** | **100%** | 18.1 | Share metadata (i.e. data descriptions) | Yes | See Dryad depository |
|  |  | 18.2 | Share data required to reproduce the results presented in the manuscript | Yes | See Dryad depository |
|  |  | 18.3 | Share additional data, including information that was not presented in the manuscript (e.g. raw data used to calculate effect sizes, descriptions of where data were located in papers) | Yes | See Dryad depository |
|  |  | 18.4 | Share analysis scripts (or, if a software package with graphical user interface (GUI) was used, then describe full model specification and fully specify choices) | Yes | See Dryad depository |
| **Results of study selection process** | **100%** | 19.1 | Report the number of studies screened | Yes | Line: 180, Figure S1 |
|  |  | 19.2 | Report the number of studies excluded at each stage of screening | Yes | Line: 180-186, Figure S1 |
|  |  | 19.3 | Report brief reasons for exclusion from the full text stage | Yes | See Excel table ‘Summary screening process from 2nd screening’ in Dryad depository |
|  |  | 19.4 | Present a Preferred Reporting Items for Systematic Reviews and Meta-Analyses (PRISMA)-like flowchart (www.prisma-statement.org). | Yes | See this present document |
| **Sample sizes and study characteristics** | **60%** | 20.1 | Report the number of studies and effect sizes for data included in meta-analyses | Yes | Line: 311-312, Text S10 |
|  |  | 20.2 | Report the number of studies and effect sizes for subsets of data included in meta-regressions | Yes | See specific main result tables or supplementary tables according to specific model of interest |
|  |  | 20.3 | Provide a summary of key characteristics for reported outcomes (either in text or figures; e.g. one quarter of effect sizes reported for vertebrates and the rest invertebrates) | Yes | Line: 366, Figure 1 |
|  |  | 20.4 | Provide a summary of limitations of included moderators (e.g. collinearity and overlap between moderators) | No | No summary reported for a matter a manuscript length, but assumptions are explicitly stated, see item 15.1 |
|  |  | 20.5 | Provide a summary of characteristics related to individual study quality (risk of bias) | Not applicable | Study quality was not assessed |
| **Meta-analysis** | **67%** | 21.1 | Provide a quantitative synthesis of results across studies, including estimates for the mean effect size, with confidence/credible intervals | Yes | See ‘Results’ section |
| **Heterogeneity** |  | 22.1 | Report indicators of heterogeneity in the estimated effect (e.g. I2, tau2 and other variance components) | Yes | See ‘Results’ section |
| **Meta-regression** |  | 23.1 | Provide estimates of meta-regression slopes (i.e. regression coefficients) and confidence/credible intervals | Yes | Line: 415-417 (Table S10e) |
|  |  | 23.2 | Include estimates and confidence/credible intervals for all moderator variables that were assessed (i.e. complete reporting) | Yes | Line: 415-428 + Table S10a-e, TableS12a-e |
|  |  | 23.3 | Report interactions, if they were included | Not applicable | No models with interactions |
|  |  | 23.4 | Describe outcomes from model selection, if done (e.g. R2 and AIC) | Not applicable | No model selection |
| **Outcomes of publication bias and sensitivity analysis** | **100%** | 24.1 | Provide results for the assessments of the risks of bias (e.g. Egger's regression, funnel plots) | Yes | Table S17 and S18 |
|  |  | 24.2 | Provide results for the robustness of the review's results (e.g. subgroup analyses, meta-regression of study quality, results from alternative methods of analysis, and temporal trends) | Yes | Line: 429-456 |
| **Discussion** | **83%** | 25.1 | Summarise the main findings in terms of the magnitude of effect | Yes | Line: 461-465 |
|  |  | 25.2 | Summarise the main findings in terms of the precision of effects (e.g. size of confidence intervals, statistical significance) | Yes | Line: 461-465 |
|  |  | 25.3 | Summarise the main findings in terms of their heterogeneity | No | Not the main results |
|  |  | 25.4 | Summarise the main findings in terms of their biological/practical relevance | Yes | Line: 474-476 (within-individual variation), 564-565 and 581-584 (among-individual variation) |
|  |  | 25.5 | Compare results with previous reviews on the topic, if available | Yes | Line: 488-490 and 526-530 (within-individual variation), 566 (among-individual variation) |
|  |  | 25.6 | Consider limitations and their influence on the generality of conclusions, such as gaps in the available evidence (e.g. taxonomic and geographical research biases) | Yes | Line: 477-481 (higher environmental heterogeneity in urban areas), 483-484 (trait relevance), 515-520 and 530-533 (approximation of individual plasticity and predictability), 560-563 (competition effects on among-individual variation) |
| **Contributions and funding** | **100%** | 26.1 | Provide names, affiliations, and funding sources of all co-authors | Yes | Line: 5-8, 1005-1009 |
|  |  | 26.2 | List the contributions of each co-author | Yes | Line: 20-25 |
|  |  | 26.3 | Provide contact details for the corresponding author | Yes | Line: 9-10 |
|  |  | 26.4 | Disclose any conflicts of interest | Yes | Line: 1011 |
| **References** | **100%** | 27.1 | Provide a reference list of all studies included in the systematic review or meta-analysis | Yes | Line: 311-319 |
|  |  | 27.2 | List included studies as referenced sources (e.g. rather than listing them in a table or supplement) | Yes | Line: 311-319 |

Appendix - Literature

Bar‐Ziv, M., Sofer, A., Gorovoy, A. & Spiegel, O. (2023). Beyond simple habituation: Anthropogenic habitats influence the escape behaviour of spur‐winged lapwings in response to both human and non‐human threats. *Journal of Animal Ecology*, 92, 417–429.

Carrete, M. & Tella, J.L. (2013). High individual consistency in fear of humans throughout the adult lifespan of rural and urban burrowing owls. *Sci Rep*, 3, 3524.

Carrete, M. & Tella, J.L. (2017). Behavioral Correlations Associated with Fear of Humans Differ between Rural and Urban Burrowing Owls. *Frontiers in Ecology and Evolution*, 5.

Charmantier, A., Demeyrier, V., Lambrechts, M., Perret, S. & Grégoire, A. (2017). Urbanization Is Associated with Divergence in Pace-of-Life in Great Tits. *Frontiers in Ecology and Evolution*, 5.

Crawley, J.N. (1985). Exploratory behavior models of anxiety in mice. *Neuroscience & Biobehavioral Reviews*, 9, 37–44.

Davies, S. & Sewall, K.B. (2016). Agonistic urban birds: elevated territorial aggression of urban song sparrows is individually consistent within a breeding period. *Biology Letters*, 12, 20160315.

Dominoni, D.M., Van’t Hof, T.J. & Partecke, J. (2015). Social cues are unlikely to be the single cause for early reproduction in urban European blackbirds (*Turdus merula*). *Physiology & Behavior*, 142, 14–19.

Evans, J., Boudreau, K. & Hyman, J. (2010). Behavioural Syndromes in Urban and Rural Populations of Song Sparrows. *Ethology*.

Fellows, I. (2018). wordcloud: Word Clouds. *R package version 2.6*.

Foo, Y.Z., O’Dea, R.E., Koricheva, J., Nakagawa, S. & Lagisz, M. (2021). A practical guide to question formation, systematic searching and study screening for literature reviews in ecology and evolution. *Methods in Ecology and Evolution*, 12, 1705–1720.

Garitano-Zavala, Á., Calbimonte, R. & Esteve-Herraiz, G. (2022). The Behavioral Responses of the Chiguanco Thrush to Urbanization in a Neotropical City Comes From Preadapted Behavioral Traits. *Front. Ecol. Evol.*, 10.

Greenberg, R. (2003). The Role of Neophobia and Neophilia in the Development of Innovative Behaviour of Birds. In: *Animal Innovation* (eds. Reader, S.M. & Laland, K.N.). Oxford University Press, pp. 175–196.

Greenberg, R. & Mettke-hofmann, C. (2001). Ecological Aspects of Neophobia and neophilia in birds. In: *Current Ornithology, Volume 16* (eds. Nolan, V. & Thompson, C.F.). Springer US, Boston, MA, pp. 119–178.

Griffin, A.S., Guillette, L.M. & Healy, S.D. (2015). Cognition and personality: an analysis of an emerging field. *Trends in Ecology & Evolution*, 30, 207–214.

Grunst, M.L., Rotenberry, J.T. & Grunst, A.S. (2014). Variation in adrenocortical stress physiology and condition metrics within a heterogeneous urban environment in the song sparrow Melospiza melodia. *Journal of Avian Biology*, 45, 574–583.

Hardman, S.I. & Dalesman, S. (2018). Repeatability and degree of territorial aggression differs among urban and rural great tits (Parus major). *Sci Rep*, 8, 5042.

Harten, L., Gonceer, N., Handel, M., Dash, O., Fokidis, H.B. & Yovel, Y. (2021). Urban bat pups take after their mothers and are bolder and faster learners than rural pups. *BMC Biol*, 19, 190.

Heppner, J.J., Krause, J.S. & Ouyang, J.Q. (2023). Urbanization and maternal hormone transfer: Endocrine and morphological phenotypes across ontogenetic stages. *General and Comparative Endocrinology*, 333, 114166.

Huang, P., St.Mary, C.M. & Kimball, R.T. (2020). Habitat urbanization and stress response are primary predictors of personality variation in northern cardinals (Cardinalis cardinalis). *Journal of Urban Ecology*, 6, juaa015.

Kaiser, A., Merckx, T. & Van Dyck, H. (2018). Urbanisation and sex affect the consistency of butterfly personality across metamorphosis. *Behavioral Ecology and Sociobiology*, 72, 1–11.

King, J.A. (1973). The Ecology of Aggressive Behavior. *Annu. Rev. Ecol. Syst.*, 4, 117–138.

Kozlovsky, D.Y., Weissgerber, E.A. & Pravosudov, V.V. (2017). What makes specialized food-caching mountain chickadees successful city slickers? *Proceedings of the Royal Society B: Biological Sciences*, 284, 20162613.

Lang, D. & Chien, G. (2018). wordcloud2: Create Word Cloud by “htmlwidget.” *R package version 0.2.1*.

Lüdecke, D., Ben-Shachar, M., Patil, I., Waggoner, P. & Makowski, D. (2021). performance: An R Package for Assessment, Comparison and Testing of Statistical Models. *JOSS*, 6, 3139.

Mazza, V. & Guenther, A. (2021). City mice and country mice: innovative problem solving in rural and urban noncommensal rodents. *Animal Behaviour*, 172, 197–210.

Mettke‐Hofmann, C., Winkler, H. & Leisler, B. (2002). The Significance of Ecological Factors for Exploration and Neophobia in Parrots. *Ethology*, 108, 249–272.

Meyer, J.S. (1998). Chapter 22 - Behavioral Assessment in Developmental Neurotoxicology: Approaches Involving Unconditioned Behaviors Pharmacologic Challenges in Rodents. In: *Handbook of Developmental Neurotoxicology* (eds. Slikker, W. & Chang, L.W.). Academic Press, San Diego, pp. 403–426.

Miranda, A.C., Schielzeth, H., Sonntag, T. & Partecke, J. (2013). Urbanization and its effects on personality traits: a result of microevolution or phenotypic plasticity? *Glob Change Biol*, 19, 2634–2644.

Møller, A.P. & Tryjanowski, P. (2014). Direction of approach by predators and flight initiation distance of urban and rural populations of birds. *Behavioral Ecology*, 25, 960–966.

Nakagawa, S., Lagisz, M., Jennions, M.D., Koricheva, J., Noble, D.W.A., Parker, T.H., *et al.* (2022). Methods for testing publication bias in ecological and evolutionary meta‐analyses. *Methods Ecol Evol*, 13, 4–21.

O’Dea, R.E., Lagisz, M., Jennions, M.D., Koricheva, J., Noble, D.W.A., Parker, T.H., *et al.* (2021). Preferred reporting items for systematic reviews and meta‐analyses in ecology and evolutionary biology: a PRISMA extension. *Biological Reviews*, 96, 1695–1722.

Ouyang, J.Q., Baldan, D., Munguia, C. & Davies, S. (2019). Genetic inheritance and environment determine endocrine plasticity to urban living. *Proceedings of the Royal Society B: Biological Sciences*, 286, 20191215.

Papp, S., Vincze, E., Preiszner, B., Liker, A. & Bókony, V. (2015). A comparison of problem-solving success between urban and rural house sparrows. *Behav Ecol Sociobiol*, 69, 471–480.

Paré, W.P. & Glavin, G.B. (1993). Animal models of stress in pharmacology. In: *Techniques in the Behavioral and Neural Sciences*. Elsevier, pp. 413–441.

Petit, J. & Dammhahn, M. (2024). Data for: Petit & Dammhahn dataset. [Dataset], DOI: 10.5061/dryad.2rbnzs82d.

Prasher, S., Evans, J.C., Thompson, M.J. & Morand-Ferron, J. (2019). Characterizing innovators: Ecological and individual predictors of problem-solving performance. *PLoS ONE*, 14, e0217464.

R Core Team. (2022). R: a language and environment for statistical computing. *Vienna, Austria: R Foundation for Statistical Computing*.

Réale, D., Reader, S.M., Sol, D., McDougall, P.T. & Dingemanse, N.J. (2007). Integrating animal temperament within ecology and evolution. *Biological Reviews*, 82, 291–318.

Rimbach, R. & Dammhahn, M. (2024). Data for: Rimbach & Dammhahn dataset [Dataset], DOI: 10.5061/dryad.2rbnzs82d.

Scales, J., Hyman, J. & Hughes, M. (2011). Behavioral Syndromes Break Down in Urban Song Sparrow Populations: Behavioral Syndromes Break Down in Urban Song Sparrow Populations. *Ethology*, 117, 887–895.

Schielzeth, H., Dingemanse, N.J., Nakagawa, S., Westneat, D.F., Allegue, H., Teplitsky, C., *et al.* (2020). Robustness of linear mixed-effects models to violations of distributional assumptions. *Methods in Ecology and Evolution*, 11, 1141–1152.

Solaro, C. & Sarasola, J.H. (2019). Urban living predicts behavioural response in a neotropical raptor. *Behavioural Processes*, 169, 103995.

Stansell, H.M., Blumstein, D.T., Yeh, P.J. & Nonacs, P. (2022). Individual variation in tolerance of human activity by urban Dark-eyed Juncos (Junco hyemalis). *The Wilson Journal of Ornithology*, 134, 43–51.

Tabh, J.K.R., Mastromonaco, G.F. & Burness, G. (2022). Stress-induced changes in body surface temperature are repeatable, but do not differ between urban and rural birds. *Oecologia*, 198, 663–677.

Thompson, M.J., Evans, J.C., Parsons, S. & Morand-Ferron, J. (2018). Urbanization and individual differences in exploration and plasticity. *Behavioral Ecology*.

Thompson, M.J. & Morand-Ferron, J. (2019). Food caching in city birds: urbanization and exploration do not predict spatial memory in scatter hoarders. *Anim Cogn*, 22, 743–756.

Tüzün, N., Müller, S., Koch, K. & Stoks, R. (2017). Pesticide-induced changes in personality depend on the urbanization level. *Animal Behaviour*, 134, 45–55.

Uchida, K., Shimamoto, T., Yanagawa, H. & Koizumi, I. (2020). Comparison of multiple behavioral traits between urban and rural squirrels. *Urban Ecosyst*, 23, 745–754.

Williams, S.B. (1942). Reversal learning after two degrees of training. *Journal of Comparative Psychology*, 34, 353–360.

Zuur, A.F., Ieno, E.N., Walker, N.J., Saveliev, A.A. & Smith, G.M. (2009a). GLM and GAM for Count Data. In: *Mixed effects models and extensions in ecology with R* (eds. Zuur, A.F., Ieno, E.N., Walker, N., Saveliev, A.A. & Smith, G.M.). Springer, New York, NY, pp. 209–243.

Zuur, A.F., Ieno, E.N., Walker, N.J., Saveliev, A.A. & Smith, G.M. (2009b). GLMM and GAMM. In: *Mixed effects models and extensions in ecology with R* (eds. Zuur, A.F., Ieno, E.N., Walker, N., Saveliev, A.A. & Smith, G.M.). Springer, New York, NY, pp. 323–341.
